# Correlation of tumor mutational burden with prognosis and immune infiltration in lung adenocarcinoma

**DOI:** 10.3389/fonc.2023.1128785

**Published:** 2023-03-07

**Authors:** Lin Li, Junyu Li

**Affiliations:** ^1^ Department of Thoracic Oncology, Jiangxi Cancer Hospital, Nanchang, China; ^2^ Department of Radiation Oncology, Jiangxi Cancer Hospital, Nanchang, China; ^3^ Jiangxi Health Committee Key (JHCK) Laboratory of Tumor Metastasis, Jiangxi Cancer Hospital, Nanchang, China

**Keywords:** lung adenocarcinoma, tumor mutation burden, immune checkpoint blockade, the cancer genome atlas, cell proliferation

## Abstract

**Background:**

Tumor mutational burden (TMB) plays an important role in the evaluation of immunotherapy efficacy in lung adenocarcinoma (LUAD).

**Objective:**

To improve the clinical management of LUAD by investigating the prognostic value of TMB and the relationship between TMB and immune infiltration.

**Methods:**

TMB scores were calculated from the mutation data of 587 LUAD samples from The Cancer Genome Atlas (TCGA), and patients were divided into low-TMB and high-TMB groups based on the quartiles of the TMB score. Differentially expressed genes (DEGs), immune cell infiltration and survival analysis were compared between the low-TMB and high-TMB groups. We queried the expression of genes in lung cancer tissues through the GEPIA online database and performed experimental validation of the function of aberrant genes expressed in lung cancer tissues.

**Results:**

We obtained sample information from TCGA for 587 LUAD patients, and the results of survival analysis for the high- and low- TMB groups suggested that patients in the high-TMB group had lower survival rates than those in the low-TMB group. A total of 756 DEGs were identified in the study, and gene set enrichment analysis (GSEA) showed that DEGs in the low-TMB group were enriched in immune-related pathways. Among the differentially expressed genes obtained, 15 immune-related key genes were screened with the help of ImmPort database, including 5 prognosis-related genes (CD274, PDCD1, CTLA4, LAG3, TIGIT). No difference in the expression of PDCD1, CTLA4, LAG3, TIGIT in lung cancer tissues and differential expression of CD274 in lung cancer tissues.

**Conclusions:**

The survival rate of LUAD patients with low TMB was better than that of LUAD patients with high TMB. CD274 expression was down regulated in human LUAD cell lines H1299, PC-9, A549 and SPC-A1, which inhibited malignant progression of A549 cells.

## 1 Introduction

The tumor mutation burden (TMB) refers to the number of somatic mutations per million bases in the coding region of tumor cells in a tumor sample. It is regarded as a biological marker of the level of tumor mutation (1). The number of neoantigens in a tumor correlates with TMB, and patients with high TMB are more likely to produce immunogenic neoantigens (2). Several clinical trials have shown a positive correlation between TMB and antigen recognition by T lymphocytes and the effectiveness of immunotherapy, which can be used to predict the efficacy of PD-1/PD-L1 inhibitors, such as in melanoma (3, 4). TMB is currently used as an indicator to assess the benefit of LUAD patients from ICI therapy (5). As a factor to assess the prognosis of LUAD patients after receiving ICI, several clinical trials have shown a positive correlation between TMB and T lymphocyte recognition of antigens and the effectiveness of immunotherapy (5–7).

As a relatively common subtype of lung cancer worldwide, patients with lung adenocarcinoma (LUAD) have poor survival rates and poor prognosis (8, 9). It is estimated that there are 2.09 million new lung cancer cases and 1.76 million lung cancer deaths each year, with LUAD accounting for approximately 40% of all lung cancer cases in the world (10–12). With advances in technology, surgical resection, immunotherapy and targeted therapy offer a variety of treatment options for LUAD patients, but due to the highly heterogeneous nature of LUAD, the 5-year survival rate for patients ranges from 4% to 17% (5, 13, 14). Finding effective treatment modalities to improve the survival rate and improve the survival outcome of LUAD patients is the main direction of current research. Current first-line treatment (EGFR-TKI therapy) for patients with LUAD in lung adenocarcinoma has yielded good results, but most LUAD patients eventually acquire drug resistance (15). Immune checkpoint blockade (ICB) therapy is currently considered to be an effective treatment for patients with LUAD (16, 17). The main principle of action of ICB for tumor treatment is to target the immune recognition and immune response related escape mechanism of tumor cells. (PD-L1) antibodies, tumor cells are now being tested for the expression of PD-L1. PD-L1 expressed on tumor cells has been listed by the FDA as a companion or complementary diagnosis for screening lung cancer patients treated with PD-1/PD-L1 treatment population as a concomitant or complementary diagnosis (18, 19). However, studies have shown that PD-L1 as a biomarker has some limitations and still needs to be combined with other biomarkers. However, studies have shown that PD-L1 as a biomarker has some limitations and still needs to be combined with other evidence (20).

We believe that combined research on TMB and immunotherapy is beneficial to decipher the limitations of PD-L1 as a biomarker. However, there is no uniform conclusion on the mechanism of how TMB affects the efficacy and prognosis of immunotherapy. In the present study, we intend to further investigate the mechanisms by which TMB affects prognosis by exploring the relationship between TMB, immune cell infiltration and prognosis in patients with LUAD.

## 2 Materials and methods

### 2.1 Data source and data processing

We used the “TCGAbiolinks” R package to extract clinical information (age, gender, TNM stage, overall survival (OS), progression-free survival (PFS), etc.) and mutation profiling of LUAD patients from the TCGA database (https://portal.gdc.cancer.gov/) (21). The mutation data processing process involves GDCquery and GDCprepare (22). We used the “maftools” R package to visualize the MAF files (23). Data cleaning was performed by R software (vision4.2.2), e.g. by “dplry” (24) and “stringr”[B]. In addition, we obtained IRG lists from the immunology database and analysis portal ImmPort (https://immport.niaid.nih.gov) (25).

### 2.2 TMB calculation and analysis

We calculated the TMB of the samples using the “maftools” R package (the pipeline of MAF file: muse) (23). The TMB was calculated as TMB= (number of somatic mutations)/(length of sample CDS region), CDS (Coding sequence) refers to the protein coding region sequence. We divided the LUAD samples into high and low TMBs based on the quartiles of TMB scores. The lower 25% of the scores were defined as the low TMB group, while the higher 25% of the scores were defined as the high TMB group.

### 2.3 Survival analysis of high- and low- TMB groups

We assessed the effect of high and low TMB on OS and PFS in LUAD patients by Kaplan-Meier method. In addition, we compared the impact of different clinical baseline characteristics on the prognosis of patients in the high and low TMB groups. The Wilcoxon test was used to analyze the differences between the two groups, and samples with missing values were excluded from the analysis. The image editing software was GraphPad Prism **8.2.1 (** 25).

### 2.4 Screening for differently expressed genes and survival-related immune genes

We chose to detect differentially expressed genes (DEGs) in both high- and low- TMB groups using “Limma” (26). We set the fold change not to log (fold change)>1 to reduce the effect of confounding factors and performed multiple testing correction to control the false discovery rate (FDR<0.05) (27). We compared DEGs with IRG list genes to screen for immune-related differentially expressed genes. Bulk survival analysis of sample transcriptome data combined with clinical information data was performed by R software to obtain survival-associated genes. Survival-related genes were compared with immune-related differentially expressed genes to screen for survival-related immune genes.

### 2.5 Cell culture and transfection

Human LUAD cell lines (H1299, PC-9, A549 and SPC-A1) and normal human lung epithelial cell lines (HBE) were purchased from the Cell center of Chinese Academy of Sciences, Shanghai, China. Cells were cultured in RPMI1640 (Thermo Fisher Scientific, MA, USA) containing 10% fetal bovine serum (FBS) (Thermo Fisher Scientific, MA, USA) and 1% penicillin/streptomycin (Invitrogen, CA, USA) in an incubator at 37°C with 5% CO2 volume fraction. The cells were digested with 0.25% trypsin (Thermo Fisher HyClone, Utah, USA) during the logarithmic growth phase.

The pcDNA empty vector (NC), pcDNA-CD274 (CD274) were purchased from GenePharma Co., Ltd. (Shanghai, China). A549 cells were inoculated at a density of 3×105 cells/well in a 24-well cell culture plate and incubated at 37°C with 5% CO2 for 24h before cell transfection. A549 cells were transfected with Lipofectamine ^®^ 3000 (Invitrogen; ThermoFisherScientific, Inc.) according to the supplier’s instructions. Transfection efficiency was assayed by quantitative reverse transcription-polymerase chainreaction (qRT-PCR). Cells were incubated at 37˚C and 5% CO2 for 24h, pending further analysis.

### 2.6 qRT-qPCR and CCK8

Total RNA from human LUAD cell lines (H1299, PC-9, A549 and SPC-A1) and normal human lung epithelial cell lines (HBE) was extracted with TRIzol reagent (Invitrogen, Carlsbad, CA, USA), tested for purity and then reverse transcribed into cDNA using RevertAid First Strand cDNA Synthesis Kit (Thermo Fisher Scientific, Waltham, MA, USA). We performed quantitative reverse transcription-polymerase chainreaction (qRTPCR) using SYBR^®^Premix-Ex-Taq™ (Takara, TX, USA) and ABI7300 system. The expression level of CD274 (after correction of GAPDH as an internal reference) was assessed using the 2-ΔΔCt method (28). CD274: Forward, 5’-CATCTTATTATGCCTTGGTGTAGCA-3, Reverse, 5’- GGATTACGTCTCCTCCAAATG TG-3’; GAPDH: Forward, 5’-GAACGGGAAGCTCACTGG-3’, Reverse, 5’- GCCTGCTTCACCACCTTCT-3’. It was found that the expression of CD274 was most significantly down-regulated in A549 cells. We performed the follow-up experiment in A549 cells.

2×103 cells of each group were taken and inoculated in 96-well plates. After 24 h of wall incubation, 10 μL of CCK-8 solution (Dojindo Molecular Technologies, Kumamoto, Japan) was added to each well. The plates were incubated in the incubator for 4h, and the absorbance values at 450nm were measured using an enzyme marker. The changes in cell proliferation capacity of A549 cells at 24, 48 and 72 h after transfection as well as cotransfection were measured in this way, respectively. The experiment was repeated three times and measured three times.

### 2.7 Transwell assay for invasion and flow cytometry for apoptosis

The invasiveness of A549 cells was assessed by Transwell assay. Transwell chambers (Corting, NY, USA) were coated with 200 mg/ml Matrigel (BD, SanJose, USA) and incubated overnight. A549 cells were then added to the upper chamber of serum-free medium. DMEM (500 μl) containing 10% FBS was placed in the lower chamber as a chemotactic agent. After 24h incubation, all non-invasive cells were removed. Matrigel membranes were fixed with paraformaldehyde and then stained with crystalline violet solution. The number of invading cells was counted using a phase contrast microscope (Olympus, Tokyo, Japan). The experiment was repeated three times and measured three times.

For the apoptosis assay, follow the steps in the instructions of the Apoptosis Detection Kit (Shanghai Aladdin Bioreagents): after washing A549 cells twice with PBS, 400 μl of pre-cooled PBS was added, followed by 10 μl AnnexinV-FITC and 5 μl PI, respectively, and incubated for 30 min at 4°C protected from light, and then immediately measured by flow cytometry (model: Becton- Dickinson) and the percentage of apoptotic cells was calculated after processing by computer software. The experiment was repeated three times and measured three times.

## 3 Results

### 3.1 Landscape of mutation profiles in LUAD samples

We downloaded mutation data from TCGA for 587 LUAD samples and merged them with clinical information based on TCGA sample IDs. The clinical information of the 522 LUAD patients after removal of the missing values was shown in **Table 1**. The mean age of the patients was 57.85 years and the male to female ratio was 0.86:1. We used the quartile method to perform TMB calculations on the patient sample information (Q1 = 1.240, Q2 = 3.170, Q3 = 6.375, Q4 = 37.540).

**Table 1:** Clinical information of 522 LUAD patients.

Variables		Variables	
Age(y)	57.85 ± 6.44	Stage	
Gender		I	279 (53.45)
Female	280 (53.64)	II	124 (23.75)
Male	242 (46.36)	III	85 (16.28)
AJCC-T		IV	34 (6.52)
T1	172 (32.95)	Status	
T2	281 (54.60)	Dead	188 (36.02)
T3	47 (9.00)	Alive	334 (63.98)
T4	19 (3.64)	TMB	
TX	3 (0.57)	TMB>6.38	362
AJCC-N		TMB ≤ 1.24	110
N0	335 (64.18)	AJCC-M	
N1	98 (18.77)	M0	353 (67.62)
N2	75 (14.37)	M1	25 (4.790)
N3	2 (0.38)	MX	144 (27.59)
NX	12 (2.30)		

Categorical variables are expressed as percentages and continuous variables as SD±χ.

**Figure 1:**
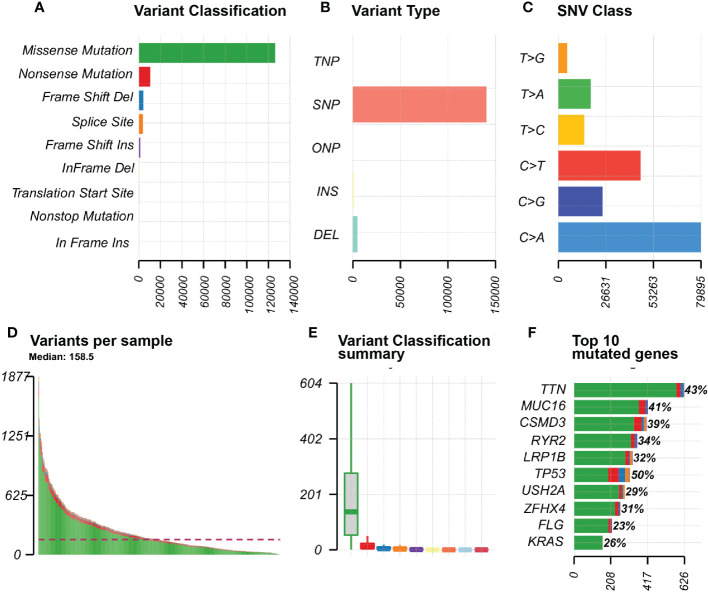
Mutation information in LUAD samples SNP, single nucleotide polymorphism; SNV, single nucleotide variant. **(A)** Variant Classification of genes in the sample; **(B)** Variant Type of the gene in the sample; **(C)** SNV Class of the gene in the sample; **(D)** Graph of variants for each sample; **(E)** Summary chart of variant classification; **(F)** Top 10 mutated genes in the sample.

We visualised the mutation profile of LUAD patients from TCGA *via* the “maftools “R package in **Figure 1**. The largest proportion of mutation types were missense mutations (**Figure 1A**). **Figure 1B** reflected that single nucleotide polymorphisms occur significantly more frequently than insertions or deletions, and **Figure 1C** showed that the most common type of single nucleotide variation is C>T. T; **Figure 1D** presented the number of base mutations in each sample. **Figure 1E** showed a summary of the different mutation types. **Figure 1F** showed the top 10 mutated genes in the samples, including TTN, MUC16, CSMD3, RYR2, LRP1B, TP53, USH2A, ZFHX4, FLG, KRAS, etc.

**Figure 2:**
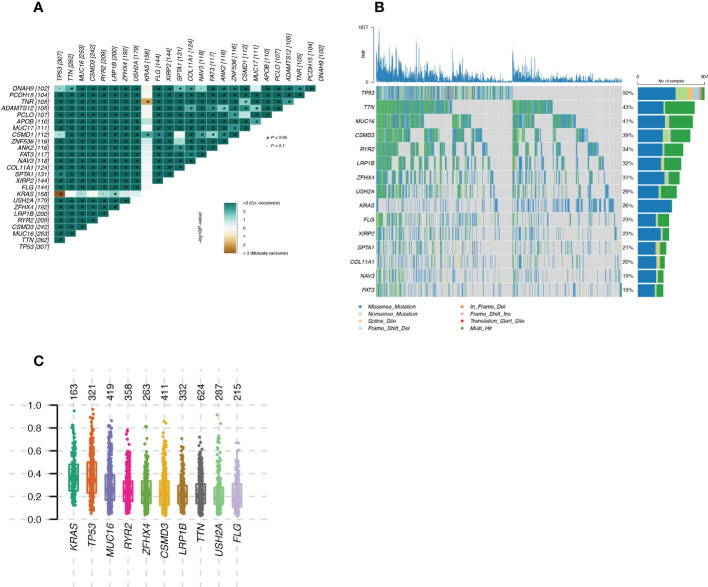
Landscape of mutated genes in LUAD samples from TCGA **(A)**: Mutually exclusive mutations or simultaneous mutations in the sample; **(B)**: Waterfall plot of gene mutations in the sample; **(C)**: Box plot of Variant Allele Frequencies (VAF) reflecting gene cloning status.

The mutational landscape of LUAD patients from TCGA was shown in **Figure 2**. **Figure 2A** showed the mutually exclusive mutations and concurrent mutations present in the top 15 mutated genes in the sample. Green represented concurrent occurrence and tan represents mutual exclusion. The magnitude of the P-value for the correlation test was indicated by the shade of the color, the darker the color the smaller the P-value, the greater the confidence in the correlation test for the corresponding two genes; the lighter the color the larger the P-value, the lower the confidence in the correlation test for the corresponding two genes. Plotting the Variant Allele Frequencies (VAF) of the genes (**Figure 2C**) gave an indication of the clonal status of the genes. Ideally, the average allele frequency of the cloned genes in the sample was approximately 50%.

### 3.2 Survival analysis of patients in the high- and low TMB- groups

**Figure 3:**
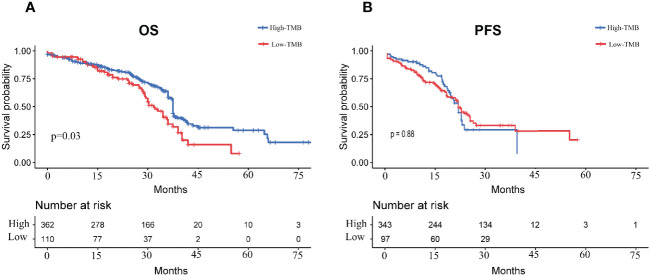
Survival curves of LUAD patients in high- and low- TMB groups **(A)**: OS survival curves of LUAD patients in high and low TMB groups; **(B)**: PFS survival curves of LUAD patients in high and low TMB groups.

**Figure 3A** illustrated the trend of OS survival over time for patients in the high- and low- TMB groups. The blue survival curve represented the lower TMB group and the red survival curve represented the higher TMB group. **Figure 3B** showed the trend of PFS survival over time for patients in the high- and low- TMB groups. A Log Rank test showed a difference in OS survival between the two groups (p=0.03), while there was no significant difference in PFS survival (p=0.88).

### 3.3 Differentially expressed genes and prognostic immune-related genes

**Figure 4:**
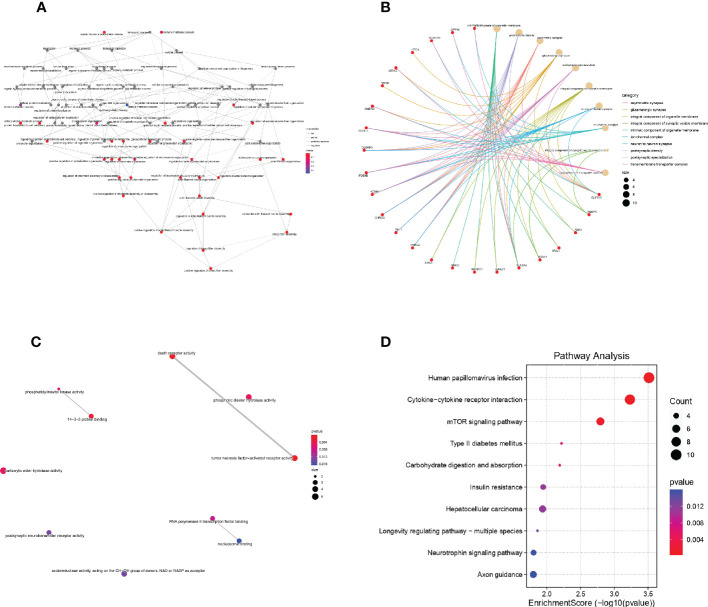
GO and KEGG analysis of DEGs **(A)**: Biological process of DEGs in Gene Ontology; **(B)**: Cellular component of DEGs in Gene Ontology; **(C)**: molecular function of DEGs in Gene Ontology; **(D)**: KEGG analysis of DEGs.

The results of GO analysis and KEGG analysis of the differential genes were shown in **Figure 4**. **Figure 4A** showed the BP pathway for the differential genes, **Figure 4B** showed the CC pathway for the differential genes, **Figure 4C** showed the MF pathway for the differential genes, and **Figure 4D** showed the KEGG pathway for the differential genes.

**Figure 5:**
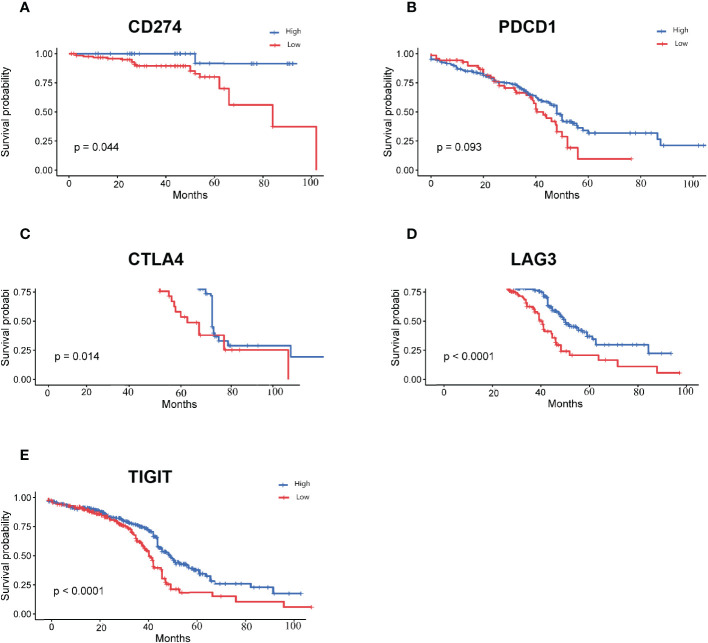
Survival curves for high and low expression groups of immune-associated differentially expressed genes **(A)**: Survival curves for high and low expression groups of CD274; **(B)**: Survival curves for high and low expression groups of PDCD1; **(C)**: Survival curves for high and low expression groups of CTLA4; **(D)**: Survival curves for high and low expression groups of LAG3; **(E)**: Survival curves for high and low expression groups of TIGIT.

The 756 DEGs were compared with the list of genes downloaded from ImmPort, and 15 immune-related differentially expressed genes common to both groups were screened. We performed survival analysis of the screened genes using the Kaplan-Meier plotter, and five of them (CD274, PDCD1, CTLA4, LAG3, and TIGIT) were significantly correlated with patient survival (P<0.05). The survival curves for the high- and low- expression groupings of the immune-related differentially expressed genes were shown in **Figure 5**.

### 3.4 Cellular assays section

**Figure 6:**
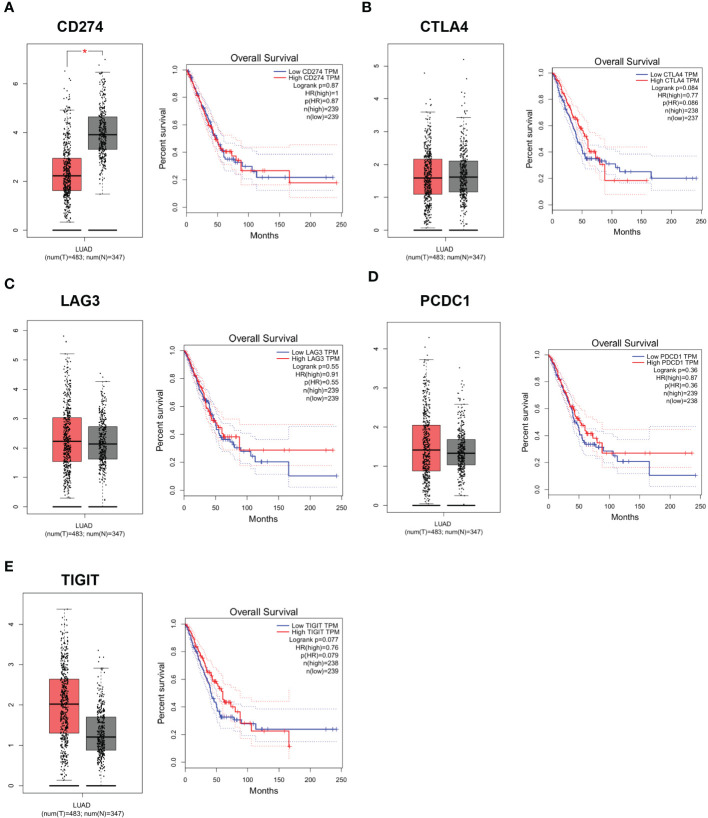
Expression levels and survival curves of genes in LUAD analyzed by GEPIA online database. **(A)**: Expression levels and survival curves of CD274 in LUAD analyzed by GEPIA online database; **(B)**: Expression levels and survival curves of CTLA4 in LUAD analyzed by GEPIA online database; **(C)**: Expression levels and survival curves of LAG3in LUAD analyzed by GEPIA online database; **(D)**: Expression levels and survival curves of PCDC1 in LUAD analyzed by GEPIA online database; **(E)**: Expression levels and survival curves of TIGIT in LUAD analyzed by GEPIA online database.

CD274 expression was down-regulated in LUAD tumor tissues in the GEPIA database. To further assess the role of DEG in LUAD, we used the GEPIA online database (http://gepia.cancer-pku.cn) to analyze the expression levels of CD274, TIGIT, PDCD1, CTLA4 and LAG3 in normal population and tumor tissues of LUAD patients, as well as the relationship between each gene and LUAD patients. The relationship between the expression levels of CD274, TIGIT, PDCD1, CTLA4 and LAG3 in tumor tissues of normal and LUAD patients and the relationship between each gene and the survival rate of LUAD patients. CD274 expression was found to be down-regulated in tumor tissues (P < 0.05), while TIGIT, PDCD1, CTLA4 and LAG3 expression in tumor tissues were not statistically different (**Figures 6A–E**). This indicated that there was an abnormal expression profile of CD274 expression levels in the tumor samples.

### 3.5 CD274 expression is down-regulated in human LUAD cell lines

**Figure 7:**
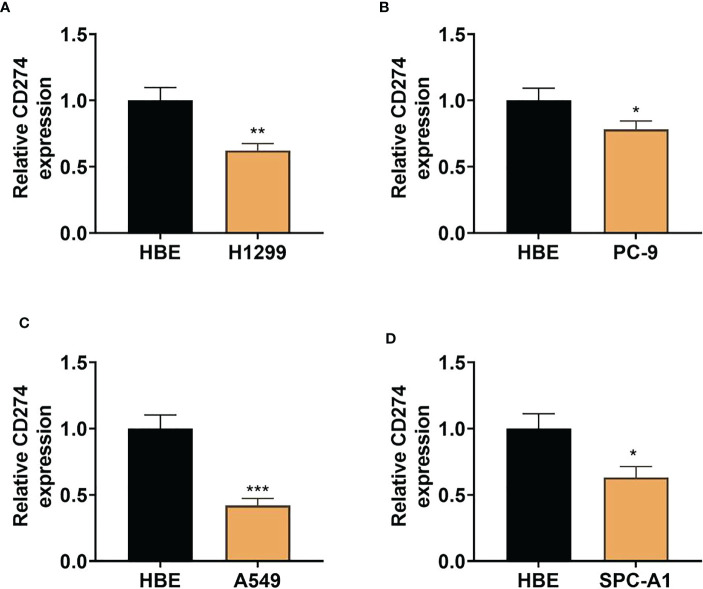
Expression of CD274 in human LUAD cell lines (H1299, PC-9, A549, and SPC-A1) and normal human lung epithelial cell lines (HBE) **(A–D)**: qRT-PCR to detect CD274 expression in human LUAD cell lines (H1299, PC-9, A549, and SPC-A1) after transfection with pcDNA empty vector and pcDNACD274. *P < 0.05 **P < 0.01 and ***P < 0.001 as determined by two-tailed t-test.

To further confirm whether CD274 expression is dysregulated in LUAD, we examined CD274 expression in human LUAD cell lines (H1299, PC-9, A549 and SPC-A1) and normal human lung epithelial cell lines (HBE) using qRT-PCR. The results showed that the expression of CD274 was significantly down-regulated in human LUAD cell lines H1299, PC-9, A549 and SPC-A1 compared to normal human lung epithelial cell line HBE, with the most significant down-regulation in A549 cells (P < 0.05, **Figures 7A–D**). The above results indicated that CD274 expression was down-regulated in human LUAD cell lines H1299, PC-9, A549 and SPC-A1.

### 3.6 Upregulation of CD274 inhibits proliferation and invasion and increases apoptosis in A549 cells

**Figure 8:**
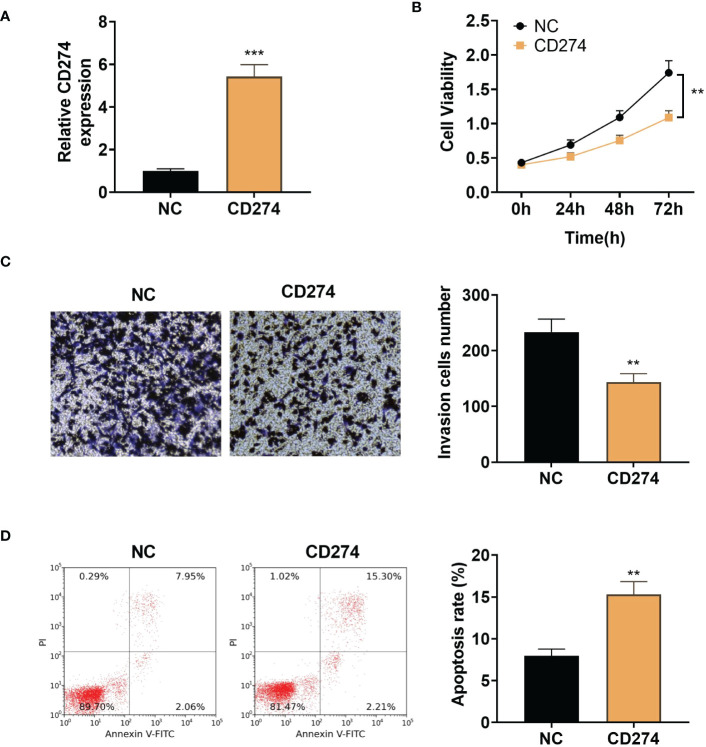
Up-regulation of CD274 inhibits proliferation and invasion and increases apoptosis in A549 cells **(A)**: qRT-PCR assay of CD274 expression in A549 cells. **(B)**: CCK8 assay of A549 cell proliferation. **(C)**: Transwell assay of A549 cell invasion. **(D)**: Flow cytometry detection of apoptosis in A549 cells. **P < 0.01 and ***P < 0.001 as determined by two-tailed t-test.

To examine the effect of CD274 in A549 cells, we transfected pcDNA empty vector (NC) and pcDNA-CD274 (CD274) into A549 cells and detected the expression of CD274 in A549 cells after 24h using qRT-PCR. CD274 expression was found to be significantly up-regulated in cells transfected with pcDNA-CD274 compared to the NC group, indicating successful transient transfection (P < 0.05, **Figure 8A**). CCK8 assays showed that up-regulation of CD274 significantly reduced cell viability in A549 cells compared to NC (P < 0.05, **Figure 8B**). In addition, we explored whether CD274 was involved in cell invasion in A549 cells. Transwell assays showed that the number of invading cells was significantly reduced in the CD274 group compared to the NC group (P < 0.05, **Figure 8C**). Similarly, flow cytometry showed that CD274 significantly promoted apoptosis in A549 cells compared to the NC group (P < 0.05, **Figure 8D**). The above results suggested that CD274 inhibited the malignant progression of A549 cells.

## 4 Discussion

LUAD accounts for approximately 40% of all lung cancer cases (29), and finding appropriate treatment modalities to improve survival outcomes in patients with LUAD is a problem that needs to be addressed by current medicine. Patients with LUAD were often treated with adjuvant therapies such as radiotherapy, chemotherapy and targeted therapy. EGFR-TKI therapy as the first-line treatment for patients with LUAD has achieved excellent results in the initial stage, but the development of drug resistance in patients at a later stage is also a non-negligible situation (15). The ICB has demonstrated significant overall survival and progression-free survival benefits with LUAD, but there was inter-patient heterogeneity in the efficacy of immunotherapy (30–33). TMB was a recently discovered independent biomarker for predicting the efficacy of immunotherapy, and its predictive ability in immunotherapy was not limited to “hot tumors” such as lung adenocarcinoma (34), non-small cell lung cancer (35), and melanoma (36), but also as a biomarker for other cancers (37, 38). At the same time, TMB results can effectively taken into account the heterogeneity of the samples. Recently researches have shown that patients with a high number of somatic mutations benefit more from immune checkpoint inhibitor therapy (1). However, the prognostic value of TMB and the relationship between TMB and immune infiltration in LUAD have been less studied in LUAD. We conducted this study to provide data to support the improvement of clinical immunotherapy efficacy in patients with LUAD.

In a tumor mutational load study of lung adenocarcinoma, Lv Y et al (39) defined high TMB levels as ≥10 mutations per MB and low TMB levels as <10 mutations per MB (40).We used the quartile method to perform TMB calculations on the patient sample information (Q1 = 1.240, Q2 = 3.170, Q3 = 6.375, Q4 = 37.540). LUAD patients with TMB values lower than Q1 were classified as the low-TMB group, and LUAD patients with TMB values higher than Q3 were classified as the high-TMB group. During the study of tumor mutation load and prognosis of patients with LUAD, Wu D et al (41) divided patients into high TMB (>maximum 25%), medium TMB, and low TMB (<minimum 25%) groups according to their TMB levels. This is consistent with our grouping approach, and we believe that this TMB grouping increases the precision of the study and the significance of the results.

Our results showed that patients in the high-TMB group had lower survival rates than those in the low-TMB group. Meanwhile numerous studies have explored the immune subtypes of LUAD and their relationship with clinical response to immune checkpoint inhibitors (42–46).Wang S et al. (7) downloaded information on patients with LUAD from the TCGA database, and performed tumor molecular subtype, association analysis and independent validation cohort validation. They concluded that patients with high-risk LUAD were characterized by higher TMB. Their findings showed that high-risk LUAD patients were characterized by higher TMB, and the high-risk group was associated with poorer survival outcomes. The findings of Wang S et al. were consistent with our study, which laterally showed that our findings could provide data to support the prognosis prediction of LUAD patients.

In a series of studies correlating immune function with survival outcome in LUAD patients, Seo et al. (47) concluded that the subtype of LUAD patients with normal immune function was characterized by elevated expression of immune checkpoint genes, however the differential survival outcome was not significant compared to LUAD patients with abnormal immune function. Wang W (48) and Qi YA et al. (49) downloaded LUAD patient data from the TCGA database and GEO database,which was supplemented by an in-depth study in the direction of Seo et al. It was concluded that immune checkpoint inhibitors showed no significant therapeutic effect in cancers with low TMB, such as mutant epidermal growth factor receptor (EGFR)-driven lung adenocarcinoma. This is discrepant from our findings and speculation is that there may be a correlation with patient sample differences and TMB subgroup differences.

To clarify the reasons for the differences in immunotherapy efficacy in LUAD patients, Niu Y et al. (50) performed a mutated gene screen in the TCGA-LUAD cohort,and found that NTRK3 mutations were strongly associated with immunotherapy. They concluded that that there were significant differences in survival rates between patients with the two mutation types (mutated NTRK3, NTRK3-MT). Although the relevance of TMB to immunotherapy was not the focus of this study, the results of this study showed an association between the mutation, TMB and the outcome of immunotherapy in patients with LUAD. Jia Q et al (51) also investigated the correlation between TTN mutations and the efficacy of immunotherapy and concluded that TTN mutation status independently predicted immunotherapy prognosis. This may be related to down-regulation of pathways associated with immunosuppression and immune depletion. Our mutation data results showed TTN as a significant mutational factor and TTN-MT as a potential predictive marker for patients with LUAD receiving ICI still needs to be validated by a large body of evidence.

Our results showed that the pathways affected by DEGs were mainly associated with immune, inflammatory progression, which is consistent with the results of several studies. For example, bioinformatics analysis by Li X et al. concluded that the expression of IRRGs was significantly associated with TMB. They also concluded that the lncRNA MIR503HG/SNHG17/miR-330-3p/regulatory axis involving altered lncRNAs was significantly associated with immune cell infiltration (52). We also suggested that upregulation of CD274 inhibited proliferation and invasion of A549 cells and increased apoptosis in lung cancer cells. The CD274 gene encodes PD-L1, a major co-inhibitory checkpoint signal that controls T-cell activity (53, 54). A study showed a significant increase in PD-L1 in shGBE1 A549 cells and a negative correlation between PD-L1 and GBE1 (55).

There were some limitations to this research. The first was that the sample size was not sufficient, making the results potentially unbalanced and incomplete, etc. Secondly, we lower 25% and higher 25% TMB as our cut off this leaves out a category between the two cut-offs, which may leaded to some limitations and incompleteness of the results. Finally we did not perform correlation analysis of baseline factors due to insufficient sample size and more missing clinical information. As the differences in TMB and its impact on the prognosis of lung adenocarcinoma between raceswere still unknown, we intended to conduct a large cohort study in the next step to refine the data in this area and provided a reference for the treatment of lung adenocarcinoma.

## 5 Conclusions

The survival of LUAD patients with low TMB was better than that of LUAD patients with high TMB. CD274, PDCD1, CTLA4, LAG3, TIGIT and LUAD prognosis were associated. CD274 expression was down regulated in human LUAD cell lines H1299, PC-9, A549 and SPC-A1 and inhibits malignant progression in A549 cells.

## Data availability statement

The original contributions presented in the study are included in the article/**Supplementary Material**. Further inquiries can be directed to the corresponding author.

## Author contributions

LL and JL contributed to the conception and design of the study. LL organized the database and statistical analyses and wrote the first draft of the manuscript. LL and JL wrote parts of the manuscript. All authors contributed to the article and approved the submitted version.
