# Supplementary figures and images for: Correlation of tumor mutational burden with prognosis and immune infiltration in lung adenocarcinoma
Source: Front Oncol. 2023 Mar 7;13:1128785. doi: 10.3389/fonc.2023.1128785 (PMC10028277; doi:10.3389/fonc.2023.1128785)

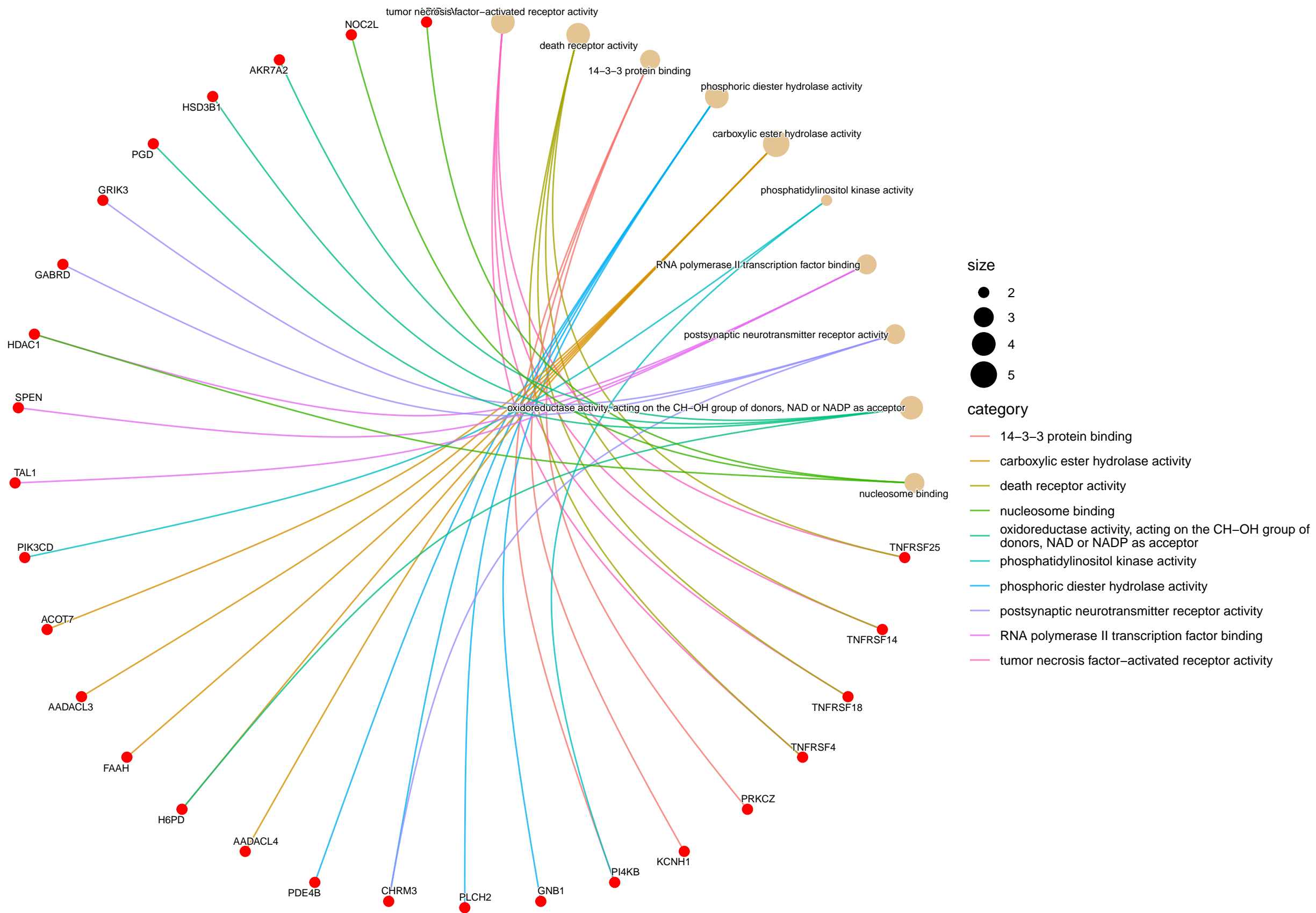

Supplement: Supplementary file 7 [file DataSheet_7.pdf]

## Slide 1
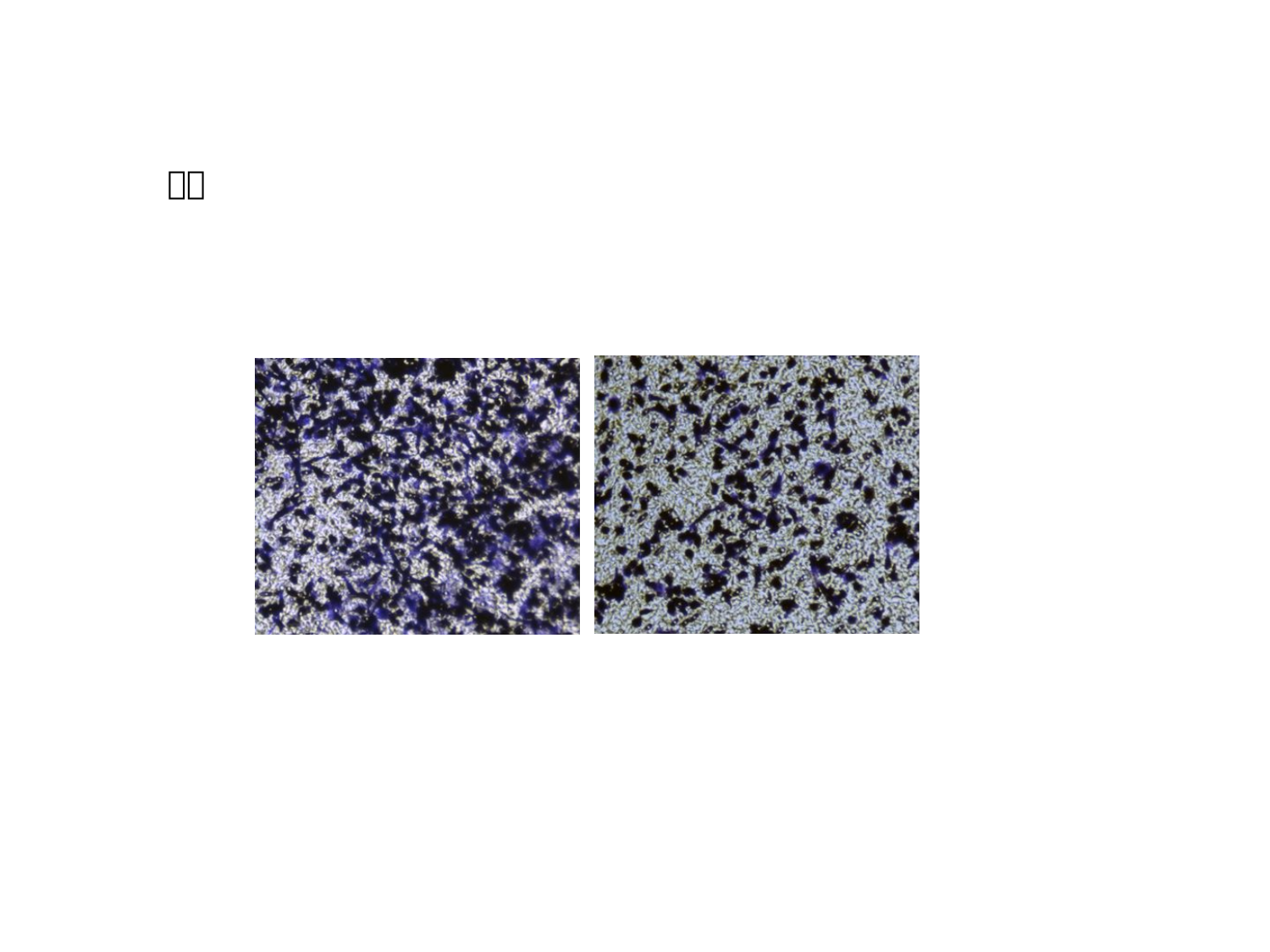

侵袭

## Slide 2
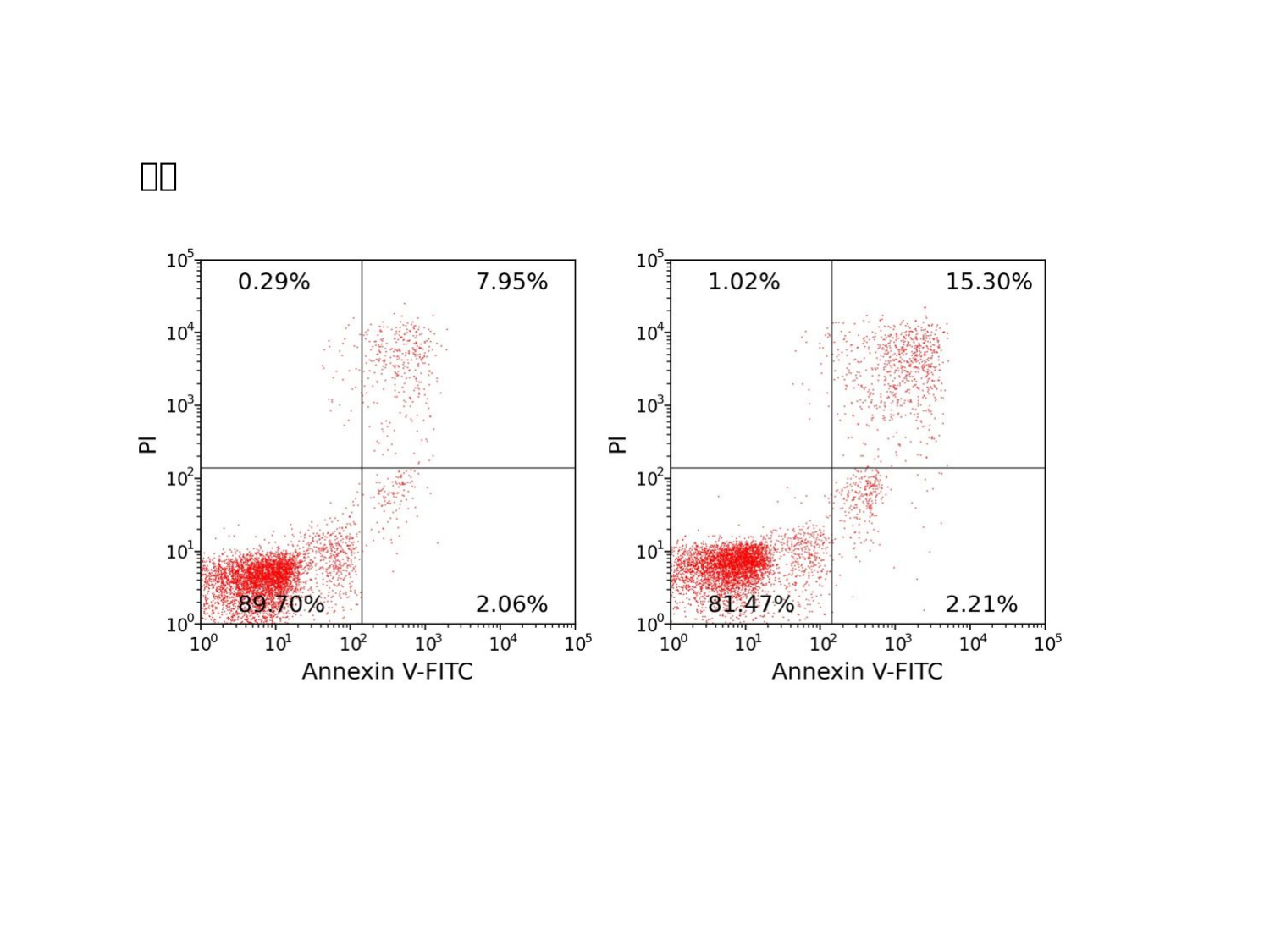

凋亡

## Slide 3
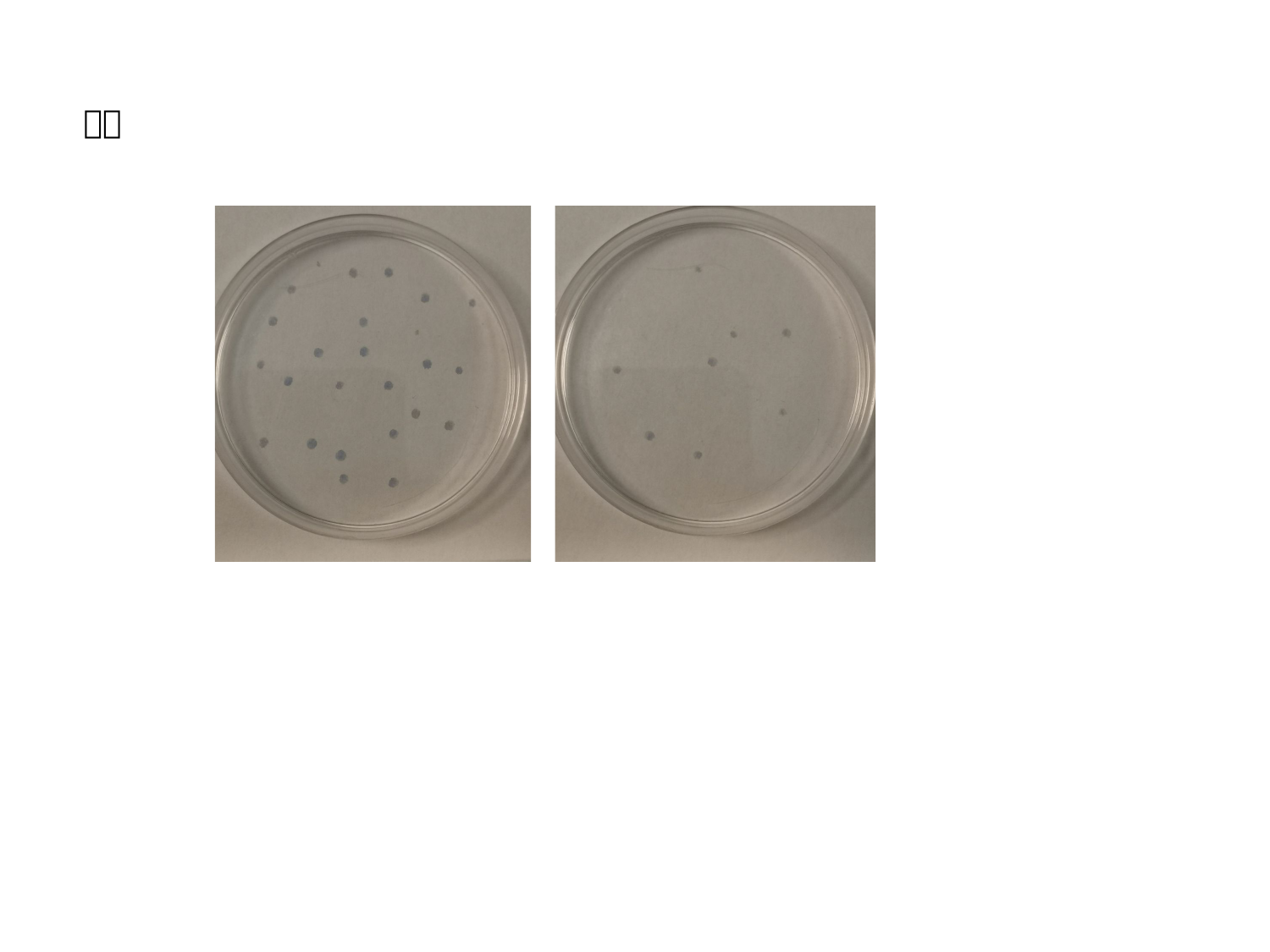

克隆

Supplement: Supplementary file 13 [file Presentation_1.ppt]
