# Supplementary material for: Correlation of tumor mutational burden with prognosis and immune infiltration in lung adenocarcinoma
Source: Front Oncol. 2023 Mar 7;13:1128785. doi: 10.3389/fonc.2023.1128785 (PMC10028277; doi:10.3389/fonc.2023.1128785)
Supplement: Supplementary file 8 [file Table_1.docx]

Symbol ID Name Synonyms Chromosome Category

AZGP1 563 alpha-2-glycoprotein 1, zinc-binding ZA2G|ZAG 7 Antigen_Processing_and_Presentation

B2M 567 beta-2-microglobulin IMD43 15 Antigen_Processing_and_Presentation

CALR 811 calreticulin CRT|HEL-S-99n|RO|SSA|cC1qR 19 Antigen_Processing_and_Presentation

CANX 821 calnexin CNX|IP90|P90 5 Antigen_Processing_and_Presentation

CD1A 909 CD1a molecule CD1|FCB6|HTA1|R4|T6 1 Antigen_Processing_and_Presentation

CD1B 910 CD1b molecule CD1|CD1A|R1 1 Antigen_Processing_and_Presentation

CD1C 911 CD1c molecule BDCA1|CD1|CD1A|R7 1 Antigen_Processing_and_Presentation

CD1D 912 CD1d molecule CD1A|R3|R3G1 1 Antigen_Processing_and_Presentation

CD1E 913 CD1e molecule CD1A|R2 1 Antigen_Processing_and_Presentation

CD4 920 CD4 molecule CD4mut 12 Antigen_Processing_and_Presentation

CD8A 925 CD8a molecule CD8|Leu2|p32 2 Antigen_Processing_and_Presentation

CD8B 926 CD8b molecule CD8B1|LEU2|LY3|LYT3|P37 2 Antigen_Processing_and_Presentation

CD74 972 CD74 molecule DHLAG|HLADG|II|Ia-GAMMA|p33 5 Antigen_Processing_and_Presentation

CREB1 1385 cAMP responsive element binding protein 1 CREB|CREB-1 2 Antigen_Processing_and_Presentation

CTSB 1508 cathepsin B APPS|CPSB|RECEUP 8 Antigen_Processing_and_Presentation

CTSE 1510 cathepsin E CATE 1 Antigen_Processing_and_Presentation

CTSL 1514 cathepsin L CATL|CTSL1|MEP 9 Antigen_Processing_and_Presentation

CTSS 1520 cathepsin S - 1 Antigen_Processing_and_Presentation

FCER1G 2207 Fc fragment of IgE receptor Ig FCRG 1 Antigen_Processing_and_Presentation

FCGRT 2217 Fc fragment of IgG receptor and transporter FCRN|alpha-chain 19 Antigen_Processing_and_Presentation

PDIA3 2923 protein disulfide isomerase family A member 3 ER60|ERp57|ERp60|ERp61|GRP57|GRP58|HEL-S-269|HEL-S-93n|HsT17083|P58|PI-PLC 15 Antigen_Processing_and_Presentation

HFE 3077 homeostatic iron regulator HFE1|HH|HLA-H|MVCD7|TFQTL2 6 Antigen_Processing_and_Presentation

HLA-A 3105 major histocompatibility complex, class I, A HLAA 6 Antigen_Processing_and_Presentation

HLA-B 3106 major histocompatibility complex, class I, B AS|B-4901|HLAB 6 Antigen_Processing_and_Presentation

HLA-C 3107 major histocompatibility complex, class I, C D6S204|HLA-JY3|HLAC|HLC-C|MHC|PSORS1 6 Antigen_Processing_and_Presentation

HLA-DMA 3108 major histocompatibility complex, class II, DM alpha D6S222E|DMA|HLADM|RING6 6 Antigen_Processing_and_Presentation

HLA-DMB 3109 major histocompatibility complex, class II, DM beta D6S221E|RING7 6 Antigen_Processing_and_Presentation

HLA-DOA 3111 major histocompatibility complex, class II, DO alpha HLA-DNA|HLA-DZA|HLADZ 6 Antigen_Processing_and_Presentation

HLA-DOB 3112 major histocompatibility complex, class II, DO beta DOB|HLA_DOB 6 Antigen_Processing_and_Presentation

HLA-DPA1 3113 major histocompatibility complex, class II, DP alpha 1 DP(W3)|DP(W4)|DPA1|HLA-DP1A|HLA-DPB1|HLADP|HLASB|PLT1 6 Antigen_Processing_and_Presentation

HLA-DPB1 3115 major histocompatibility complex, class II, DP beta 1 DPB1|HLA-DP|HLA-DP1B|HLA-DPB 6 Antigen_Processing_and_Presentation

HLA-DQA1 3117 major histocompatibility complex, class II, DQ alpha 1 CELIAC1|DQ-A1|DQA1|HLA-DQA 6 Antigen_Processing_and_Presentation

HLA-DQA2 3118 major histocompatibility complex, class II, DQ alpha 2 DC-alpha|DX-ALPHA|HLA-DCA|HLA-DXA|HLADQA2 6 Antigen_Processing_and_Presentation

HLA-DQB1 3119 major histocompatibility complex, class II, DQ beta 1 CELIAC1|HLA-DQB|IDDM1 6 Antigen_Processing_and_Presentation

HLA-DRA 3122 major histocompatibility complex, class II, DR alpha HLA-DRA1 6 Antigen_Processing_and_Presentation

HLA-DRB1 3123 major histocompatibility complex, class II, DR beta 1 DRB1|HLA-DR1B|HLA-DRB|SS1 6 Antigen_Processing_and_Presentation

HLA-DRB3 3125 major histocompatibility complex, class II, DR beta 3 DRB3|HLA-DPB1|HLA-DR1B|HLA-DR3B 6 Antigen_Processing_and_Presentation

HLA-DRB4 3126 major histocompatibility complex, class II, DR beta 4 DR4|DRB4|HLA-DR4B|HLA-DRB4* 6 Antigen_Processing_and_Presentation

HLA-DRB5 3127 major histocompatibility complex, class II, DR beta 5 - 6 Antigen_Processing_and_Presentation

HLA-E 3133 major histocompatibility complex, class I, E HLA-6.2|QA1 6 Antigen_Processing_and_Presentation

HLA-F 3134 major histocompatibility complex, class I, F CDA12|HLA-5.4|HLA-CDA12|HLAF 6 Antigen_Processing_and_Presentation

HLA-G 3135 major histocompatibility complex, class I, G MHC-G 6 Antigen_Processing_and_Presentation

HLA-H 3136 major histocompatibility complex, class I, H (pseudogene) HLAHP 6 Antigen_Processing_and_Presentation

MR1 3140 major histocompatibility complex, class I-related HLALS 1 Antigen_Processing_and_Presentation

HSPA1A 3303 heat shock protein family A (Hsp70) member 1A HEL-S-103|HSP70-1|HSP70-1A|HSP70-2|HSP70.1|HSP70.2|HSP70I|HSP72|HSPA1 6 Antigen_Processing_and_Presentation

HSPA1B 3304 heat shock protein family A (Hsp70) member 1B HSP70-1|HSP70-1B|HSP70-2|HSP70.1|HSP70.2|HSP72|HSPA1|HSX70 6 Antigen_Processing_and_Presentation

HSPA1L 3305 heat shock protein family A (Hsp70) member 1 like HSP70-1L|HSP70-HOM|HSP70T|hum70t 6 Antigen_Processing_and_Presentation

HSPA2 3306 heat shock protein family A (Hsp70) member 2 HSP70-2|HSP70-3 14 Antigen_Processing_and_Presentation

HSPA4 3308 heat shock protein family A (Hsp70) member 4 APG-2|HEL-S-5a|HS24/P52|HSPH2|RY|hsp70|hsp70RY 5 Antigen_Processing_and_Presentation

HSPA5 3309 heat shock protein family A (Hsp70) member 5 BIP|GRP78|HEL-S-89n|MIF2 9 Antigen_Processing_and_Presentation

HSPA6 3310 heat shock protein family A (Hsp70) member 6 HSP70B' 1 Antigen_Processing_and_Presentation

HSPA8 3312 heat shock protein family A (Hsp70) member 8 HEL-33|HEL-S-72p|HSC54|HSC70|HSC71|HSP71|HSP73|HSPA10|LAP-1|LAP1|NIP71 11 Antigen_Processing_and_Presentation

HSP90AA1 3320 heat shock protein 90 alpha family class A member 1 EL52|HEL-S-65p|HSP86|HSP89A|HSP90A|HSP90N|HSPC1|HSPCA|HSPCAL1|HSPCAL4|HSPN|Hsp103|Hsp89|Hsp90|LAP-2|LAP2 14 Antigen_Processing_and_Presentation

HSP90AB1 3326 heat shock protein 90 alpha family class B member 1 D6S182|HSP84|HSP90B|HSPC2|HSPCB 6 Antigen_Processing_and_Presentation

ICAM1 3383 intercellular adhesion molecule 1 BB2|CD54|P3.58 19 Antigen_Processing_and_Presentation

IFNA1 3439 interferon alpha 1 IFL|IFN|IFN-ALPHA|IFN-alphaD|IFNA13|IFNA@|leIF D 9 Antigen_Processing_and_Presentation

IFNA2 3440 interferon alpha 2 IFN-alpha-2|IFN-alphaA|IFNA|IFNA2B|leIF A 9 Antigen_Processing_and_Presentation

IFNA4 3441 interferon alpha 4 IFN-alpha4a|INFA4 9 Antigen_Processing_and_Presentation

IFNA5 3442 interferon alpha 5 IFN-alpha-5|IFN-alphaG|INA5|INFA5|leIF G 9 Antigen_Processing_and_Presentation

IFNA6 3443 interferon alpha 6 IFN-alphaK 9 Antigen_Processing_and_Presentation

IFNA7 3444 interferon alpha 7 IFN-alphaJ|IFNA-J 9 Antigen_Processing_and_Presentation

IFNA8 3445 interferon alpha 8 IFN-alphaB 9 Antigen_Processing_and_Presentation

IFNA10 3446 interferon alpha 10 IFN-alphaC 9 Antigen_Processing_and_Presentation

IFNA13 3447 interferon alpha 13 - 9 Antigen_Processing_and_Presentation

IFNA14 3448 interferon alpha 14 IFN-alphaH|LEIF2H 9 Antigen_Processing_and_Presentation

IFNA16 3449 interferon alpha 16 IFN-alpha-16|IFN-alphaO 9 Antigen_Processing_and_Presentation

IFNA17 3451 interferon alpha 17 IFN-alphaI|IFNA|INFA|LEIF2C1 9 Antigen_Processing_and_Presentation

IFNA21 3452 interferon alpha 21 IFN-alphaI|LeIF F|leIF-F 9 Antigen_Processing_and_Presentation

IFNG 3458 interferon gamma IFG|IFI 12 Antigen_Processing_and_Presentation

KIR2DL1 3802 killer cell immunoglobulin like receptor, two Ig domains and long cytoplasmic tail 1 CD158A|KIR-K64|KIR221|KIR2DL3|NKAT|NKAT-1|NKAT1|p58.1 19 Antigen_Processing_and_Presentation

KIR2DL2 3803 killer cell immunoglobulin like receptor, two Ig domains and long cytoplasmic tail 2 CD158B1|CD158b|NKAT-6|NKAT6|p58.2 19 Antigen_Processing_and_Presentation

KIR2DL3 3804 killer cell immunoglobulin like receptor, two Ig domains and long cytoplasmic tail 3 CD158B2|CD158b|GL183|KIR-023GB|KIR-K7b|KIR-K7c|KIR2DL|KIR2DS5|KIRCL23|NKAT|NKAT2|NKAT2A|NKAT2B|p58 19 Antigen_Processing_and_Presentation

KIR2DL4 3805 killer cell immunoglobulin like receptor, two Ig domains and long cytoplasmic tail 4 CD158D|G9P|KIR-103AS|KIR-2DL4|KIR103|KIR103AS 19 Antigen_Processing_and_Presentation

KIR2DS1 3806 killer cell immunoglobulin like receptor, two Ig domains and short cytoplasmic tail 1 CD158H|CD158a|p50.1 19 Antigen_Processing_and_Presentation

KIR2DS3 3808 killer cell immunoglobulin like receptor, two Ig domains and short cytoplasmic tail 3 NKAT7 19 Antigen_Processing_and_Presentation

KIR2DS4 3809 killer cell immunoglobulin like receptor, two Ig domains and short cytoplasmic tail 4 CD158I|KIR-2DS4|KIR1D|KIR412|KKA3|NKAT-8|NKAT8 19 Antigen_Processing_and_Presentation

KIR2DS5 3810 killer cell immunoglobulin like receptor, two Ig domains and short cytoplasmic tail 5 CD158G|NKAT9 19 Antigen_Processing_and_Presentation

KIR3DL1 3811 killer cell immunoglobulin like receptor, three Ig domains and long cytoplasmic tail 1 CD158E1|KIR|KIR3DL1/S1|NKAT-3|NKAT3|NKB1|NKB1B 19 Antigen_Processing_and_Presentation

KIR3DL2 3812 killer cell immunoglobulin like receptor, three Ig domains and long cytoplasmic tail 2 3DL2|CD158K|KIR-3DL2|NKAT-4|NKAT4|NKAT4B|p140 19 Antigen_Processing_and_Presentation

KLRC1 3821 killer cell lectin like receptor C1 CD159A|NKG2|NKG2A 12 Antigen_Processing_and_Presentation

KLRC2 3822 killer cell lectin like receptor C2 CD159c|NKG2-C|NKG2C 12 Antigen_Processing_and_Presentation

KLRC3 3823 killer cell lectin like receptor C3 NKG2-E|NKG2E 12 Antigen_Processing_and_Presentation

KLRD1 3824 killer cell lectin like receptor D1 CD94 12 Antigen_Processing_and_Presentation

LTA 4049 lymphotoxin alpha LT|TNFB|TNFSF1|TNLG1E 6 Antigen_Processing_and_Presentation

CIITA 4261 class II major histocompatibility complex transactivator C2TA|CIITAIV|MHC2TA|NLRA 16 Antigen_Processing_and_Presentation

MICA 100507436 MHC class I polypeptide-related sequence A MIC-A|PERB11.1 6 Antigen_Processing_and_Presentation

MICB 4277 MHC class I polypeptide-related sequence B PERB11.2 6 Antigen_Processing_and_Presentation

NFYA 4800 nuclear transcription factor Y subunit alpha CBF-A|CBF-B|HAP2|NF-YA 6 Antigen_Processing_and_Presentation

NFYB 4801 nuclear transcription factor Y subunit beta CBF-A|CBF-B|HAP3|NF-YB 12 Antigen_Processing_and_Presentation

NFYC 4802 nuclear transcription factor Y subunit gamma CBF-C|CBFC|H1TF2A|HAP5|HSM|NF-YC 1 Antigen_Processing_and_Presentation

LGMN 5641 legumain AEP|LGMN1|PRSC1 14 Antigen_Processing_and_Presentation

PSMB8 5696 proteasome 20S subunit beta 8 ALDD|D6S216|D6S216E|JMP|LMP7|NKJO|PRAAS1|PSMB5i|RING10 6 Antigen_Processing_and_Presentation

PSMC1 5700 proteasome 26S subunit, ATPase 1 P26S4|S4|p56 14 Antigen_Processing_and_Presentation

PSMC2 5701 proteasome 26S subunit, ATPase 2 MSS1|Nbla10058|S7 7 Antigen_Processing_and_Presentation

PSMC3 5702 proteasome 26S subunit, ATPase 3 TBP1 11 Antigen_Processing_and_Presentation

PSMC4 5704 proteasome 26S subunit, ATPase 4 MIP224|RPT3|S6|TBP-7|TBP7 19 Antigen_Processing_and_Presentation

PSMC5 5705 proteasome 26S subunit, ATPase 5 S8|SUG-1|SUG1|TBP10|TRIP1|p45|p45/SUG 17 Antigen_Processing_and_Presentation

PSMC6 5706 proteasome 26S subunit, ATPase 6 SUG2|p42 14 Antigen_Processing_and_Presentation

PSMD1 5707 proteasome 26S subunit, non-ATPase 1 P112|Rpn2|S1 2 Antigen_Processing_and_Presentation

PSMD2 5708 proteasome 26S subunit, non-ATPase 2 P97|RPN1|S2|TRAP2 3 Antigen_Processing_and_Presentation

PSMD3 5709 proteasome 26S subunit, non-ATPase 3 P58|RPN3|S3|TSTA2 17 Antigen_Processing_and_Presentation

PSMD4 5710 proteasome 26S subunit, non-ATPase 4 AF|AF-1|ASF|MCB1|Rpn10|S5A|pUB-R5 1 Antigen_Processing_and_Presentation

PSMD5 5711 proteasome 26S subunit, non-ATPase 5 S5B 9 Antigen_Processing_and_Presentation

PSMD7 5713 proteasome 26S subunit, non-ATPase 7 MOV34|P40|Rpn8|S12 16 Antigen_Processing_and_Presentation

PSMD8 5714 proteasome 26S subunit, non-ATPase 8 HEL-S-91n|HIP6|HYPF|Nin1p|Rpn12|S14|p31 19 Antigen_Processing_and_Presentation

PSMD10 5716 proteasome 26S subunit, non-ATPase 10 dJ889N15.2|p28|p28(GANK) X Antigen_Processing_and_Presentation

PSMD11 5717 proteasome 26S subunit, non-ATPase 11 Rpn6|S9|p44.5 17 Antigen_Processing_and_Presentation

PSMD13 5719 proteasome 26S subunit, non-ATPase 13 HSPC027|Rpn9|S11|p40.5 11 Antigen_Processing_and_Presentation

PSME1 5720 proteasome activator subunit 1 HEL-S-129m|IFI5111|PA28A|PA28alpha|REGalpha 14 Antigen_Processing_and_Presentation

PSME1 5720 proteasome activator subunit 1 HEL-S-129m|IFI5111|PA28A|PA28alpha|REGalpha 14 Antigen_Processing_and_Presentation

PSME2 5721 proteasome activator subunit 2 PA28B|PA28beta|REGbeta 14 Antigen_Processing_and_Presentation

PSME2 5721 proteasome activator subunit 2 PA28B|PA28beta|REGbeta 14 Antigen_Processing_and_Presentation

RELB 5971 RELB proto-oncogene, NF-kB subunit I-REL|IMD53|IREL|REL-B 19 Antigen_Processing_and_Presentation

RFX5 5993 regulatory factor X5 - 1 Antigen_Processing_and_Presentation

RFXAP 5994 regulatory factor X associated protein - 13 Antigen_Processing_and_Presentation

SLC10A2 6555 solute carrier family 10 member 2 ASBT|IBAT|ISBT|NTCP2|PBAM 13 Antigen_Processing_and_Presentation

TAP1 6890 transporter 1, ATP binding cassette subfamily B member ABC17|ABCB2|APT1|D6S114E|PSF-1|PSF1|RING4|TAP1*0102N|TAP1N 6 Antigen_Processing_and_Presentation

TAP2 6891 transporter 2, ATP binding cassette subfamily B member ABC18|ABCB3|APT2|D6S217E|PSF-2|PSF2|RING11 6 Antigen_Processing_and_Presentation

TAPBP 6892 TAP binding protein NGS17|TAPA|TPN|TPSN 6 Antigen_Processing_and_Presentation

THBS1 7057 thrombospondin 1 THBS|THBS-1|TSP|TSP-1|TSP1 15 Antigen_Processing_and_Presentation

SEM1 7979 SEM1 26S proteasome complex subunit C7orf76|DSS1|ECD|SHFD1|SHFM1|SHSF1|Shfdg1 7 Antigen_Processing_and_Presentation

KLRC4 8302 killer cell lectin like receptor C4 NKG2-F|NKG2F 12 Antigen_Processing_and_Presentation

AP3B1 8546 adaptor related protein complex 3 subunit beta 1 ADTB3|ADTB3A|HPS|HPS2|PE 5 Antigen_Processing_and_Presentation

RFXANK 8625 regulatory factor X associated ankyrin containing protein ANKRA1|BLS|F14150_1|RFX-B 19 Antigen_Processing_and_Presentation

PSMD6 9861 proteasome 26S subunit, non-ATPase 6 Rpn7|S10|SGA-113M|p42A|p44S10 3 Antigen_Processing_and_Presentation

PSME3 10197 proteasome activator subunit 3 HEL-S-283|Ki|PA28-gamma|PA28G|PA28gamma|REG-GAMMA 17 Antigen_Processing_and_Presentation

PSMD14 10213 proteasome 26S subunit, non-ATPase 14 PAD1|POH1|RPN11 2 Antigen_Processing_and_Presentation

CLEC4M 10332 C-type lectin domain family 4 member M CD209L|CD299|DC-SIGN2|DC-SIGNR|DCSIGNR|HP10347|L-SIGN|LSIGN 19 Antigen_Processing_and_Presentation

IFI30 10437 IFI30 lysosomal thiol reductase GILT|IFI-30|IP-30|IP30 19 Antigen_Processing_and_Presentation

PROCR 10544 protein C receptor CCCA|CCD41|EPCR 20 Antigen_Processing_and_Presentation

ADRM1 11047 adhesion regulating molecule 1 ARM-1|ARM1|GP110 20 Antigen_Processing_and_Presentation

ECPAS 23392 Ecm29 proteasome adaptor and scaffold ECM29|KIAA0368 9 Antigen_Processing_and_Presentation

TRPC4AP 26133 transient receptor potential cation channel subfamily C member 4 associated protein C20orf188|PPP1R158|TRRP4AP|TRUSS 20 Antigen_Processing_and_Presentation

CD209 30835 CD209 molecule CDSIGN|CLEC4L|DC-SIGN|DC-SIGN1 19 Antigen_Processing_and_Presentation

UBXN1 51035 UBX domain protein 1 2B28|SAKS1|UBXD10 11 Antigen_Processing_and_Presentation

ERAP1 51752 endoplasmic reticulum aminopeptidase 1 A-LAP|ALAP|APPILS|ARTS-1|ARTS1|ERAAP|ERAAP1|PILS-AP|PILSAP 5 Antigen_Processing_and_Presentation

TAPBPL 55080 TAP binding protein like TAPBP-R|TAPBPR 12 Antigen_Processing_and_Presentation

KIR2DL5A 57292 killer cell immunoglobulin like receptor, two Ig domains and long cytoplasmic tail 5A CD158F|KIR2DL5|KIR2DL5.1|KIR2DL5.3 19 Antigen_Processing_and_Presentation

ERAP2 64167 endoplasmic reticulum aminopeptidase 2 L-RAP|LRAP 5 Antigen_Processing_and_Presentation

ULBP3 79465 UL16 binding protein 3 N2DL-3|NKG2DL3|RAET1N 6 Antigen_Processing_and_Presentation

ULBP2 80328 UL16 binding protein 2 ALCAN-alpha|N2DL2|NKG2DL2|RAET1H|RAET1L 6 Antigen_Processing_and_Presentation

ULBP1 80329 UL16 binding protein 1 N2DL-1|NKG2DL1|RAET1I 6 Antigen_Processing_and_Presentation

KIR3DL3 115653 killer cell immunoglobulin like receptor, three Ig domains and long cytoplasmic tail 3 CD158Z|KIR3DL7|KIR44|KIRC1 19 Antigen_Processing_and_Presentation

RAET1E 135250 retinoic acid early transcript 1E LETAL|N2DL-4|NKG2DL4|RAET1E2|RL-4|ULBP4|bA350J20.7 6 Antigen_Processing_and_Presentation

RAET1L 154064 retinoic acid early transcript 1L ULBP6 6 Antigen_Processing_and_Presentation

UBR1 197131 ubiquitin protein ligase E3 component n-recognin 1 JBS 15 Antigen_Processing_and_Presentation

RAET1G 353091 retinoic acid early transcript 1G ULBP5 6 Antigen_Processing_and_Presentation

PDIA2 64714 protein disulfide isomerase family A member 2 PDA2|PDI|PDIP|PDIR 16 Antigen_Processing_and_Presentation

HAMP 57817 hepcidin antimicrobial peptide HEPC|HFE2B|LEAP1|PLTR 19 Antimicrobials

PI3 5266 peptidase inhibitor 3 ESI|SKALP|WAP3|WFDC14|cementoin 20 Antimicrobials

CAMP 820 cathelicidin antimicrobial peptide CAP-18|CAP18|CRAMP|FALL-39|FALL39|HSD26|LL37 3 Antimicrobials

DEFB4A 1673 defensin beta 4A BD-2|DEFB-2|DEFB102|DEFB2|DEFB4|HBD-2|SAP1 8 Antimicrobials

PPBP 5473 pro-platelet basic protein B-TG1|Beta-TG|CTAP-III|CTAP3|CTAPIII|CXCL7|LA-PF4|LDGF|MDGF|NAP-2|PBP|SCYB7|TC1|TC2|TGB|TGB1|THBGB|THBGB1 4 Antimicrobials

REG3G 130120 regenerating family member 3 gamma LPPM429|PAP IB|PAP-1B|PAP1B|PAPIB|REG III|REG-III|UNQ429 2 Antimicrobials

CXCL14 9547 C-X-C motif chemokine ligand 14 BMAC|BRAK|KEC|KS1|MIP-2g|MIP2G|NJAC|SCYB14 5 Antimicrobials

CXCL16 58191 C-X-C motif chemokine ligand 16 CXCLG16|SR-PSOX|SRPSOX 17 Antimicrobials

SLPI 6590 secretory leukocyte peptidase inhibitor ALK1|ALP|BLPI|HUSI|HUSI-I|MPI|WAP4|WFDC4 20 Antimicrobials

CXCL8 3576 C-X-C motif chemokine ligand 8 GCP-1|GCP1|IL8|LECT|LUCT|LYNAP|MDNCF|MONAP|NAF|NAP-1|NAP1|SCYB8 4 Antimicrobials

CXCL10 3627 C-X-C motif chemokine ligand 10 C7|IFI10|INP10|IP-10|SCYB10|crg-2|gIP-10|mob-1 4 Antimicrobials

CXCL9 4283 C-X-C motif chemokine ligand 9 CMK|Humig|MIG|SCYB9|crg-10 4 Antimicrobials

CXCL5 6374 C-X-C motif chemokine ligand 5 ENA-78|SCYB5 4 Antimicrobials

CXCL11 6373 C-X-C motif chemokine ligand 11 H174|I-TAC|IP-9|IP9|SCYB11|SCYB9B|b-R1 4 Antimicrobials

CXCL6 6372 C-X-C motif chemokine ligand 6 CKA-3|GCP-2|GCP2|SCYB6 4 Antimicrobials

CXCL1 2919 C-X-C motif chemokine ligand 1 FSP|GRO1|GROa|MGSA|MGSA-a|NAP-3|SCYB1 4 Antimicrobials

CXCL12 6387 C-X-C motif chemokine ligand 12 IRH|PBSF|SCYB12|SDF1|TLSF|TPAR1 10 Antimicrobials

CXCL13 10563 C-X-C motif chemokine ligand 13 ANGIE|ANGIE2|BCA-1|BCA1|BLC|BLR1L|SCYB13 4 Antimicrobials

CXCL2 2920 C-X-C motif chemokine ligand 2 CINC-2a|GRO2|GROb|MGSA-b|MIP-2a|MIP2|MIP2A|SCYB2 4 Antimicrobials

PF4 5196 platelet factor 4 CXCL4|PF-4|SCYB4 4 Antimicrobials

XCL1 6375 X-C motif chemokine ligand 1 ATAC|LPTN|LTN|SCM-1|SCM-1a|SCM1|SCM1A|SCYC1 1 Antimicrobials

CXCL3 2921 C-X-C motif chemokine ligand 3 CINC-2b|GRO3|GROg|MIP-2b|MIP2B|SCYB3 4 Antimicrobials

DEFB103B 55894 defensin beta 103B BD-3|DEFB-3|DEFB103|DEFB3|HBD-3|HBD3|HBP-3|HBP3 8 Antimicrobials

CCL13 6357 C-C motif chemokine ligand 13 CKb10|MCP-4|NCC-1|NCC1|SCYA13|SCYL1 17 Antimicrobials

CCL1 6346 C-C motif chemokine ligand 1 I-309|P500|SCYA1|SISe|TCA3 17 Antimicrobials

DEFB1 1672 defensin beta 1 BD1|DEFB-1|DEFB101|HBD1 8 Antimicrobials

CCL8 6355 C-C motif chemokine ligand 8 HC14|MCP-2|MCP2|SCYA10|SCYA8 17 Antimicrobials

ELANE 1991 elastase, neutrophil expressed ELA2|GE|HLE|HNE|NE|PMN-E|SCN1 19 Antimicrobials

DEFB103A 414325 defensin beta 103A BD-3|DEFB-3|DEFB103|DEFB3|HBD3|HBP-3|HBP3 8 Antimicrobials

DEFA3 1668 defensin alpha 3 DEF3|HNP-3|HNP3|HP-3|HP3 8 Antimicrobials

DEFA1 1667 defensin alpha 1 DEF1|DEFA2|HNP-1|HP-1|HP1|MRS 8 Antimicrobials

TMSB10 9168 thymosin beta 10 MIG12|TB10 2 Antimicrobials

DEFA6 1671 defensin alpha 6 DEF6|HD-6 8 Antimicrobials

DEFA5 1670 defensin alpha 5 DEF5|HD-5 8 Antimicrobials

DEFA4 1669 defensin alpha 4 DEF4|HNP-4|HP-4|HP4 8 Antimicrobials

LCN2 3934 lipocalin 2 24p3|MSFI|NGAL|p25 9 Antimicrobials

LCN1 3933 lipocalin 1 PMFA|TLC|TP|VEGP 9 Antimicrobials

COLEC10 10584 collectin subfamily member 10 3MC3|CL-34|CLL1 8 Antimicrobials

BPI 671 bactericidal permeability increasing protein BPIFD1|rBPI 20 Antimicrobials

S100A9 6280 S100 calcium binding protein A9 60B8AG|CAGB|CFAG|CGLB|L1AG|LIAG|MAC387|MIF|MRP14|NIF|P14 1 Antimicrobials

S100A8 6279 S100 calcium binding protein A8 60B8AG|CAGA|CFAG|CGLA|CP-10|L1Ag|MA387|MIF|MRP8|NIF|P8 1 Antimicrobials

DCD 117159 dermcidin AIDD|DCD-1|DSEP|HCAP|PIF 12 Antimicrobials

LCN6 158062 lipocalin 6 LCN5|UNQ643|hLcn5 9 Antimicrobials

S100A12 6283 S100 calcium binding protein A12 CAAF1|CAGC|CGRP|ENRAGE|MRP-6|MRP6|p6 1 Antimicrobials

HTN3 3347 histatin 3 HIS2|HTN2|HTN5|PB 4 Antimicrobials

LCN8 138307 lipocalin 8 EP17|LCN5 9 Antimicrobials

DEFA1B 728358 defensin alpha 1B HNP-1|HP-1|HP1 8 Antimicrobials

CCR10 2826 C-C motif chemokine receptor 10 GPR2 17 Antimicrobials

CELA1 1990 chymotrypsin like elastase 1 ELA1 12 Antimicrobials

DEFB106A 245909 defensin beta 106A BD-6|DEFB-6|DEFB106 8 Antimicrobials

PENK 5179 proenkephalin PE|PENK-A 8 Antimicrobials

BPIFC 254240 BPI fold containing family C BPIL2 22 Antimicrobials

MMP12 4321 matrix metallopeptidase 12 HME|ME|MME|MMP-12 11 Antimicrobials

BPIFB6 128859 BPI fold containing family B member 6 BPIL3|LPLUNC6 20 Antimicrobials

LEAP2 116842 liver enriched antimicrobial peptide 2 LEAP-2 5 Antimicrobials

SFTPD 6441 surfactant protein D COLEC7|PSP-D|SFTP4|SP-D 10 Antimicrobials

LCN9 392399 lipocalin 9 HEL129 9 Antimicrobials

BPIFB2 80341 BPI fold containing family B member 2 BPIL1|C20orf184|LPLUNC2|RYSR|dJ726C3.2 20 Antimicrobials

PTGDS 5730 prostaglandin D2 synthase L-PGDS|LPGDS|PDS|PGD2|PGDS|PGDS2 9 Antimicrobials

TMSB4X 7114 thymosin beta 4 X-linked FX|PTMB4|TB4X|TMSB4 X Antimicrobials

PGLYRP1 8993 peptidoglycan recognition protein 1 PGLYRP|PGRP|PGRP-S|PGRPS|TAG7|TNFSF3L 19 Antimicrobials

ZC3HAV1 56829 zinc finger CCCH-type containing, antiviral 1 ARTD13|FLB6421|PARP13|ZAP|ZC3H2|ZC3HDC2 7 Antimicrobials

TMSB15A 11013 thymosin beta 15a TMSB15|TMSB15B|TMSL8|TMSNB|Tb15|TbNB X Antimicrobials

S100B 6285 S100 calcium binding protein B NEF|S100|S100-B|S100beta 21 Antimicrobials

S100A13 6284 S100 calcium binding protein A13 - 1 Antimicrobials

S100A6 6277 S100 calcium binding protein A6 2A9|5B10|CABP|CACY|PRA|S10A6 1 Antimicrobials

DEFB119 245932 defensin beta 119 DEFB-19|DEFB-20|DEFB120|DEFB20|ESC42-RELA|ESC42-RELB 20 Antimicrobials

DEFB107A 245910 defensin beta 107A BD-7|DEFB-7|DEFB107 8 Antimicrobials

DEFB105A 245908 defensin beta 105A BD-5|DEFB-5|DEFB105 8 Antimicrobials

SERPIND1 3053 serpin family D member 1 D22S673|HC2|HCF2|HCII|HLS2|LS2|THPH10 22 Antimicrobials

DEFB129 140881 defensin beta 129 C20orf87|DEFB-29|DEFB29|bA530N10.3|hBD-29 20 Antimicrobials

DEFB127 140850 defensin beta 127 C20orf73|DEF-27|DEFB-27|DEFB27|bA530N10.2|hBD-27 20 Antimicrobials

S100P 6286 S100 calcium binding protein P MIG9 4 Antimicrobials

S100A7 6278 S100 calcium binding protein A7 PSOR1|S100A7c 1 Antimicrobials

DEFB104A 140596 defensin beta 104A BD-4|DEFB-4|DEFB104|DEFB4|hBD-4 8 Antimicrobials

DEFB126 81623 defensin beta 126 C20orf8|DEFB-26|DEFB26|HBD26|bA530N10.1|hBD-26 20 Antimicrobials

DEFB106B 503841 defensin beta 106B BD-6|DEFB-6 8 Antimicrobials

DEFB104B 503618 defensin beta 104B BD-4|DEFB-4|hBD-4 8 Antimicrobials

DEFB107B 503614 defensin beta 107B HsT21816 8 Antimicrobials

PGLYRP3 114771 peptidoglycan recognition protein 3 PGLYRPIalpha|PGRP-Ialpha|PGRPIA 1 Antimicrobials

PGLYRP2 114770 peptidoglycan recognition protein 2 HMFT0141|PGLYRPL|PGRP-L|PGRPL|TAGL-like|tagL|tagL-alpha|tagl-beta 19 Antimicrobials

S100A10 6281 S100 calcium binding protein A10 42C|ANX2L|ANX2LG|CAL1L|CLP11|Ca[1]|GP11|P11|p10 1 Antimicrobials

S100A2 6273 S100 calcium binding protein A2 CAN19|S100L 1 Antimicrobials

DEFB125 245938 defensin beta 125 DEFB-25 20 Antimicrobials

DEFB123 245936 defensin beta 123 DEFB-23|DEFB23|ESC42-RELD 20 Antimicrobials

DEFB105B 504180 defensin beta 105B BD-5|DEFB-5 8 Antimicrobials

DEFB132 400830 defensin beta 132 BD-32|DEFB-32|DEFB32|HEL-75|KFLL827|UNQ827 20 Antimicrobials

BPIFB3 359710 BPI fold containing family B member 3 C20orf185|LPLUNC3|RYA3 20 Antimicrobials

LCN12 286256 lipocalin 12 - 9 Antimicrobials

PGLYRP4 57115 peptidoglycan recognition protein 4 PGLYRPIbeta|PGRP-Ibeta|PGRPIB|SBBI67 1 Antimicrobials

S100A11 6282 S100 calcium binding protein A11 HEL-S-43|MLN70|S100C 1 Antimicrobials

S100A5 6276 S100 calcium binding protein A5 S100D 1 Antimicrobials

S100A3 6274 S100 calcium binding protein A3 S100E 1 Antimicrobials

S100A1 6271 S100 calcium binding protein A1 S100|S100-alpha|S100A 1 Antimicrobials

DEFB128 245939 defensin beta 128 DEFB-28|DEFB28|hBD-28 20 Antimicrobials

DEFB108B 245911 defensin beta 108B DEFB-8|hBD-8 11 Antimicrobials

HTN1 3346 histatin 1 HIS1 4 Antimicrobials

LMBR1L 55716 limb development membrane protein 1 like LIMR 12 Antimicrobials

S100A7A 338324 S100 calcium binding protein A7A NICE-2|NICE2|S100A15|S100A7L1|S100A7f 1 Antimicrobials

DEFB118 117285 defensin beta 118 C20orf63|DEFB-18|ESC42|ESP13.6 20 Antimicrobials

COLEC12 81035 collectin subfamily member 12 CLP1|NSR2|SCARA4|SRCL 18 Antimicrobials

TMSB4Y 9087 thymosin beta 4 Y-linked TB4Y Y Antimicrobials

DEFB131A 644414 defensin beta 131A DEFB-31|DEFB131 4 Antimicrobials

DEFB134 613211 defensin beta 134 - 8 Antimicrobials

DEFB130A 245940 defensin beta 130A DEFB-30|DEFB130|DEFB130L|DEFB30 8 Antimicrobials

DEFB124 245937 defensin beta 124 DEFB-24 20 Antimicrobials

DEFB121 245934 defensin beta 121 DEFB21|ESC42RELC 20 Antimicrobials

DEFB116 245930 defensin beta 116 DEFB-16 20 Antimicrobials

DEFB115 245929 defensin beta 115 DEFB-15 20 Antimicrobials

DEFB114 245928 defensin beta 114 DEFB-14|DEFB14 6 Antimicrobials

DEFB113 245927 defensin beta 113 DEFB-13 6 Antimicrobials

DEFB112 245915 defensin beta 112 DEFB-12 6 Antimicrobials

DEFB110 245913 defensin beta 110 DEFB-10|DEFB-11|DEFB111 6 Antimicrobials

TMSB15B 286527 thymosin beta 15B TMSB15A|TMSL8|TMSNB|Tbeta15b X Antimicrobials

DEFB133 403339 defensin beta 133 - 6 Antimicrobials

S100Z 170591 S100 calcium binding protein Z Gm625|S100-zeta 5 Antimicrobials

MAVS 57506 mitochondrial antiviral signaling protein CARDIF|IPS-1|IPS1|VISA 20 Antimicrobials

TMSB4XP8 7117 TMSB4X pseudogene 8 TMSL3 4 Antimicrobials

S100A14 57402 S100 calcium binding protein A14 BCMP84|S100A15 1 Antimicrobials

LCN10 414332 lipocalin 10 - 9 Antimicrobials

S100A16 140576 S100 calcium binding protein A16 AAG13|DT1P1A7|S100F 1 Antimicrobials

DEFB136 613210 defensin beta 136 DEFB137 8 Antimicrobials

DEFB135 613209 defensin beta 135 DEFB136 8 Antimicrobials

DEFB117 245931 defensin beta 117 (pseudogene) DEFB-17 20 Antimicrobials

DEFB110 245913 defensin beta 110 DEFB-10|DEFB-11|DEFB111 6 Antimicrobials

ZC3HAV1L 92092 zinc finger CCCH-type containing, antiviral 1 like C7orf39 7 Antimicrobials

S100A7L2 645922 S100 calcium binding protein A7 like 2 S100a7b 1 Antimicrobials

MBL3P 50639 mannose-binding lectin family member 3, pseudogene COLEC2|MBL 10 Antimicrobials

DEFB4B 100289462 defensin beta 4B DEFB4P 8 Antimicrobials

BPIFB4 149954 BPI fold containing family B member 4 C20orf186|LPLUNC4|RY2G5|dJ726C3.5 20 Antimicrobials

IFNAR1 3454 interferon alpha and beta receptor subunit 1 AVP|IFN-alpha-REC|IFNAR|IFNBR|IFRC 21 Antimicrobials

AZU1 566 azurocidin 1 AZAMP|AZU|CAP37|HBP|HUMAZUR|NAZC|hHBP 19 Antimicrobials

DEFB131B 100129216 defensin beta 131B - 11 Antimicrobials

DEFA1A3 613253 defensin alpha 1 and alpha 3, variable copy number locus DEFA1|DEFA3|DEFT1P 8 Antimicrobials

LCN1P1 286310 lipocalin 1 pseudogene 1 LCN1L1|bA430N14.2 9 Antimicrobials

S100G 795 S100 calcium binding protein G CABP|CABP1|CABP9K|CALB3 X Antimicrobials

DEFA7P 724067 defensin alpha 7, pseudogene DEFA7 8 Antimicrobials

DEFB130B 100133267 defensin beta 130B - 8 Antimicrobials

DEFB108F 100133128 defensin beta 108F (pseudogene) DEFB108P5 4 Antimicrobials

DEFB131C 100128174 defensin beta 131C (pseudogene) - 8 Antimicrobials

TCHHL1 126637 trichohyalin like 1 S100A17|THHL1 1 Antimicrobials

TINAGL1 64129 tubulointerstitial nephritis antigen like 1 ARG1|LCN7|LIECG3|TINAGRP 1 Antimicrobials

IFNGR1 3459 interferon gamma receptor 1 CD119|IFNGR|IMD27A|IMD27B 6 Antimicrobials

SLC22A17 51310 solute carrier family 22 member 17 24p3R|BOCT|BOIT|NGALR|NGALR2|NGALR3|hBOIT 14 Antimicrobials

WFIKKN1 117166 WAP, follistatin/kazal, immunoglobulin, kunitz and netrin domain containing 1 C16orf12|RJD2|WFDC20A|WFIKKN 16 Antimicrobials

WFDC2 10406 WAP four-disulfide core domain 2 EDDM4|HE4|WAP5|dJ461P17.6 20 Antimicrobials

IL6 3569 interleukin 6 BSF-2|BSF2|CDF|HGF|HSF|IFN-beta-2|IFNB2|IL-6 7 Antimicrobials

UMODL1 89766 uromodulin like 1 - 21 Antimicrobials

TGFB1 7040 transforming growth factor beta 1 CED|DPD1|IBDIMDE|LAP|TGF-beta1|TGFB|TGFbeta 19 Antimicrobials

PF4V1 5197 platelet factor 4 variant 1 CXCL4L1|CXCL4V1|PF4-ALT|PF4A|SCYB4V1 4 Antimicrobials

MMP9 4318 matrix metallopeptidase 9 CLG4B|GELB|MANDP2|MMP-9 20 Antimicrobials

ANOS1 3730 anosmin 1 ADMLX|HH1|HHA|KAL|KAL1|KALIG-1|KMS|WFDC19 X Antimicrobials

TLR4 7099 toll like receptor 4 ARMD10|CD284|TLR-4|TOLL 9 Antimicrobials

IFNG 3458 interferon gamma IFG|IFI 12 Antimicrobials

SPAG11B 10407 sperm associated antigen 11B EDDM2B|EP2|EP2C|EP2D|HE2|HE2C|SPAG11|SPAG11A 8 Antimicrobials

A2M 2 alpha-2-macroglobulin A2MD|CPAMD5|FWP007|S863-7 12 Antimicrobials

CTSL 1514 cathepsin L CATL|CTSL1|MEP 9 Antimicrobials

NFKB1 4790 nuclear factor kappa B subunit 1 CVID12|EBP-1|KBF1|NF-kB|NF-kB1|NF-kappa-B1|NF-kappaB|NF-kappabeta|NFKB-p105|NFKB-p50|NFkappaB 4 Antimicrobials

APOBEC3G 60489 apolipoprotein B mRNA editing enzyme catalytic subunit 3G A3G|ARCD|ARP-9|ARP9|CEM-15|CEM15|MDS019|bK150C2.7|dJ494G10.1 22 Antimicrobials

FABP6 2172 fatty acid binding protein 6 I-15P|I-BABP|I-BALB|I-BAP|ILBP|ILBP3|ILLBP 5 Antimicrobials

NOD2 64127 nucleotide binding oligomerization domain containing 2 ACUG|BLAU|BLAUS|CARD15|CD|CLR16.3|IBD1|NLRC2|NOD2B|PSORAS1|YAOS 16 Antimicrobials

MBL2 4153 mannose binding lectin 2 COLEC1|HSMBPC|MBL|MBL2D|MBP|MBP-C|MBP1|MBPD 10 Antimicrobials

SFTPA1 653509 surfactant protein A1 COLEC4|PSAP|PSP-A|PSPA|SFTP1|SFTPA1B|SP-A|SP-A1|SP-A1 beta|SP-A1 delta|SP-A1 epsilon|SP-A1 gamma|SPA|SPA1 10 Antimicrobials

RBP1 5947 retinol binding protein 1 CRABP-I|CRBP|CRBP1|CRBPI|RBPC 3 Antimicrobials

TLR2 7097 toll like receptor 2 CD282|TIL4 4 Antimicrobials

SLC40A1 30061 solute carrier family 40 member 1 FPN1|HFE4|IREG1|MST079|MSTP079|MTP1|SLC11A3 2 Antimicrobials

PLAU 5328 plasminogen activator, urokinase ATF|BDPLT5|QPD|UPA|URK|u-PA 10 Antimicrobials

IL1B 3553 interleukin 1 beta IL-1|IL1-BETA|IL1F2|IL1beta 2 Antimicrobials

PAEP 5047 progestagen associated endometrial protein GD|GdA|GdF|GdS|PAEG|PEP|PP14|ZIF-1 9 Antimicrobials

HJV 148738 hemojuvelin BMP co-receptor HFE2|HFE2A|JH|RGMC 1 Antimicrobials

MUC5AC 4586 mucin 5AC, oligomeric mucus/gel-forming MUC5|TBM|leB|mucin 11 Antimicrobials

CTSS 1520 cathepsin S - 1 Antimicrobials

OBP2A 29991 odorant binding protein 2A LCN13|OBP|OBP2C|OBPIIa|hOBPIIa 9 Antimicrobials

PLTP 5360 phospholipid transfer protein BPIFE|HDLCQ9 20 Antimicrobials

MX1 4599 MX dynamin like GTPase 1 IFI-78K|IFI78|MX|MxA|lncMX1-215 21 Antimicrobials

DDX58 23586 DExD/H-box helicase 58 RIG-I|RIG1|RIGI|RLR-1|SGMRT2 9 Antimicrobials

IFNL1 282618 interferon lambda 1 IL-29|IL29 19 Antimicrobials

IRF3 3661 interferon regulatory factor 3 IIAE7 19 Antimicrobials

SFTPA2 729238 surfactant protein A2 COLEC5|PSAP|PSP-A|PSPA|SFTP1|SFTPA2B|SP-2A|SP-A|SPA2|SPAII 10 Antimicrobials

LPA 4018 lipoprotein(a) AK38|APOA|LP 6 Antimicrobials

LBP 3929 lipopolysaccharide binding protein BPIFD2 20 Antimicrobials

RBP4 5950 retinol binding protein 4 MCOPCB10|RDCCAS 10 Antimicrobials

SFTPA1 653509 surfactant protein A1 COLEC4|PSAP|PSP-A|PSPA|SFTP1|SFTPA1B|SP-A|SP-A1|SP-A1 beta|SP-A1 delta|SP-A1 epsilon|SP-A1 gamma|SPA|SPA1 10 Antimicrobials

NOX4 50507 NADPH oxidase 4 KOX|KOX-1|RENOX 11 Antimicrobials

LTF 4057 lactotransferrin GIG12|HEL110|HLF2|LF 3 Antimicrobials

IFNB1 3456 interferon beta 1 IFB|IFF|IFN-beta|IFNB 9 Antimicrobials

RBP5 83758 retinol binding protein 5 CRBP-III|CRBP3|CRBPIII|HRBPiso 12 Antimicrobials

FABP7 2173 fatty acid binding protein 7 B-FABP|BLBP|FABPB|MRG 6 Antimicrobials

FABP5 2171 fatty acid binding protein 5 E-FABP|EFABP|KFABP|PA-FABP|PAFABP 8 Antimicrobials

FABP3 2170 fatty acid binding protein 3 FABP11|H-FABP|M-FABP|MDGI|O-FABP 1 Antimicrobials

FABP2 2169 fatty acid binding protein 2 FABPI|I-FABP 4 Antimicrobials

FABP4 2167 fatty acid binding protein 4 A-FABP|AFABP|ALBP|HEL-S-104|aP2 8 Antimicrobials

R3HDML 140902 R3H domain containing like dJ881L22.3 20 Antimicrobials

BPIFA3 128861 BPI fold containing family A member 3 C20orf71|SPLUNC3 20 Antimicrobials

BPIFB1 92747 BPI fold containing family B member 1 C20orf114|LPLUNC1 20 Antimicrobials

OASL 8638 2'-5'-oligoadenylate synthetase like OASL1|OASLd|TRIP-14|TRIP14|p59 OASL|p59-OASL|p59OASL 12 Antimicrobials

CRABP2 1382 cellular retinoic acid binding protein 2 CRABP-II|RBP6 1 Antimicrobials

CRABP1 1381 cellular retinoic acid binding protein 1 CRABP|CRABP-I|CRABPI|RBP5 15 Antimicrobials

RBP7 116362 retinol binding protein 7 CRABP4|CRBP4|CRBPIV 1 Antimicrobials

DUOX1 53905 dual oxidase 1 LNOX1|NOXEF1|THOX1 15 Antimicrobials

OBP2B 29989 odorant binding protein 2B LCN14|OBPIIb 9 Antimicrobials

RBP2 5948 retinol binding protein 2 CRABP-II|CRBP2|CRBPII|RBPC2 3 Antimicrobials

LCN15 389812 lipocalin 15 PRO6093|UNQ2541 9 Antimicrobials

CETP 1071 cholesteryl ester transfer protein BPIFF|HDLCQ10 16 Antimicrobials

FABP12 646486 fatty acid binding protein 12 - 8 Antimicrobials

FABP9 646480 fatty acid binding protein 9 PERF|PERF15|T-FABP|TLBP 8 Antimicrobials

BPIFA1 51297 BPI fold containing family A member 1 LUNX|NASG|PLUNC|SPLUNC1|SPURT|bA49G10.5 20 Antimicrobials

LCNL1 401562 lipocalin like 1 - 9 Antimicrobials

C8G 733 complement C8 gamma chain C8C 9 Antimicrobials

SPAG11A 653423 sperm associated antigen 11A EDDM2A|HE2 8 Antimicrobials

PI15 51050 peptidase inhibitor 15 CRISP8|P24TI|P25TI 8 Antimicrobials

NOX1 27035 NADPH oxidase 1 GP91-2|MOX1|NOH-1|NOH1 X Antimicrobials

PMP2 5375 peripheral myelin protein 2 CMT1G|FABP8|M-FABP|MP2|P2 8 Antimicrobials

APOD 347 apolipoprotein D - 3 Antimicrobials

ORM2 5005 orosomucoid 2 AGP-B|AGP-B'|AGP2 9 Antimicrobials

ORM1 5004 orosomucoid 1 AGP-A|AGP1|HEL-S-153w|ORM 9 Antimicrobials

TNF 7124 tumor necrosis factor DIF|TNF-alpha|TNFA|TNFSF2|TNLG1F 6 Antimicrobials

CTSG 1511 cathepsin G CATG|CG 14 Antimicrobials

PRTN3 5657 proteinase 3 ACPA|AGP7|C-ANCA|CANCA|MBN|MBT|NP-4|NP4|P29|PR-3|PR3 19 Antimicrobials

MAPK1 5594 mitogen-activated protein kinase 1 ERK|ERK-2|ERK2|ERT1|MAPK2|P42MAPK|PRKM1|PRKM2|p38|p40|p41|p41mapk|p42-MAPK 22 Antimicrobials

PML 5371 PML nuclear body scaffold MYL|PP8675|RNF71|TRIM19 15 Antimicrobials

AEN 64782 apoptosis enhancing nuclease ISG20L1|pp12744 15 Antimicrobials

CYBB 1536 cytochrome b-245 beta chain AMCBX2|CGD|GP91-1|GP91-PHOX|GP91PHOX|IMD34|NOX2|p91-PHOX X Antimicrobials

BPIFA2 140683 BPI fold containing family A member 2 C20orf70|PSP|SPLUNC2|bA49G10.1 20 Antimicrobials

ISG20 3669 interferon stimulated exonuclease gene 20 CD25|HEM45 15 Antimicrobials

BCL3 602 BCL3 transcription coactivator BCL4|D19S37 19 Antimicrobials

ISG20L2 81875 interferon stimulated exonuclease gene 20 like 2 HSD38 1 Antimicrobials

NOX5 79400 NADPH oxidase 5 - 15 Antimicrobials

NOX3 50508 NADPH oxidase 3 GP91-3|MOX-2 6 Antimicrobials

DUOX2 50506 dual oxidase 2 LNOX2|NOXEF2|P138-TOX|TDH6|THOX2 15 Antimicrobials

TLR3 7098 toll like receptor 3 CD283|IIAE2 4 Antimicrobials

TFRC 7037 transferrin receptor CD71|IMD46|T9|TFR|TFR1|TR|TRFR|p90 3 Antimicrobials

IFIH1 64135 interferon induced with helicase C domain 1 AGS7|Hlcd|IDDM19|MDA-5|MDA5|RLR-2|SGMRT1 2 Antimicrobials

LRP1 4035 LDL receptor related protein 1 A2MR|APOER|APR|CD91|IGFBP-3R|IGFBP3R|IGFBP3R1|KPA|LRP|LRP1A|TGFBR5 12 Antimicrobials

TRIM5 85363 tripartite motif containing 5 RNF88|TRIM5alpha 11 Antimicrobials

IDO1 3620 indoleamine 2,3-dioxygenase 1 IDO|IDO-1|INDO 8 Antimicrobials

GDF15 9518 growth differentiation factor 15 GDF-15|MIC-1|MIC1|NAG-1|PDF|PLAB|PTGFB 19 Antimicrobials

NEDD4 4734 NEDD4 E3 ubiquitin protein ligase NEDD4-1|RPF1 15 Antimicrobials

ADIPOQ 9370 adiponectin, C1Q and collagen domain containing ACDC|ACRP30|ADIPQTL1|ADPN|APM-1|APM1|GBP28 3 Antimicrobials

STAT3 6774 signal transducer and activator of transcription 3 ADMIO|ADMIO1|APRF|HIES 17 Antimicrobials

STAT1 6772 signal transducer and activator of transcription 1 CANDF7|IMD31A|IMD31B|IMD31C|ISGF-3|STAT91 2 Antimicrobials

IFNL2 282616 interferon lambda 2 IL-28A|IL28A 19 Antimicrobials

SOCS3 9021 suppressor of cytokine signaling 3 ATOD4|CIS3|Cish3|SOCS-3|SSI-3|SSI3 17 Antimicrobials

SEMG1 6406 semenogelin 1 CT103|SEMG|SGI|dJ172H20.2 20 Antimicrobials

TNFSF10 8743 TNF superfamily member 10 APO2L|Apo-2L|CD253|TL2|TNLG6A|TRAIL 3 Antimicrobials

CCL20 6364 C-C motif chemokine ligand 20 CKb4|Exodus|LARC|MIP-3-alpha|MIP-3a|MIP3A|SCYA20|ST38 2 Antimicrobials

SOCS1 8651 suppressor of cytokine signaling 1 CIS1|CISH1|JAB|SOCS-1|SSI-1|SSI1|TIP-3|TIP3 16 Antimicrobials

RNASEL 6041 ribonuclease L PRCA1|RNS4 1 Antimicrobials

IRF1 3659 interferon regulatory factor 1 IRF-1|MAR 5 Antimicrobials

IL15 3600 interleukin 15 IL-15 4 Antimicrobials

APOBEC3F 200316 apolipoprotein B mRNA editing enzyme catalytic subunit 3F A3F|ARP8|BK150C2.4.MRNA|KA6 22 Antimicrobials

PLAAT4 5920 phospholipase A and acyltransferase 4 HRASLS4|HRSL4|PLA1/2-3|PLAAT-4|RARRES3|RIG1|TIG3 11 Antimicrobials

CHIT1 1118 chitinase 1 CHI3|CHIT|CHITD 1 Antimicrobials

IFNA1 3439 interferon alpha 1 IFL|IFN|IFN-ALPHA|IFN-alphaD|IFNA13|IFNA@|leIF D 9 Antimicrobials

CD40 958 CD40 molecule Bp50|CDW40|TNFRSF5|p50 20 Antimicrobials

TLR7 51284 toll like receptor 7 TLR7-like X Antimicrobials

PPIA 5478 peptidylprolyl isomerase A CYPA|CYPH|HEL-S-69p 7 Antimicrobials

HFE 3077 homeostatic iron regulator HFE1|HH|HLA-H|MVCD7|TFQTL2 6 Antimicrobials

ZYX 7791 zyxin ESP-2|HED-2 7 Antimicrobials

NLRX1 79671 NLR family member X1 CLR11.3|DLNB26|NOD26|NOD5|NOD9 11 Antimicrobials

PGC 5225 progastricsin PEPC|PGII 6 Antimicrobials

VEGFA 7422 vascular endothelial growth factor A MVCD1|VEGF|VPF 6 Antimicrobials

IKBKE 9641 inhibitor of nuclear factor kappa B kinase subunit epsilon IKK-E|IKK-i|IKKE|IKKI 1 Antimicrobials

ISG15 9636 ISG15 ubiquitin like modifier G1P2|IFI15|IMD38|IP17|UCRP|hUCRP 1 Antimicrobials

DHX58 79132 DExH-box helicase 58 D11LGP2|D11lgp2e|LGP2|RLR-3 17 Antimicrobials

TNFAIP3 7128 TNF alpha induced protein 3 A20|AISBL|OTUD7C|TNFA1P2 6 Antimicrobials

TFR2 7036 transferrin receptor 2 HFE3|TFRC2 7 Antimicrobials

FCN2 2220 ficolin 2 EBP-37|FCNL|P35|ficolin-2 9 Antimicrobials

MUC4 4585 mucin 4, cell surface associated ASGP|HSA276359|MUC-4 3 Antimicrobials

F2R 2149 coagulation factor II thrombin receptor CF2R|HTR|PAR-1|PAR1|TR 5 Antimicrobials

ELN 2006 elastin ADCL1|SVAS|WBS|WS 7 Antimicrobials

IL27 246778 interleukin 27 IL-27|IL-27A|IL27A|IL27p28|IL30|p28 16 Antimicrobials

MAPT 4137 microtubule associated protein tau DDPAC|FTDP-17|MAPTL|MSTD|MTBT1|MTBT2|PPND|PPP1R103|TAU 17 Antimicrobials

LYZ 4069 lysozyme LYZF1|LZM 12 Antimicrobials

CCL5 6352 C-C motif chemokine ligand 5 D17S136E|RANTES|SCYA5|SIS-delta|SISd|TCP228|eoCP 17 Antimicrobials

LEP 3952 leptin LEPD|OB|OBS 7 Antimicrobials

CYLD 1540 CYLD lysine 63 deubiquitinase BRSS|CDMT|CYLD1|CYLDI|EAC|MFT|MFT1|SBS|TEM|USPL2 16 Antimicrobials

KLKB1 3818 kallikrein B1 KLK3|PKK|PKKD|PPK 4 Antimicrobials

CST4 1472 cystatin S - 20 Antimicrobials

CSRP1 1465 cysteine and glycine rich protein 1 CRP|CRP1|CSRP|CYRP|D1S181E|HEL-141|HEL-S-286 1 Antimicrobials

MAPK14 1432 mitogen-activated protein kinase 14 CSBP|CSBP1|CSBP2|CSPB1|EXIP|Mxi2|PRKM14|PRKM15|RK|SAPK2A|p38|p38ALPHA 6 Antimicrobials

JUN 3725 Jun proto-oncogene, AP-1 transcription factor subunit AP-1|AP1|c-Jun|cJUN|p39 1 Antimicrobials

ITGAV 3685 integrin subunit alpha V CD51|MSK8|VNRA|VTNR 2 Antimicrobials

IRF5 3663 interferon regulatory factor 5 SLEB10 7 Antimicrobials

CCR6 1235 C-C motif chemokine receptor 6 BN-1|C-C CKR-6|CC-CKR-6|CCR-6|CD196|CKR-L3|CKRL3|CMKBR6|DCR2|DRY6|GPR29|GPRCY4|STRL22 6 Antimicrobials

IL12B 3593 interleukin 12B CLMF|CLMF2|IL-12B|IMD28|IMD29|NKSF|NKSF2 5 Antimicrobials

TLR8 51311 toll like receptor 8 CD288 X Antimicrobials

GNLY 10578 granulysin D2S69E|LAG-2|LAG2|NKG5|TLA519 2 Antimicrobials

CD81 975 CD81 molecule CVID6|S5.7|TAPA1|TSPAN28 11 Antimicrobials

EIF2AK2 5610 eukaryotic translation initiation factor 2 alpha kinase 2 EIF2AK1|LEUDEN|PKR|PPP1R83|PRKR 2 Antimicrobials

APOM 55937 apolipoprotein M G3a|HSPC336|NG20|apo-M 6 Antimicrobials

CACYBP 27101 calcyclin binding protein GIG5|PNAS-107|S100A6BP|SIP 1 Antimicrobials

NOD1 10392 nucleotide binding oligomerization domain containing 1 CARD4|CLR7.1|NLRC1 7 Antimicrobials

MAPK8 5599 mitogen-activated protein kinase 8 JNK|JNK-46|JNK1|JNK1A2|JNK21B1/2|PRKM8|SAPK1|SAPK1c 10 Antimicrobials

MAPK3 5595 mitogen-activated protein kinase 3 ERK-1|ERK1|ERT2|HS44KDAP|HUMKER1A|P44ERK1|P44MAPK|PRKM3|p44-ERK1|p44-MAPK 16 Antimicrobials

BST2 684 bone marrow stromal cell antigen 2 CD317|TETHERIN 19 Antimicrobials

BPHL 670 biphenyl hydrolase like BPH-RP|MCNAA|VACVASE 6 Antimicrobials

PLA2G2A 5320 phospholipase A2 group IIA MOM1|PLA2|PLA2B|PLA2L|PLA2S|PLAS1|sPLA2 1 Antimicrobials

GRN 2896 granulin precursor CLN11|GEP|GP88|PCDGF|PEPI|PGRN 17 Antimicrobials

NEWENTRY 192343 - - - Antimicrobials

PDGFRA 5156 platelet derived growth factor receptor alpha CD140A|PDGFR-2|PDGFR2 4 Antimicrobials

GNAI1 2770 G protein subunit alpha i1 Gi 7 Antimicrobials

WNT5A 7474 Wnt family member 5A hWNT5A 3 Antimicrobials

FURIN 5045 furin, paired basic amino acid cleaving enzyme FUR|PACE|PCSK3|SPC1 15 Antimicrobials

ADAR 103 adenosine deaminase RNA specific ADAR1|AGS6|DRADA|DSH|DSRAD|G1P1|IFI-4|IFI4|K88DSRBP|P136 1 Antimicrobials

TYK2 7297 tyrosine kinase 2 IMD35|JTK1 19 Antimicrobials

NOS2 4843 nitric oxide synthase 2 HEP-NOS|INOS|NOS|NOS2A 17 Antimicrobials

TRAF3 7187 TNF receptor associated factor 3 CAP-1|CAP1|CD40bp|CRAF1|IIAE5|LAP1|RNF118 14 Antimicrobials

TPT1 7178 tumor protein, translationally-controlled 1 HRF|TCTP|p02|p23 13 Antimicrobials

TPM2 7169 tropomyosin 2 AMCD1|DA1|DA2B|DA2B4|HEL-S-273|NEM4|TMSB 9 Antimicrobials

NEO1 4756 neogenin 1 IGDCC2|NGN|NTN1R2 15 Antimicrobials

AHNAK 79026 AHNAK nucleoprotein AHNAKRS|PM227 11 Antimicrobials

TLR1 7096 toll like receptor 1 CD281|TIL|TIL. LPRS5|rsc786 4 Antimicrobials

TK2 7084 thymidine kinase 2 MTDPS2|MTTK|PEOB3|SCA31 16 Antimicrobials

PRDX2 7001 peroxiredoxin 2 HEL-S-2a|NKEF-B|NKEFB|PRP|PRX2|PRXII|PTX1|TDPX1|TPX1|TSA 19 Antimicrobials

MX2 4600 MX dynamin like GTPase 2 MXB 21 Antimicrobials

FGF2 2247 fibroblast growth factor 2 BFGF|FGF-2|FGFB|HBGF-2 4 Antimicrobials

FGA 2243 fibrinogen alpha chain Fib2 4 Antimicrobials

TCF7L2 6934 transcription factor 7 like 2 TCF-4|TCF4 10 Antimicrobials

F2RL1 2150 F2R like trypsin receptor 1 GPR11|PAR2 5 Antimicrobials

TKFC 26007 triokinase and FMN cyclase DAK|NET45|TKFCD 11 Antimicrobials

MSR1 4481 macrophage scavenger receptor 1 CD204|SCARA1|SR-A|SR-AI|SR-AII|SR-AIII|SRA|phSR1|phSR2 8 Antimicrobials

NFKBIZ 64332 NFKB inhibitor zeta IKBZ|INAP|MAIL 3 Antimicrobials

LMBR1 64327 limb development membrane protein 1 ACHP|C7orf2|DIF14|LSS|PPD2|THYP|TPT|ZRS 7 Antimicrobials

EPPIN 57119 epididymal peptidase inhibitor CT71|CT72|SPINLW1|WAP7|WFDC7|dJ461P17.2 20 Antimicrobials

SRC 6714 SRC proto-oncogene, non-receptor tyrosine kinase ASV|SRC1|THC6|c-SRC|p60-Src 20 Antimicrobials

MPO 4353 myeloperoxidase - 17 Antimicrobials

ELAVL1 1994 ELAV like RNA binding protein 1 ELAV1|HUR|Hua|MelG 19 Antimicrobials

ROBO3 64221 roundabout guidance receptor 3 HGPPS|HGPPS1|HGPS|RBIG1|RIG1 11 Antimicrobials

SP1 6667 Sp1 transcription factor - 12 Antimicrobials

SOD1 6647 superoxide dismutase 1 ALS|ALS1|HEL-S-44|IPOA|SOD|STAHP|hSod1|homodimer 21 Antimicrobials

PDF 64146 peptide deformylase, mitochondrial - 16 Antimicrobials

DLL4 54567 delta like canonical Notch ligand 4 AOS6|delta4|hdelta2 15 Antimicrobials

ECD 11319 ecdysoneless cell cycle regulator GCR2|HSGT1|SGT1 10 Antimicrobials

SLC11A1 6556 solute carrier family 11 member 1 LSH|NRAMP|NRAMP1 2 Antimicrobials

DMBT1 1755 deleted in malignant brain tumors 1 GP340|SAG|SALSA|muclin 10 Antimicrobials

STING1 340061 stimulator of interferon response cGAMP interactor 1 ERIS|MITA|MPYS|NET23|SAVI|STING|STING-beta|TMEM173|hMITA|hSTING 5 Antimicrobials

SKIV2L 6499 Ski2 like RNA helicase 170A|DDX13|HLP|SKI2|SKI2W|SKIV2|SKIV2L1|THES2 6 Antimicrobials

SEMG2 6407 semenogelin 2 SGII 20 Antimicrobials

LTA 4049 lymphotoxin alpha LT|TNFB|TNFSF1|TNLG1E 6 Antimicrobials

DES 1674 desmin CDCD3|CSM1|CSM2|LGMD1D|LGMD1E|LGMD2R 2 Antimicrobials

DCK 1633 deoxycytidine kinase - 4 Antimicrobials

DAXX 1616 death domain associated protein BING2|DAP6|EAP1|SMIM40 6 Antimicrobials

TNFRSF10A 8797 TNF receptor superfamily member 10a APO2|CD261|DR4|TRAILR-1|TRAILR1 8 Antimicrobials

TNFRSF10B 8795 TNF receptor superfamily member 10b CD262|DR5|KILLER|KILLER/DR5|TRAIL-R2|TRAILR2|TRICK2|TRICK2A|TRICK2B|TRICKB|ZTNFR9 8 Antimicrobials

EED 8726 embryonic ectoderm development COGIS|HEED|WAIT1 11 Antimicrobials

CCL4 6351 C-C motif chemokine ligand 4 ACT2|AT744.1|G-26|HC21|LAG-1|LAG1|MIP-1-beta|MIP1B|MIP1B1|SCYA2|SCYA4 17 Antimicrobials

LIMS1 3987 LIM zinc finger domain containing 1 PINCH|PINCH-1|PINCH1 2 Antimicrobials

LALBA 3906 lactalbumin alpha LYZG 12 Antimicrobials

APOBEC3H 164668 apolipoprotein B mRNA editing enzyme catalytic subunit 3H A3H|ARP-10|ARP10 22 Antimicrobials

TMPRSS6 164656 transmembrane serine protease 6 IRIDA|MT2 22 Antimicrobials

SPINK5 11005 serine peptidase inhibitor Kazal type 5 LEKTI|LETKI|NETS|NS|VAKTI 5 Antimicrobials

MARCO 8685 macrophage receptor with collagenous structure SCARA2|SR-A6 2 Antimicrobials

BECN1 8678 beclin 1 ATG6|VPS30|beclin1 17 Antimicrobials

TNFSF11 8600 TNF superfamily member 11 CD254|ODF|OPGL|OPTB2|RANKL|TNLG6B|TRANCE|hRANKL2|sOdf 13 Antimicrobials

KNG1 3827 kininogen 1 BDK|BK|HMWK|KNG 3 Antimicrobials

CSK 1445 C-terminal Src kinase - 15 Antimicrobials

KLRK1 22914 killer cell lectin like receptor K1 CD314|D12S2489E|KLR|NKG2-D|NKG2D 12 Antimicrobials

KCNH2 3757 potassium voltage-gated channel subfamily H member 2 ERG-1|ERG1|H-ERG|HERG|HERG1|Kv11.1|LQT2|SQT1 7 Antimicrobials

JUND 3727 JunD proto-oncogene, AP-1 transcription factor subunit AP-1 19 Antimicrobials

JAK1 3716 Janus kinase 1 JAK1A|JAK1B|JTK3 1 Antimicrobials

CREB1 1385 cAMP responsive element binding protein 1 CREB|CREB-1 2 Antimicrobials

CLDN4 1364 claudin 4 CPE-R|CPER|CPETR|CPETR1|WBSCR8|hCPE-R 7 Antimicrobials

CCL28 56477 C-C motif chemokine ligand 28 CCK1|MEC|SCYA28 5 Antimicrobials

RNASE3 6037 ribonuclease A family member 3 ECP|RAF1|RNS3 14 Antimicrobials

RN7SL1 6029 RNA component of signal recognition particle 7SL1 7L1a|7SL|RN7SL|RNSRP1 14 Antimicrobials

IRF7 3665 interferon regulatory factor 7 IMD39|IRF-7|IRF-7H|IRF7A|IRF7B|IRF7C|IRF7H 11 Antimicrobials

IREB2 3658 iron responsive element binding protein 2 ACO3|IRE-BP 2|IRE-BP2|IRP2|IRP2AD|NDCAMA 15 Antimicrobials

ILK 3611 integrin linked kinase HEL-S-28|ILK-1|ILK-2|P59|p59ILK 11 Antimicrobials

IL18 3606 interleukin 18 IGIF|IL-18|IL-1g|IL1F4 11 Antimicrobials

IL17A 3605 interleukin 17A CTLA-8|CTLA8|IL-17|IL-17A|IL17 6 Antimicrobials

LTB4R 1241 leukotriene B4 receptor BLT1|BLTR|CMKRL1|GPR16|LTB4R1|LTBR1|P2RY7|P2Y7 14 Antimicrobials

APOBEC3A 200315 apolipoprotein B mRNA editing enzyme catalytic subunit 3A A3A|ARP3|PHRBN|bK150C2.1 22 Antimicrobials

MASP2 10747 mannan binding lectin serine peptidase 2 MAP19|MASP-2|MASP1P1|sMAP 1 Antimicrobials

TRIM27 5987 tripartite motif containing 27 RFP|RNF76 6 Antimicrobials

RELA 5970 RELA proto-oncogene, NF-kB subunit CMCU|NFKB3|p65 11 Antimicrobials

IL7R 3575 interleukin 7 receptor CD127|CDW127|IL-7R-alpha|IL7RA|ILRA 5 Antimicrobials

IL1A 3552 interleukin 1 alpha IL-1 alpha|IL-1A|IL1|IL1-ALPHA|IL1F1 2 Antimicrobials

PTX3 5806 pentraxin 3 TNFAIP5|TSG-14 3 Antimicrobials

IFNAR2 3455 interferon alpha and beta receptor subunit 2 IFN-R|IFN-alpha-REC|IFNABR|IFNARB|IMD45 21 Antimicrobials

IFN1@ 3438 - IFNA 9 Antimicrobials

SYTL1 84958 synaptotagmin like 1 JFC1|SLP1 1 Antimicrobials

APOBEC3C 27350 apolipoprotein B mRNA editing enzyme catalytic subunit 3C A3C|APOBEC1L|ARDC2|ARDC4|ARP5|PBI|bK150C2.3 22 Antimicrobials

DDX17 10521 DEAD-box helicase 17 P72|RH70 22 Antimicrobials

PTGS2 5743 prostaglandin-endoperoxide synthase 2 COX-2|COX2|GRIPGHS|PGG/HS|PGHS-2|PHS-2|hCox-2 1 Antimicrobials

HTR1A 3350 5-hydroxytryptamine receptor 1A 5-HT-1A|5-HT1A|5HT1a|ADRB2RL1|ADRBRL1|G-21|PFMCD 5 Antimicrobials

SEPTIN7 989 septin 7 CDC10|CDC3|NBLA02942|SEPT7|SEPT7A 7 Antimicrobials

CD40LG 959 CD40 ligand CD154|CD40L|HIGM1|IGM|IMD3|T-BAM|TNFSF5|TRAP|gp39|hCD40L X Antimicrobials

CD14 929 CD14 molecule - 5 Antimicrobials

CD8A 925 CD8a molecule CD8|Leu2|p32 2 Antimicrobials

CD4 920 CD4 molecule CD4mut 12 Antimicrobials

MASP1 5648 mannan binding lectin serine peptidase 1 3MC1|CRARF|CRARF1|MAP1|MASP|MASP3|MAp44|PRSS5|RaRF 3 Antimicrobials

PROC 5624 protein C, inactivator of coagulation factors Va and VIIIa APC|PC|PROC1|THPH3|THPH4 2 Antimicrobials

MAP2K2 5605 mitogen-activated protein kinase kinase 2 CFC4|MAPKK2|MEK2|MKK2|PRKMK2 19 Antimicrobials

MAP2K1 5604 mitogen-activated protein kinase kinase 1 CFC3|MAPKK1|MEK1|MKK1|PRKMK1 15 Antimicrobials

HRG 3273 histidine rich glycoprotein HPRG|HRGP|THPH11 3 Antimicrobials

NDRG1 10397 N-myc downstream regulated 1 CAP43|CMT4D|DRG-1|DRG1|GC4|HMSNL|NDR1|NMSL|PROXY1|RIT42|RTP|TARG1|TDD5 8 Antimicrobials

IRF9 10379 interferon regulatory factor 9 IRF-9|ISGF3|ISGF3G|p48 14 Antimicrobials

TRIM22 10346 tripartite motif containing 22 GPSTAF50|RNF94|STAF50 11 Antimicrobials

LANCL1 10314 LanC like 1 GPR69A|p40 2 Antimicrobials

PPP4C 5531 protein phosphatase 4 catalytic subunit PP-X|PP4|PP4C|PPH3|PPP4|PPX 16 Antimicrobials

HMOX1 3162 heme oxygenase 1 HMOX1D|HO-1|HSP32|bK286B10 22 Antimicrobials

HMGB1 3146 high mobility group box 1 HMG-1|HMG1|HMG3|SBP-1 13 Antimicrobials

HLA-B 3106 major histocompatibility complex, class I, B AS|B-4901|HLAB 6 Antimicrobials

RNASE7 84659 ribonuclease A family member 7 RAE1 14 Antimicrobials

ABCC4 10257 ATP binding cassette subfamily C member 4 MOAT-B|MOATB|MRP4 13 Antimicrobials

HGF 3082 hepatocyte growth factor DFNB39|F-TCF|HGFB|HPTA|SF 7 Antimicrobials

HDAC1 3065 histone deacetylase 1 GON-10|HD1|KDAC1|RPD3|RPD3L1 1 Antimicrobials

IFNLR1 163702 interferon lambda receptor 1 CRF2/12|IFNLR|IL-28R1|IL28RA|LICR2 1 Antimicrobials

PLSCR1 5359 phospholipid scramblase 1 MMTRA1B 3 Antimicrobials

B2M 567 beta-2-microglobulin IMD43 15 Antimicrobials

BACH2 60468 BTB domain and CNC homolog 2 BTBD25|IMD60 6 Antimicrobials

TANK 10010 TRAF family member associated NFKB activator I-TRAF|ITRAF|TRAF2 2 Antimicrobials

PIK3CG 5294 phosphatidylinositol-4,5-bisphosphate 3-kinase catalytic subunit gamma PI3CG|PI3K|PI3Kgamma|PIK3|p110gamma|p120-PI3K 7 Antimicrobials

ARRB1 408 arrestin beta 1 ARB1|ARR1 11 Antimicrobials

RSAD2 91543 radical S-adenosyl methionine domain containing 2 2510004L01Rik|cig33|cig5|vig1 2 Antimicrobials

STAB2 55576 stabilin 2 FEEL2|FELE-2|FELL2|FEX2|HARE|SCARH1 12 Antimicrobials

TBK1 29110 TANK binding kinase 1 FTDALS4|IIAE8|NAK|T2K 12 Antimicrobials

PDYN 5173 prodynorphin ADCA|PENKB|SCA23 20 Antimicrobials

PDGFRB 5159 platelet derived growth factor receptor beta CD140B|IBGC4|IMF1|JTK12|KOGS|PDGFR|PDGFR-1|PDGFR1|PENTT 5 Antimicrobials

PDCD1 5133 programmed cell death 1 CD279|PD-1|PD1|SLEB2|hPD-1|hPD-l|hSLE1 2 Antimicrobials

PCSK2 5126 proprotein convertase subtilisin/kexin type 2 NEC 2|NEC-2|NEC2|PC2|SPC2 20 Antimicrobials

PCSK1 5122 proprotein convertase subtilisin/kexin type 1 BMIQ12|NEC1|PC1|PC3|SPC3 5 Antimicrobials

ARG2 384 arginase 2 - 14 Antimicrobials

AQP9 366 aquaporin 9 AQP-9|HsT17287|SSC1|T17287 15 Antimicrobials

FASLG 356 Fas ligand ALPS1B|APT1LG1|APTL|CD178|CD95-L|CD95L|FASL|TNFSF6|TNLG1A 1 Antimicrobials

APOH 350 apolipoprotein H B2G1|B2GP1|BG 17 Antimicrobials

BIRC5 332 baculoviral IAP repeat containing 5 API4|EPR-1 17 Antimicrobials

ANXA6 309 annexin A6 ANX6|CBP68|CPB-II|p68|p70 5 Antimicrobials

IL22 50616 interleukin 22 IL-21|IL-22|IL-D110|IL-TIF|ILTIF|TIFIL-23|TIFa|zcyto18 12 Antimicrobials

VTN 7448 vitronectin V75|VN|VNT 17 Antimicrobials

VIM 7431 vimentin - 10 Antimicrobials

VCAM1 7412 vascular cell adhesion molecule 1 CD106|INCAM-100 1 Antimicrobials

PRDX1 5052 peroxiredoxin 1 MSP23|NKEF-A|NKEFA|PAG|PAGA|PAGB|PRX1|PRXI|TDPX2 1 Antimicrobials

GFAP 2670 glial fibrillary acidic protein ALXDRD 17 Antimicrobials

GBP2 2634 guanylate binding protein 2 - 1 Antimicrobials

ALB 213 albumin HSA|PRO0883|PRO0903|PRO1341 4 Antimicrobials

SLC29A3 55315 solute carrier family 29 member 3 ENT3|HCLAP|HJCD|PHID 10 Antimicrobials

OAS1 4938 2'-5'-oligoadenylate synthetase 1 E18/E16|IFI-4|OIAS|OIASI 12 Antimicrobials

AGER 177 advanced glycosylation end-product specific receptor RAGE|SCARJ1 6 Antimicrobials

UNC93B1 81622 unc-93 homolog B1, TLR signaling regulator IIAE1|UNC93|UNC93B|Unc-93B1 11 Antimicrobials

TNFSF4 7292 TNF superfamily member 4 CD134L|CD252|GP34|OX-40L|OX4OL|TNLG2B|TXGP1 1 Antimicrobials

NOS1 4842 nitric oxide synthase 1 IHPS1|N-NOS|NC-NOS|NOS|bNOS|nNOS 12 Antimicrobials

ACTG1 71 actin gamma 1 ACT|ACTG|DFNA20|DFNA26|HEL-176 17 Antimicrobials

ACTA1 58 actin alpha 1, skeletal muscle ACTA|ASMA|CFTD|CFTD1|CFTDM|MPFD|NEM1|NEM2|NEM3|SHPM 1 Antimicrobials

ACO1 48 aconitase 1 ACONS|HEL60|IREB1|IREBP|IREBP1|IRP1 9 Antimicrobials

SERPINA3 12 serpin family A member 3 AACT|ACT|GIG24|GIG25 14 Antimicrobials

CXCR1 3577 C-X-C motif chemokine receptor 1 C-C|C-C-CKR-1|CD128|CD181|CDw128a|CKR-1|CMKAR1|IL8R1|IL8RA|IL8RBA 2 Antimicrobials

CCL15 6359 C-C motif chemokine ligand 15 HCC-2|HMRP-2B|LKN-1|LKN1|MIP-1 delta|MIP-1D|MIP-5|MRP-2B|NCC-3|NCC3|SCYA15|SCYL3|SY15 17 Antimicrobials

CCL14 6358 C-C motif chemokine ligand 14 CC-1|CC-3|CKB1|HCC-1|HCC-1(1-74)|HCC-1/HCC-3|HCC-3|MCIF|NCC-2|NCC2|SCYA14|SCYL2|SY14 17 Antimicrobials

CCL4 6351 C-C motif chemokine ligand 4 ACT2|AT744.1|G-26|HC21|LAG-1|LAG1|MIP-1-beta|MIP1B|MIP1B1|SCYA2|SCYA4 17 Antimicrobials

CCL16 6360 C-C motif chemokine ligand 16 CKb12|HCC-4|ILINCK|LCC-1|LEC|LMC|Mtn-1|NCC-4|NCC4|SCYA16|SCYL4 17 Antimicrobials

CCL19 6363 C-C motif chemokine ligand 19 CKb11|ELC|MIP-3b|MIP3B|SCYA19 9 Antimicrobials

CCL13 6357 C-C motif chemokine ligand 13 CKb10|MCP-4|NCC-1|NCC1|SCYA13|SCYL1 17 Antimicrobials

CCL18 6362 C-C motif chemokine ligand 18 AMAC-1|AMAC1|CKb7|DC-CK1|DCCK1|MIP-4|PARC|SCYA18 17 Antimicrobials

CCL17 6361 C-C motif chemokine ligand 17 A-152E5.3|ABCD-2|SCYA17|TARC 16 Antimicrobials

CCL26 10344 C-C motif chemokine ligand 26 IMAC|MIP-4a|MIP-4alpha|SCYA26|TSC-1 7 Antimicrobials

CCL22 6367 C-C motif chemokine ligand 22 A-152E5.1|ABCD-1|DC/B-CK|MDC|SCYA22|STCP-1 16 Antimicrobials

CCR3 1232 C-C motif chemokine receptor 3 C C CKR3|CC-CKR-3|CD193|CKR 3|CKR3|CMKBR3 3 Antimicrobials

CCL28 56477 C-C motif chemokine ligand 28 CCK1|MEC|SCYA28 5 Antimicrobials

CCL4L1 388372 C-C motif chemokine ligand 4 like 1 AT744.2|CCL4L|LAG-1|LAG1|MIP-1-beta|SCYA4L|SCYA4L1|SCYA4L2 17 Antimicrobials

ACKR2 1238 atypical chemokine receptor 2 CCBP2|CCR10|CCR9|CMKBR9|D6|hD6 3 Antimicrobials

CCR7 1236 C-C motif chemokine receptor 7 BLR2|CC-CKR-7|CCR-7|CD197|CDw197|CMKBR7|EBI1 17 Antimicrobials

CCL27 10850 C-C motif chemokine ligand 27 ALP|CTACK|CTAK|ESKINE|ILC|PESKY|SCYA27 9 Antimicrobials

CCR8 1237 C-C motif chemokine receptor 8 CC-CKR-8|CCR-8|CDw198|CKRL1|CMKBR8|CMKBRL2|CY6|GPRCY6|TER1 3 Antimicrobials

ACKR4 51554 atypical chemokine receptor 4 CC-CKR-11|CCBP2|CCR-11|CCR10|CCR11|CCRL1|CCX CKR|CCX-CKR|CKR-11|PPR1|VSHK1 3 Antimicrobials

CCR10 2826 C-C motif chemokine receptor 10 GPR2 17 Antimicrobials

CCL2 6347 C-C motif chemokine ligand 2 GDCF-2|HC11|HSMCR30|MCAF|MCP-1|MCP1|SCYA2|SMC-CF 17 Antimicrobials

CCL21 6366 C-C motif chemokine ligand 21 6Ckine|CKb9|ECL|SCYA21|SLC|TCA4 9 Antimicrobials

CCL7 6354 C-C motif chemokine ligand 7 FIC|MARC|MCP-3|MCP3|NC28|SCYA6|SCYA7 17 Antimicrobials

CCL5 6352 C-C motif chemokine ligand 5 D17S136E|RANTES|SCYA5|SIS-delta|SISd|TCP228|eoCP 17 Antimicrobials

CCL3 6348 C-C motif chemokine ligand 3 G0S19-1|LD78ALPHA|MIP-1-alpha|MIP1A|SCYA3 17 Antimicrobials

CCL20 6364 C-C motif chemokine ligand 20 CKb4|Exodus|LARC|MIP-3-alpha|MIP-3a|MIP3A|SCYA20|ST38 2 Antimicrobials

CCL11 6356 C-C motif chemokine ligand 11 SCYA11 17 Antimicrobials

CCR5 1234 C-C motif chemokine receptor 5 CC-CKR-5|CCCKR5|CCR-5|CD195|CKR-5|CKR5|CMKBR5|IDDM22 3 Antimicrobials

CCL23 6368 C-C motif chemokine ligand 23 CK-BETA-8|CKb8|Ckb-8|Ckb-8-1|MIP-3|MIP3|MPIF-1|SCYA23|hmrp-2a 17 Antimicrobials

CCL25 6370 C-C motif chemokine ligand 25 Ckb15|SCYA25|TECK 19 Antimicrobials

CCL1 6346 C-C motif chemokine ligand 1 I-309|P500|SCYA1|SISe|TCA3 17 Antimicrobials

CCL3L3 414062 C-C motif chemokine ligand 3 like 3 464.2|D17S1718|G0S19-2|LD78|LD78BETA|SCYA3L|SCYA3L1 17 Antimicrobials

CCL4L2 9560 C-C motif chemokine ligand 4 like 2 AT744.2|CCL4L|SCYA4L|SCYQ4L2 17 Antimicrobials

CXCL12 6387 C-X-C motif chemokine ligand 12 IRH|PBSF|SCYB12|SDF1|TLSF|TPAR1 10 Antimicrobials

XCL1 6375 X-C motif chemokine ligand 1 ATAC|LPTN|LTN|SCM-1|SCM-1a|SCM1|SCM1A|SCYC1 1 Antimicrobials

CCL8 6355 C-C motif chemokine ligand 8 HC14|MCP-2|MCP2|SCYA10|SCYA8 17 Antimicrobials

CCL3L1 6349 C-C motif chemokine ligand 3 like 1 464.2|D17S1718|G0S19-2|LD78|LD78-beta(1-70)|LD78BETA|MIP1AP|SCYA3L|SCYA3L1 17 Antimicrobials

CCR1 1230 C-C motif chemokine receptor 1 CD191|CKR-1|CKR1|CMKBR1|HM145|MIP1aR|SCYAR1 3 Antimicrobials

CCL24 6369 C-C motif chemokine ligand 24 Ckb-6|MPIF-2|MPIF2|SCYA24 7 Antimicrobials

XCL2 6846 X-C motif chemokine ligand 2 SCM-1b|SCM1B|SCYC2 1 Antimicrobials

CXCL1 2919 C-X-C motif chemokine ligand 1 FSP|GRO1|GROa|MGSA|MGSA-a|NAP-3|SCYB1 4 Antimicrobials

CXCL10 3627 C-X-C motif chemokine ligand 10 C7|IFI10|INP10|IP-10|SCYB10|crg-2|gIP-10|mob-1 4 Antimicrobials

CXCR4 7852 C-X-C motif chemokine receptor 4 CD184|D2S201E|FB22|HM89|HSY3RR|LAP-3|LAP3|LCR1|LESTR|NPY3R|NPYR|NPYRL|NPYY3R|WHIM|WHIMS 2 Antimicrobials

CXCL2 2920 C-X-C motif chemokine ligand 2 CINC-2a|GRO2|GROb|MGSA-b|MIP-2a|MIP2|MIP2A|SCYB2 4 Antimicrobials

CXCR6 10663 C-X-C motif chemokine receptor 6 BONZO|CD186|STRL33|TYMSTR 3 Antimicrobials

CCR4 1233 C-C motif chemokine receptor 4 CC-CKR-4|CD194|CKR4|CMKBR4|ChemR13|HGCN:14099|K5-5 3 Antimicrobials

CXCL11 6373 C-X-C motif chemokine ligand 11 H174|I-TAC|IP-9|IP9|SCYB11|SCYB9B|b-R1 4 Antimicrobials

TAFA5 25817 TAFA chemokine like family member 5 FAM19A5|QLLK5208|TAFA-5|UNQ5208 22 Antimicrobials

TAFA3 284467 TAFA chemokine like family member 3 FAM19A3|TAFA-3 1 Antimicrobials

TAFA4 151647 TAFA chemokine like family member 4 FAM19A4|TAFA-4 3 Antimicrobials

TAFA1 407738 TAFA chemokine like family member 1 FAM19A1|TAFA-1 3 Antimicrobials

TAFA2 338811 TAFA chemokine like family member 2 FAM19A2|TAFA-2 12 Antimicrobials

CCL15-CCL14 348249 CCL15-CCL14 readthrough (NMD candidate) CCL15|HCC-2|LKN-1|MIP-5|MIP5|Mrp-2b|NCC-3|NCC3|SCYA15 17 Antimicrobials

IL6 3569 interleukin 6 BSF-2|BSF2|CDF|HGF|HSF|IFN-beta-2|IFNB2|IL-6 7 Antimicrobials

TNF 7124 tumor necrosis factor DIF|TNF-alpha|TNFA|TNFSF2|TNLG1F 6 Antimicrobials

IL1B 3553 interleukin 1 beta IL-1|IL1-BETA|IL1F2|IL1beta 2 Antimicrobials

IL18 3606 interleukin 18 IGIF|IL-18|IL-1g|IL1F4 11 Antimicrobials

PTK2B 2185 protein tyrosine kinase 2 beta CADTK|CAKB|FADK2|FAK2|PKB|PTK|PYK2|RAFTK 8 Antimicrobials

VEGFA 7422 vascular endothelial growth factor A MVCD1|VEGF|VPF 6 Antimicrobials

IL4 3565 interleukin 4 BCGF-1|BCGF1|BSF-1|BSF1|IL-4 5 Antimicrobials

CDH1 999 cadherin 1 Arc-1|BCDS1|CD324|CDHE|ECAD|LCAM|UVO 16 Antimicrobials

CD40 958 CD40 molecule Bp50|CDW40|TNFRSF5|p50 20 Antimicrobials

DEFB103B 55894 defensin beta 103B BD-3|DEFB-3|DEFB103|DEFB3|HBD-3|HBD3|HBP-3|HBP3 8 Antimicrobials

F2RL1 2150 F2R like trypsin receptor 1 GPR11|PAR2 5 Antimicrobials

MMP9 4318 matrix metallopeptidase 9 CLG4B|GELB|MANDP2|MMP-9 20 Antimicrobials

LTBP1 4052 latent transforming growth factor beta binding protein 1 - 2 Antimicrobials

DEFB4A 1673 defensin beta 4A BD-2|DEFB-2|DEFB102|DEFB2|DEFB4|HBD-2|SAP1 8 Antimicrobials

TNFSF10 8743 TNF superfamily member 10 APO2L|Apo-2L|CD253|TL2|TNLG6A|TRAIL 3 Antimicrobials

IL13 3596 interleukin 13 IL-13|P600 5 Antimicrobials

IL10 3586 interleukin 10 CSIF|GVHDS|IL-10|IL10A|TGIF 1 Antimicrobials

IL2 3558 interleukin 2 IL-2|TCGF|lymphokine 4 Antimicrobials

PPARG 5468 peroxisome proliferator activated receptor gamma CIMT1|GLM1|NR1C3|PPARG1|PPARG2|PPARG5|PPARgamma 3 Antimicrobials

FGR 2268 FGR proto-oncogene, Src family tyrosine kinase SRC2|c-fgr|c-src2|p55-Fgr|p55c-fgr|p58-Fgr|p58c-fgr 1 Antimicrobials

MIF 4282 macrophage migration inhibitory factor GIF|GLIF|MMIF 22 Antimicrobials

CRP 1401 C-reactive protein PTX1 1 Antimicrobials

JAK2 3717 Janus kinase 2 JTK10|THCYT3 9 Antimicrobials

IL1A 3552 interleukin 1 alpha IL-1 alpha|IL-1A|IL1|IL1-ALPHA|IL1F1 2 Antimicrobials

PTK2 5747 protein tyrosine kinase 2 FADK|FAK|FAK1|FRNK|PPP1R71|p125FAK|pp125FAK 8 Antimicrobials

PTGDR 5729 prostaglandin D2 receptor AS1|ASRT1|DP|DP1|PTGDR1 14 Antimicrobials

CD86 942 CD86 molecule B7-2|B7.2|B70|CD28LG2|LAB72 3 Antimicrobials

HCK 3055 HCK proto-oncogene, Src family tyrosine kinase JTK9|p59Hck|p61Hck 20 Antimicrobials

ARRB1 408 arrestin beta 1 ARB1|ARR1 11 Antimicrobials

GNAI1 2770 G protein subunit alpha i1 Gi 7 Antimicrobials

VDR 7421 vitamin D receptor NR1I1|PPP1R163 12 Antimicrobials

OLR1 4973 oxidized low density lipoprotein receptor 1 CLEC8A|LOX1|LOXIN|SCARE1|SLOX1 12 Antimicrobials

GRK2 156 G protein-coupled receptor kinase 2 ADRBK1|BARK1|BETA-ARK1 11 Antimicrobials

TXK 7294 TXK tyrosine kinase BTKL|PSCTK5|PTK4|RLK|TKL 4 Antimicrobials

RNASE2 6036 ribonuclease A family member 2 EDN|RAF3|RNS2 14 Antimicrobials

CD79A 973 CD79a molecule IGA|MB-1 19 BCRSignalingPathway

CD79B 974 CD79b molecule AGM6|B29|IGB 17 BCRSignalingPathway

LYN 4067 LYN proto-oncogene, Src family tyrosine kinase JTK8|p53Lyn|p56Lyn 8 BCRSignalingPathway

SYK 6850 spleen associated tyrosine kinase p72-Syk 9 BCRSignalingPathway

BTK 695 Bruton tyrosine kinase AGMX1|AT|ATK|BPK|IGHD3|IMD1|PSCTK1|XLA X BCRSignalingPathway

BLNK 29760 B cell linker AGM4|BASH|BLNK-S|LY57|SLP-65|SLP65|bca 10 BCRSignalingPathway

VAV3 10451 vav guanine nucleotide exchange factor 3 - 1 BCRSignalingPathway

VAV1 7409 vav guanine nucleotide exchange factor 1 VAV 19 BCRSignalingPathway

VAV2 7410 vav guanine nucleotide exchange factor 2 VAV-2 9 BCRSignalingPathway

RAC1 5879 Rac family small GTPase 1 MIG5|MRD48|Rac-1|TC-25|p21-Rac1 7 BCRSignalingPathway

RAC2 5880 Rac family small GTPase 2 EN-7|Gx|HSPC022|p21-Rac2 22 BCRSignalingPathway

RAC3 5881 Rac family small GTPase 3 - 17 BCRSignalingPathway

PPP3CA 5530 protein phosphatase 3 catalytic subunit alpha ACCIID|CALN|CALNA|CALNA1|CCN1|CNA1|IECEE|IECEE1|PPP2B 4 BCRSignalingPathway

PPP3CB 5532 protein phosphatase 3 catalytic subunit beta CALNA2|CALNB|CNA2|PP2Bbeta 10 BCRSignalingPathway

PPP3CC 5533 protein phosphatase 3 catalytic subunit gamma CALNA3|CNA3|PP2Bgamma 8 BCRSignalingPathway

CHP1 11261 calcineurin like EF-hand protein 1 CHP|SLC9A1BP|SPAX9|Sid470p|p22|p24 15 BCRSignalingPathway

PPP3R1 5534 protein phosphatase 3 regulatory subunit B, alpha CALNB1|CNB|CNB1 2 BCRSignalingPathway

PPP3R2 5535 protein phosphatase 3 regulatory subunit B, beta PPP3RL 9 BCRSignalingPathway

CHP2 63928 calcineurin like EF-hand protein 2 - 16 BCRSignalingPathway

NFAT5 10725 nuclear factor of activated T cells 5 NF-AT5|NFATL1|NFATZ|OREBP|TONEBP 16 BCRSignalingPathway

NFATC1 4772 nuclear factor of activated T cells 1 NF-ATC|NF-ATc1.2|NFAT2|NFATc 18 BCRSignalingPathway

NFATC2 4773 nuclear factor of activated T cells 2 NFAT1|NFATP 20 BCRSignalingPathway

NFATC3 4775 nuclear factor of activated T cells 3 NF-AT4c|NFAT4|NFATX 16 BCRSignalingPathway

NFATC4 4776 nuclear factor of activated T cells 4 NF-AT3|NF-ATC4|NFAT3 14 BCRSignalingPathway

HRAS 3265 HRas proto-oncogene, GTPase C-BAS/HAS|C-H-RAS|C-HA-RAS1|CTLO|H-RASIDX|HAMSV|HRAS1|RASH1|p21ras 11 BCRSignalingPathway

KRAS 3845 KRAS proto-oncogene, GTPase 'C-K-RAS|C-K-RAS|CFC2|K-RAS2A|K-RAS2B|K-RAS4A|K-RAS4B|K-Ras|K-Ras 2|KI-RAS|KRAS1|KRAS2|NS|NS3|OES|RALD|RASK2|c-Ki-ras|c-Ki-ras2 12 BCRSignalingPathway

NRAS 4893 NRAS proto-oncogene, GTPase ALPS4|CMNS|N-ras|NCMS|NRAS1|NS6 1 BCRSignalingPathway

FOS 2353 Fos proto-oncogene, AP-1 transcription factor subunit AP-1|C-FOS|p55 14 BCRSignalingPathway

JUN 3725 Jun proto-oncogene, AP-1 transcription factor subunit AP-1|AP1|c-Jun|cJUN|p39 1 BCRSignalingPathway

CARD11 84433 caspase recruitment domain family member 11 BENTA|BIMP3|CARMA1|IMD11|IMD11A|PPBL 7 BCRSignalingPathway

BCL10 8915 BCL10 immune signaling adaptor CARMEN|CIPER|CLAP|IMD37|c-E10|mE10 1 BCRSignalingPathway

MALT1 10892 MALT1 paracaspase IMD12|MLT|MLT1|PCASP1 18 BCRSignalingPathway

CHUK 1147 component of inhibitor of nuclear factor kappa B kinase complex IKBKA|IKK-alpha|IKK1|IKKA|NFKBIKA|TCF16 10 BCRSignalingPathway

IKBKB 3551 inhibitor of nuclear factor kappa B kinase subunit beta IKK-beta|IKK2|IKKB|IMD15|IMD15A|IMD15B|NFKBIKB 8 BCRSignalingPathway

IKBKG 8517 inhibitor of nuclear factor kappa B kinase regulatory subunit gamma AMCBX1|EDAID1|FIP-3|FIP3|Fip3p|IKK-gamma|IKKAP1|IKKG|IMD33|IP|IP1|IP2|IPD2|NEMO|ZC2HC9 X BCRSignalingPathway

NFKB1 4790 nuclear factor kappa B subunit 1 CVID12|EBP-1|KBF1|NF-kB|NF-kB1|NF-kappa-B1|NF-kappaB|NF-kappabeta|NFKB-p105|NFKB-p50|NFkappaB 4 BCRSignalingPathway

RELA 5970 RELA proto-oncogene, NF-kB subunit CMCU|NFKB3|p65 11 BCRSignalingPathway

NFKBIA 4792 NFKB inhibitor alpha EDAID2|IKBA|MAD-3|NFKBI 14 BCRSignalingPathway

NFKBIB 4793 NFKB inhibitor beta IKBB|TRIP9 19 BCRSignalingPathway

NFKBIE 4794 NFKB inhibitor epsilon IKBE 6 BCRSignalingPathway

CD81 975 CD81 molecule CVID6|S5.7|TAPA1|TSPAN28 11 BCRSignalingPathway

CD19 930 CD19 molecule B4|CVID3 16 BCRSignalingPathway

CR2 1380 complement C3d receptor 2 C3DR|CD21|CR|CVID7|SLEB9 1 BCRSignalingPathway

PIK3R5 23533 phosphoinositide-3-kinase regulatory subunit 5 F730038I15Rik|FOAP-2|P101-PI3K|p101 17 BCRSignalingPathway

PIK3R1 5295 phosphoinositide-3-kinase regulatory subunit 1 AGM7|GRB1|IMD36|p85|p85-ALPHA 5 BCRSignalingPathway

PIK3R2 5296 phosphoinositide-3-kinase regulatory subunit 2 MPPH|MPPH1|P85B|p85|p85-BETA 19 BCRSignalingPathway

PIK3R3 8503 phosphoinositide-3-kinase regulatory subunit 3 p55|p55-GAMMA|p55PIK 1 BCRSignalingPathway

PIK3CA 5290 phosphatidylinositol-4,5-bisphosphate 3-kinase catalytic subunit alpha CLAPO|CLOVE|CWS5|MCAP|MCM|MCMTC|PI3K|PI3K-alpha|p110-alpha 3 BCRSignalingPathway

PIK3CB 5291 phosphatidylinositol-4,5-bisphosphate 3-kinase catalytic subunit beta P110BETA|PI3K|PI3KBETA|PIK3C1 3 BCRSignalingPathway

PIK3CD 5293 phosphatidylinositol-4,5-bisphosphate 3-kinase catalytic subunit delta APDS|IMD14|P110DELTA|PI3K|p110D 1 BCRSignalingPathway

PIK3CG 5294 phosphatidylinositol-4,5-bisphosphate 3-kinase catalytic subunit gamma PI3CG|PI3K|PI3Kgamma|PIK3|p110gamma|p120-PI3K 7 BCRSignalingPathway

AKT3 10000 AKT serine/threonine kinase 3 MPPH|MPPH2|PKB-GAMMA|PKBG|PRKBG|RAC-PK-gamma|RAC-gamma|STK-2 1 BCRSignalingPathway

AKT1 207 AKT serine/threonine kinase 1 AKT|CWS6|PKB|PKB-ALPHA|PRKBA|RAC|RAC-ALPHA 14 BCRSignalingPathway

AKT2 208 AKT serine/threonine kinase 2 HIHGHH|PKBB|PKBBETA|PRKBB|RAC-BETA 19 BCRSignalingPathway

GSK3B 2932 glycogen synthase kinase 3 beta - 3 BCRSignalingPathway

INPP5D 3635 inositol polyphosphate-5-phosphatase D SHIP|SHIP-1|SHIP1|SIP-145|hp51CN|p150Ship 2 BCRSignalingPathway

CD22 933 CD22 molecule SIGLEC-2|SIGLEC2 19 BCRSignalingPathway

CD72 971 CD72 molecule CD72b|LYB2 9 BCRSignalingPathway

PTPN6 5777 protein tyrosine phosphatase non-receptor type 6 HCP|HCPH|HPTP1C|PTP-1C|SH-PTP1|SHP-1|SHP-1L|SHP1 12 BCRSignalingPathway

LILRB3 11025 leukocyte immunoglobulin like receptor B3 CD85A|HL9|ILT-5|ILT5|LILRA6|LIR-3|LIR3|PIR-B|PIRB 19 BCRSignalingPathway

FCGR2B 2213 Fc fragment of IgG receptor IIb CD32|CD32B|FCG2|FCGR2|FCGR2C|FcRII-c|IGFR2 1 BCRSignalingPathway

RASGRP3 25780 RAS guanyl releasing protein 3 GRP3 2 BCRSignalingPathway

PLCG2 5336 phospholipase C gamma 2 APLAID|FCAS3|PLC-IV|PLC-gamma-2 16 BCRSignalingPathway

PRKCB 5579 protein kinase C beta PKC-beta|PKCB|PKCI(2)|PKCbeta|PRKCB1|PRKCB2 16 BCRSignalingPathway

IFITM1 8519 interferon induced transmembrane protein 1 9-27|CD225|DSPA2a|IFI17|LEU13 11 BCRSignalingPathway

IGH 3492 immunoglobulin heavy locus IGD1|IGH.1@|IGH@|IGHD@|IGHDY1|IGHJ|IGHJ@|IGHV|IGHV@ 14 BCRSignalingPathway

IGHA1 3493 immunoglobulin heavy constant alpha 1 IgA1 14 BCRSignalingPathway

IGHA2 3494 immunoglobulin heavy constant alpha 2 (A2m marker) - 14 BCRSignalingPathway

IGHD 3495 immunoglobulin heavy constant delta - 14 BCRSignalingPathway

IGHD1-1 28510 immunoglobulin heavy diversity 1-1 IGHD11 14 BCRSignalingPathway

IGHD1-14 28508 immunoglobulin heavy diversity 1-14 (non-functional) DM2|IGHD114 14 BCRSignalingPathway

IGHD1-20 28507 immunoglobulin heavy diversity 1-20 IGHD120 14 BCRSignalingPathway

IGHD1-26 28506 immunoglobulin heavy diversity 1-26 IGHD126 14 BCRSignalingPathway

IGHD1-7 28509 immunoglobulin heavy diversity 1-7 DM1|IGHD17 14 BCRSignalingPathway

IGHD2-15 28503 immunoglobulin heavy diversity 2-15 D2|IGHD215 14 BCRSignalingPathway

IGHD2-2 28505 immunoglobulin heavy diversity 2-2 IGHD22 14 BCRSignalingPathway

IGHD2-21 28502 immunoglobulin heavy diversity 2-21 IGHD221 14 BCRSignalingPathway

IGHD2-8 28504 immunoglobulin heavy diversity 2-8 DLR1|IGHD28 14 BCRSignalingPathway

IGHD3-10 28499 immunoglobulin heavy diversity 3-10 DXP'1|IGHD310 14 BCRSignalingPathway

IGHD3-16 28498 immunoglobulin heavy diversity 3-16 IGHD316 14 BCRSignalingPathway

IGHD3-22 28497 immunoglobulin heavy diversity 3-22 IGHD322 14 BCRSignalingPathway

IGHD3-3 28501 immunoglobulin heavy diversity 3-3 DXP4|IGHD33 14 BCRSignalingPathway

IGHD3-9 28500 immunoglobulin heavy diversity 3-9 DXP1|IGHD39 14 BCRSignalingPathway

IGHD4-11 28495 immunoglobulin heavy diversity 4-11 (non-functional) DA1|IGHD411 14 BCRSignalingPathway

IGHD4-17 28494 immunoglobulin heavy diversity 4-17 IGHD417 14 BCRSignalingPathway

IGHD4-23 28493 immunoglobulin heavy diversity 4-23 (non-functional) IGHD423 14 BCRSignalingPathway

IGHD4-4 28496 immunoglobulin heavy diversity 4-4 DA4|IGHD44 14 BCRSignalingPathway

IGHD5-12 28491 immunoglobulin heavy diversity 5-12 DK1|IGHD512 14 BCRSignalingPathway

IGHD5-18 28490 immunoglobulin heavy diversity 5-18 IGHD518 14 BCRSignalingPathway

IGHD5-24 28489 immunoglobulin heavy diversity 5-24 (non-functional) IGHD524 14 BCRSignalingPathway

IGHD5-5 28492 immunoglobulin heavy diversity 5-5 DK4|IGHD55 14 BCRSignalingPathway

IGHD6-13 28487 immunoglobulin heavy diversity 6-13 DN1|IGHD613 14 BCRSignalingPathway

IGHD6-19 28486 immunoglobulin heavy diversity 6-19 IGHD619 14 BCRSignalingPathway

IGHD6-25 28485 immunoglobulin heavy diversity 6-25 IGHD625 14 BCRSignalingPathway

IGHD6-6 28488 immunoglobulin heavy diversity 6-6 D(N4)|IGHD66 14 BCRSignalingPathway

IGHD7-27 28484 immunoglobulin heavy diversity 7-27 DHQ52|IGHD727 14 BCRSignalingPathway

IGHE 3497 immunoglobulin heavy constant epsilon IgE 14 BCRSignalingPathway

IGHG1 3500 immunoglobulin heavy constant gamma 1 (G1m marker) - 14 BCRSignalingPathway

IGHG2 3501 immunoglobulin heavy constant gamma 2 (G2m marker) - 14 BCRSignalingPathway

IGHG3 3502 immunoglobulin heavy constant gamma 3 (G3m marker) IgG3 14 BCRSignalingPathway

IGHG4 3503 immunoglobulin heavy constant gamma 4 (G4m marker) - 14 BCRSignalingPathway

IGHJ1 28483 immunoglobulin heavy joining 1 JH1 14 BCRSignalingPathway

IGHJ2 28481 immunoglobulin heavy joining 2 JH2 14 BCRSignalingPathway

IGHJ3 28479 immunoglobulin heavy joining 3 JH3b 14 BCRSignalingPathway

IGHJ4 28477 immunoglobulin heavy joining 4 JH4b 14 BCRSignalingPathway

IGHJ5 28476 immunoglobulin heavy joining 5 JH5b 14 BCRSignalingPathway

IGHJ6 28475 immunoglobulin heavy joining 6 JH6b 14 BCRSignalingPathway

IGHM 3507 immunoglobulin heavy constant mu AGM1|MU|VH 14 BCRSignalingPathway

IGH 3492 immunoglobulin heavy locus IGD1|IGH.1@|IGH@|IGHD@|IGHDY1|IGHJ|IGHJ@|IGHV|IGHV@ 14 BCRSignalingPathway

IGHV1-18 28468 immunoglobulin heavy variable 1-18 IGHV118 14 BCRSignalingPathway

IGHV1-2 28474 immunoglobulin heavy variable 1-2 IGHV12|V35 14 BCRSignalingPathway

IGHV1-24 28467 immunoglobulin heavy variable 1-24 IGHV124|VH 14 BCRSignalingPathway

IGHV1-3 28473 immunoglobulin heavy variable 1-3 IGHV13|VI-3B 14 BCRSignalingPathway

IGHV1-45 28466 immunoglobulin heavy variable 1-45 IGHV145|VH 14 BCRSignalingPathway

IGHV1-46 28465 immunoglobulin heavy variable 1-46 IGHV146 14 BCRSignalingPathway

IGHV1-58 28464 immunoglobulin heavy variable 1-58 IGHV158|VH 14 BCRSignalingPathway

IGHV1-69 28461 immunoglobulin heavy variable 1-69 IGHV1-E|IGHV169|IGHV1E 14 BCRSignalingPathway

IGHV1-8 28472 immunoglobulin heavy variable 1-8 IGHV18 14 BCRSignalingPathway

IGHV1-38-4 28460 immunoglobulin heavy variable 1-38-4 (non-functional) IGHV1-C|IGHV1C 14 BCRSignalingPathway

IGHV1-69-2 28458 immunoglobulin heavy variable 1-69-2 IGHV1-F|IGHV1F 14 BCRSignalingPathway

IGHV2-26 28455 immunoglobulin heavy variable 2-26 IGHV226|VH 14 BCRSignalingPathway

IGHV2-5 28457 immunoglobulin heavy variable 2-5 IGHV25|VH 14 BCRSignalingPathway

IGHV2-70 28454 immunoglobulin heavy variable 2-70 IGHV270|VH 14 BCRSignalingPathway

IGHV3-11 28450 immunoglobulin heavy variable 3-11 IGHV311|VH 14 BCRSignalingPathway

IGHV3-13 28449 immunoglobulin heavy variable 3-13 IGHV313 14 BCRSignalingPathway

IGHV3-15 28448 immunoglobulin heavy variable 3-15 IGHV315|VH 14 BCRSignalingPathway

IGHV3-16 28447 immunoglobulin heavy variable 3-16 (non-functional) IGHV316|VH 14 BCRSignalingPathway

IGHV3-20 28445 immunoglobulin heavy variable 3-20 IGHV320|VH 14 BCRSignalingPathway

IGHV3-21 28444 immunoglobulin heavy variable 3-21 IGHV321|VH 14 BCRSignalingPathway

IGHV3-23 28442 immunoglobulin heavy variable 3-23 DP47|IGHV323|V3-23|VH26 14 BCRSignalingPathway

IGHV3-30 28439 immunoglobulin heavy variable 3-30 IGHV330|VH 14 BCRSignalingPathway

IGHV3-30-3 57290 immunoglobulin heavy variable 3-30-3 IGHV3-3|IGHV3303 14 BCRSignalingPathway

IGHV3-30-5 89770 immunoglobulin heavy variable 3-30-5 IGHV3-3|IGHV3305 14 BCRSignalingPathway

IGHV3-33 28434 immunoglobulin heavy variable 3-33 IGHV333|VH 14 BCRSignalingPathway

IGHV3-35 28432 immunoglobulin heavy variable 3-35 (non-functional) IGHV335|VH 14 BCRSignalingPathway

IGHV3-38 28429 immunoglobulin heavy variable 3-38 (non-functional) IGHV338|VH 14 BCRSignalingPathway

IGHV3-43 28426 immunoglobulin heavy variable 3-43 IGHV343|VH 14 BCRSignalingPathway

IGHV3-48 28424 immunoglobulin heavy variable 3-48 IGHV348|VH 14 BCRSignalingPathway

IGHV3-49 28423 immunoglobulin heavy variable 3-49 IGHV349|VH 14 BCRSignalingPathway

IGHV3-53 28420 immunoglobulin heavy variable 3-53 IGHV353|VH 14 BCRSignalingPathway

IGHV3-64 28414 immunoglobulin heavy variable 3-64 IGHV364|VH 14 BCRSignalingPathway

IGHV3-66 28412 immunoglobulin heavy variable 3-66 IGHV366|VH 14 BCRSignalingPathway

IGHV3-7 28452 immunoglobulin heavy variable 3-7 IGHV37|VH 14 BCRSignalingPathway

IGHV3-72 28410 immunoglobulin heavy variable 3-72 IGHV372|VH 14 BCRSignalingPathway

IGHV3-73 28409 immunoglobulin heavy variable 3-73 IGHV373|VH 14 BCRSignalingPathway

IGHV3-74 28408 immunoglobulin heavy variable 3-74 IGHV374|VH 14 BCRSignalingPathway

IGHV3-9 28451 immunoglobulin heavy variable 3-9 IGHV39|VH 14 BCRSignalingPathway

IGHV3-38-3 28404 immunoglobulin heavy variable 3-38-3 (non-functional) IGHV3-D|IGHV3D 14 BCRSignalingPathway

IGHV3-69-1 28402 immunoglobulin heavy variable 3-69-1 (pseudogene) IGH|IGHM|IGHV|IGHV3-11|IGHV3-H|IGHV3H|IgVH 14 BCRSignalingPathway

IGHV4-28 28400 immunoglobulin heavy variable 4-28 IGHV428|VH 14 BCRSignalingPathway

IGHV4-30-1 28399 immunoglobulin heavy variable 4-30-1 IGHV4-3 14 BCRSignalingPathway

IGHV4-30-2 28398 immunoglobulin heavy variable 4-30-2 IGHV4-3|IGHV4302 14 BCRSignalingPathway

IGHV4-30-4 28397 immunoglobulin heavy variable 4-30-4 IGHV4-3|IGHV4304 14 BCRSignalingPathway

IGHV4-31 28396 immunoglobulin heavy variable 4-31 IGHV431 14 BCRSignalingPathway

IGHV4-34 28395 immunoglobulin heavy variable 4-34 IGHV434|VH 14 BCRSignalingPathway

IGHV4-39 28394 immunoglobulin heavy variable 4-39 IGHV439|VH 14 BCRSignalingPathway

IGHV4-4 28401 immunoglobulin heavy variable 4-4 IGHV44|VH 14 BCRSignalingPathway

IGHV4-59 28392 immunoglobulin heavy variable 4-59 IGHV459|VH 14 BCRSignalingPathway

IGHV4-61 28391 immunoglobulin heavy variable 4-61 IGHV461|VH 14 BCRSignalingPathway

IGHV4-38-2 28389 immunoglobulin heavy variable 4-38-2 IGHV4-B|IGHV4B 14 BCRSignalingPathway

IGHV5-51 28388 immunoglobulin heavy variable 5-51 IGHV551|VH 14 BCRSignalingPathway

IGHV5-10-1 28386 immunoglobulin heavy variable 5-10-1 IGHV5-A|IGHV5A 14 BCRSignalingPathway

IGHV6-1 28385 immunoglobulin heavy variable 6-1 IGHV61|VH 14 BCRSignalingPathway

IGHV7-4-1 57289 immunoglobulin heavy variable 7-4-1 IGHV7-41|IGHV741 14 BCRSignalingPathway

IGHV7-81 28378 immunoglobulin heavy variable 7-81 (non-functional) IGHV781 14 BCRSignalingPathway

IGK 50802 immunoglobulin kappa locus IGK@ 2 BCRSignalingPathway

IGKC 3514 immunoglobulin kappa constant HCAK1|IGKCD|Km 2 BCRSignalingPathway

IGKDEL 3515 immunoglobulin kappa deleting element or like IGKDE 2 BCRSignalingPathway

IGKJ 7842 - IGKJ@ 2 BCRSignalingPathway

IGKJ1 28950 immunoglobulin kappa joining 1 J1 2 BCRSignalingPathway

IGKJ2 28949 immunoglobulin kappa joining 2 J2 2 BCRSignalingPathway

IGKJ3 28948 immunoglobulin kappa joining 3 J3 2 BCRSignalingPathway

IGKJ4 28947 immunoglobulin kappa joining 4 J4 2 BCRSignalingPathway

IGKJ5 28946 immunoglobulin kappa joining 5 J5 2 BCRSignalingPathway

IGKV@ 3519 - IGKV|IGKV1|IGKV1@|IGKV2|IGKV2@|IGKV3|IGKV3@ 2 BCRSignalingPathway

IGKV1-12 28940 immunoglobulin kappa variable 1-12 IGKV112|L19 2 BCRSignalingPathway

IGKV1-13 28939 immunoglobulin kappa variable 1-13 IGKV113|L18 2 BCRSignalingPathway

IGKV1-16 28938 immunoglobulin kappa variable 1-16 IGKV116|L1 2 BCRSignalingPathway

IGKV1-17 28937 immunoglobulin kappa variable 1-17 A30|IGKV117 2 BCRSignalingPathway

IGKV1-27 28935 immunoglobulin kappa variable 1-27 A20|IGKV127 2 BCRSignalingPathway

IGKV1-33 28933 immunoglobulin kappa variable 1-33 IGKV133|O18 2 BCRSignalingPathway

IGKV1-37 28931 immunoglobulin kappa variable 1-37 (non-functional) IGKV137|O14 2 BCRSignalingPathway

IGKV1-39 28930 immunoglobulin kappa variable 1-39 IGKV139|O12|O12a 2 BCRSignalingPathway

IGKV1-5 28299 immunoglobulin kappa variable 1-5 IGKV|IGKV15|L12|L12a|V1 2 BCRSignalingPathway

IGKV1-6 28943 immunoglobulin kappa variable 1-6 IGKV16|L11 2 BCRSignalingPathway

IGKV1-8 28942 immunoglobulin kappa variable 1-8 IGKV18|L9 2 BCRSignalingPathway

IGKV1-9 28941 immunoglobulin kappa variable 1-9 IGKV19|L8 2 BCRSignalingPathway

IGKV1D-12 28903 immunoglobulin kappa variable 1D-12 IGKV1D12|L19 2 BCRSignalingPathway

IGKV1D-13 28902 immunoglobulin kappa variable 1D-13 IGKV1D13|L18 2 BCRSignalingPathway

IGKV1D-16 28901 immunoglobulin kappa variable 1D-16 IGKV1D16|L15|L15a 2 BCRSignalingPathway

IGKV1D-17 28900 immunoglobulin kappa variable 1D-17 IGKV1D17|L14 2 BCRSignalingPathway

IGKV1D-33 28896 immunoglobulin kappa variable 1D-33 IGKV1D33|O8 2 BCRSignalingPathway

IGKV1D-37 28894 immunoglobulin kappa variable 1D-37 (non-functional) IGKV1D37|O4 2 BCRSignalingPathway

IGKV1D-39 28893 immunoglobulin kappa variable 1D-39 IGKV1D39|O2 2 BCRSignalingPathway

IGKV1D-42 28892 immunoglobulin kappa variable 1D-42 (non-functional) IGKV1D42|L22 2 BCRSignalingPathway

IGKV1D-43 28891 immunoglobulin kappa variable 1D-43 IGKV1D43|L23|L23a 2 BCRSignalingPathway

IGKV1D-8 28904 immunoglobulin kappa variable 1D-8 IGKV1D8|L24|L24a 2 BCRSignalingPathway

IGKV2-24 28923 immunoglobulin kappa variable 2-24 A23|IGKV224 2 BCRSignalingPathway

IGKV2-28 28921 immunoglobulin kappa variable 2-28 A19|IGKV228 2 BCRSignalingPathway

IGKV2-30 28919 immunoglobulin kappa variable 2-30 A17|IGKV230 2 BCRSignalingPathway

IGKV2-40 28916 immunoglobulin kappa variable 2-40 IGKV240|O11|O11a 2 BCRSignalingPathway

IGKV2D-24 28885 immunoglobulin kappa variable 2D-24 (non-functional) A7|IGKV2D24 2 BCRSignalingPathway

IGKV2D-28 28883 immunoglobulin kappa variable 2D-28 A3|IGKV2D28 2 BCRSignalingPathway

IGKV2D-29 28882 immunoglobulin kappa variable 2D-29 A2a|A2c|IGKV2D29 2 BCRSignalingPathway

IGKV2D-30 28881 immunoglobulin kappa variable 2D-30 A1|IGKV2D30 2 BCRSignalingPathway

IGKV2D-40 28878 immunoglobulin kappa variable 2D-40 IGKV2D40|O1 2 BCRSignalingPathway

IGKV3-11 28914 immunoglobulin kappa variable 3-11 IGKV311|L6 2 BCRSignalingPathway

IGKV3-15 28913 immunoglobulin kappa variable 3-15 IGKV315|L2 2 BCRSignalingPathway

IGKV3-20 28912 immunoglobulin kappa variable 3-20 13K18|A27|IGKV320 2 BCRSignalingPathway

IGKV3-7 28915 immunoglobulin kappa variable 3-7 (non-functional) IGKV37|L10|L10a|Vh 2 BCRSignalingPathway

IGKV3D-11 28876 immunoglobulin kappa variable 3D-11 IGKV3D11|L20 2 BCRSignalingPathway

IGKV3D-15 28875 immunoglobulin kappa variable 3D-15 IGKV3D15|L16|L16a|L16b|L16c 2 BCRSignalingPathway

IGKV3D-20 28874 immunoglobulin kappa variable 3D-20 A11|A11a|IGKV3D20 2 BCRSignalingPathway

IGKV3D-7 28877 immunoglobulin kappa variable 3D-7 IGKV3D7|L25 2 BCRSignalingPathway

IGKV4-1 28908 immunoglobulin kappa variable 4-1 B3|IGKV41 2 BCRSignalingPathway

IGKV5-2 28907 immunoglobulin kappa variable 5-2 B2|IGKV52 2 BCRSignalingPathway

IGKV6-21 28906 immunoglobulin kappa variable 6-21 (non-functional) A26|IGKV621 2 BCRSignalingPathway

IGKV6D-21 28870 immunoglobulin kappa variable 6D-21 (non-functional) A10|IGKV6D21 2 BCRSignalingPathway

IGKV6D-41 28869 immunoglobulin kappa variable 6D-41 (non-functional) A14 2 BCRSignalingPathway

IGL 3535 immunoglobulin lambda locus IGL@|IGLC6 22 BCRSignalingPathway

IGLC1 3537 immunoglobulin lambda constant 1 IGLC 22 BCRSignalingPathway

IGLC2 3538 immunoglobulin lambda constant 2 IGLC 22 BCRSignalingPathway

IGLC3 3539 immunoglobulin lambda constant 3 (Kern-Oz+ marker) IGLC 22 BCRSignalingPathway

IGLC6 3542 immunoglobulin lambda constant 6 IGLC 22 BCRSignalingPathway

IGLC7 28834 immunoglobulin lambda constant 7 C7 22 BCRSignalingPathway

IGLJ 8217 - IGLJ@ 22 BCRSignalingPathway

IGLJ1 28833 immunoglobulin lambda joining 1 J1 22 BCRSignalingPathway

IGLJ2 28832 immunoglobulin lambda joining 2 J2 22 BCRSignalingPathway

IGLJ3 28831 immunoglobulin lambda joining 3 J3 22 BCRSignalingPathway

IGLJ4 28830 immunoglobulin lambda joining 4 (non-functional) - 22 BCRSignalingPathway

IGLJ5 28829 immunoglobulin lambda joining 5 (non-functional) - 22 BCRSignalingPathway

IGLJ6 28828 immunoglobulin lambda joining 6 - 22 BCRSignalingPathway

IGLJ7 28827 immunoglobulin lambda joining 7 J7 22 BCRSignalingPathway

IGLV@ 3546 - IGLV 22 BCRSignalingPathway

IGLV1-36 28826 immunoglobulin lambda variable 1-36 IGLV136|V1-11 22 BCRSignalingPathway

IGLV1-40 28825 immunoglobulin lambda variable 1-40 IGLV140|V1-13 22 BCRSignalingPathway

IGLV1-44 28823 immunoglobulin lambda variable 1-44 IGLV144|V1-16 22 BCRSignalingPathway

IGLV1-47 28822 immunoglobulin lambda variable 1-47 IGLV147|V1-17 22 BCRSignalingPathway

IGLV1-50 28821 immunoglobulin lambda variable 1-50 (non-functional) IGLV150|V1-18 22 BCRSignalingPathway

IGLV1-51 28820 immunoglobulin lambda variable 1-51 IGLV151|V1-19 22 BCRSignalingPathway

IGLV10-54 28772 immunoglobulin lambda variable 10-54 IGLV1054|V1-20 22 BCRSignalingPathway

IGLV11-55 28770 immunoglobulin lambda variable 11-55 (non-functional) IGLV1155|V4-6 22 BCRSignalingPathway

IGLV2-11 28816 immunoglobulin lambda variable 2-11 IGLV211|V1-3 22 BCRSignalingPathway

IGLV2-14 28815 immunoglobulin lambda variable 2-14 IGLV214|V1-4 22 BCRSignalingPathway

IGLV2-18 28814 immunoglobulin lambda variable 2-18 IGLV218|V1-5 22 BCRSignalingPathway

IGLV2-23 28813 immunoglobulin lambda variable 2-23 IGLV223|V1-7 22 BCRSignalingPathway

IGLV2-33 28811 immunoglobulin lambda variable 2-33 (non-functional) IGLV233|V1-9 22 BCRSignalingPathway

IGLV2-8 28817 immunoglobulin lambda variable 2-8 IGLV28|V1-2 22 BCRSignalingPathway

IGLV3-1 28809 immunoglobulin lambda variable 3-1 IGLV31|V2-1 22 BCRSignalingPathway

IGLV3-10 28803 immunoglobulin lambda variable 3-10 IGLV310|V2-7 22 BCRSignalingPathway

IGLV3-12 28802 immunoglobulin lambda variable 3-12 IGLV312|V2-8 22 BCRSignalingPathway

IGLV3-16 28799 immunoglobulin lambda variable 3-16 IGLV316|V2-11 22 BCRSignalingPathway

IGLV3-19 28797 immunoglobulin lambda variable 3-19 IGLV319|V2-13|VL3L 22 BCRSignalingPathway

IGLV3-21 28796 immunoglobulin lambda variable 3-21 IGLV321|V2-14 22 BCRSignalingPathway

IGLV3-22 28795 immunoglobulin lambda variable 3-22 IGLV322|V2-15 22 BCRSignalingPathway

IGLV3-25 28793 immunoglobulin lambda variable 3-25 IGLV325|V2-17 22 BCRSignalingPathway

IGLV3-27 28791 immunoglobulin lambda variable 3-27 IGLV327|V2-19 22 BCRSignalingPathway

IGLV3-32 28787 immunoglobulin lambda variable 3-32 (non-functional) IGLV332|V2-23P 22 BCRSignalingPathway

IGLV3-9 28804 immunoglobulin lambda variable 3-9 IGLV39|V2-6 22 BCRSignalingPathway

IGLV4-3 28786 immunoglobulin lambda variable 4-3 IGLV43|V5-1 22 BCRSignalingPathway

IGLV4-60 28785 immunoglobulin lambda variable 4-60 IGLV460|V5-4 22 BCRSignalingPathway

IGLV4-69 28784 immunoglobulin lambda variable 4-69 IGLV469|V5-6 22 BCRSignalingPathway

IGLV5-37 28783 immunoglobulin lambda variable 5-37 IGLV537|V4-1 22 BCRSignalingPathway

IGLV5-39 28782 immunoglobulin lambda variable 5-39 IGLV539 22 BCRSignalingPathway

IGLV5-45 28781 immunoglobulin lambda variable 5-45 IGLV545|V4-2 22 BCRSignalingPathway

IGLV5-48 28780 immunoglobulin lambda variable 5-48 (non-functional) IGLV548|V4-3 22 BCRSignalingPathway

IGLV5-52 28779 immunoglobulin lambda variable 5-52 IGLV552|V4-4 22 BCRSignalingPathway

IGLV6-57 28778 immunoglobulin lambda variable 6-57 IGLV657|V1-22 22 BCRSignalingPathway

IGLV7-43 28776 immunoglobulin lambda variable 7-43 IGLV743|V3-2 22 BCRSignalingPathway

IGLV7-46 28775 immunoglobulin lambda variable 7-46 IGLV746|V3-3 22 BCRSignalingPathway

IGLV8-61 28774 immunoglobulin lambda variable 8-61 IGLV861|V3-4 22 BCRSignalingPathway

IGLV9-49 28773 immunoglobulin lambda variable 9-49 IGLV949|V5-2 22 BCRSignalingPathway

C3 718 complement C3 AHUS5|ARMD9|ASP|C3a|C3b|CPAMD1|HEL-S-62p 19 Chemokines

C5 727 complement C5 C5D|C5a|C5b|CPAMD4|ECLZB 9 Chemokines

CAMP 820 cathelicidin antimicrobial peptide CAP-18|CAP18|CRAMP|FALL-39|FALL39|HSD26|LL37 3 Chemokines

CCL1 6346 C-C motif chemokine ligand 1 I-309|P500|SCYA1|SISe|TCA3 17 Chemokines

CCL11 6356 C-C motif chemokine ligand 11 SCYA11 17 Chemokines

CCL13 6357 C-C motif chemokine ligand 13 CKb10|MCP-4|NCC-1|NCC1|SCYA13|SCYL1 17 Chemokines

CCL14 6358 C-C motif chemokine ligand 14 CC-1|CC-3|CKB1|HCC-1|HCC-1(1-74)|HCC-1/HCC-3|HCC-3|MCIF|NCC-2|NCC2|SCYA14|SCYL2|SY14 17 Chemokines

CCL15-CCL14 348249 CCL15-CCL14 readthrough (NMD candidate) CCL15|HCC-2|LKN-1|MIP-5|MIP5|Mrp-2b|NCC-3|NCC3|SCYA15 17 Chemokines

CCL15 6359 C-C motif chemokine ligand 15 HCC-2|HMRP-2B|LKN-1|LKN1|MIP-1 delta|MIP-1D|MIP-5|MRP-2B|NCC-3|NCC3|SCYA15|SCYL3|SY15 17 Chemokines

CCL16 6360 C-C motif chemokine ligand 16 CKb12|HCC-4|ILINCK|LCC-1|LEC|LMC|Mtn-1|NCC-4|NCC4|SCYA16|SCYL4 17 Chemokines

CCL17 6361 C-C motif chemokine ligand 17 A-152E5.3|ABCD-2|SCYA17|TARC 16 Chemokines

CCL18 6362 C-C motif chemokine ligand 18 AMAC-1|AMAC1|CKb7|DC-CK1|DCCK1|MIP-4|PARC|SCYA18 17 Chemokines

CCL19 6363 C-C motif chemokine ligand 19 CKb11|ELC|MIP-3b|MIP3B|SCYA19 9 Chemokines

CCL2 6347 C-C motif chemokine ligand 2 GDCF-2|HC11|HSMCR30|MCAF|MCP-1|MCP1|SCYA2|SMC-CF 17 Chemokines

CCL20 6364 C-C motif chemokine ligand 20 CKb4|Exodus|LARC|MIP-3-alpha|MIP-3a|MIP3A|SCYA20|ST38 2 Chemokines

CCL21 6366 C-C motif chemokine ligand 21 6Ckine|CKb9|ECL|SCYA21|SLC|TCA4 9 Chemokines

CCL22 6367 C-C motif chemokine ligand 22 A-152E5.1|ABCD-1|DC/B-CK|MDC|SCYA22|STCP-1 16 Chemokines

CCL23 6368 C-C motif chemokine ligand 23 CK-BETA-8|CKb8|Ckb-8|Ckb-8-1|MIP-3|MIP3|MPIF-1|SCYA23|hmrp-2a 17 Chemokines

CCL24 6369 C-C motif chemokine ligand 24 Ckb-6|MPIF-2|MPIF2|SCYA24 7 Chemokines

CCL25 6370 C-C motif chemokine ligand 25 Ckb15|SCYA25|TECK 19 Chemokines

CCL26 10344 C-C motif chemokine ligand 26 IMAC|MIP-4a|MIP-4alpha|SCYA26|TSC-1 7 Chemokines

CCL27 10850 C-C motif chemokine ligand 27 ALP|CTACK|CTAK|ESKINE|ILC|PESKY|SCYA27 9 Chemokines

CCL28 56477 C-C motif chemokine ligand 28 CCK1|MEC|SCYA28 5 Chemokines

CCL3 6348 C-C motif chemokine ligand 3 G0S19-1|LD78ALPHA|MIP-1-alpha|MIP1A|SCYA3 17 Chemokines

CCL3L1 6349 C-C motif chemokine ligand 3 like 1 464.2|D17S1718|G0S19-2|LD78|LD78-beta(1-70)|LD78BETA|MIP1AP|SCYA3L|SCYA3L1 17 Chemokines

CCL3P1 390788 C-C motif chemokine ligand 3 pseudogene 1 CCL3L2|G0S19-3|LD78gamma|SCYA3L2 17 Chemokines

CCL3L3 414062 C-C motif chemokine ligand 3 like 3 464.2|D17S1718|G0S19-2|LD78|LD78BETA|SCYA3L|SCYA3L1 17 Chemokines

CCL4 6351 C-C motif chemokine ligand 4 ACT2|AT744.1|G-26|HC21|LAG-1|LAG1|MIP-1-beta|MIP1B|MIP1B1|SCYA2|SCYA4 17 Chemokines

CCL4L2 9560 C-C motif chemokine ligand 4 like 2 AT744.2|CCL4L|SCYA4L|SCYQ4L2 17 Chemokines

CCL4L1 388372 C-C motif chemokine ligand 4 like 1 AT744.2|CCL4L|LAG-1|LAG1|MIP-1-beta|SCYA4L|SCYA4L1|SCYA4L2 17 Chemokines

CCL5 6352 C-C motif chemokine ligand 5 D17S136E|RANTES|SCYA5|SIS-delta|SISd|TCP228|eoCP 17 Chemokines

CCL7 6354 C-C motif chemokine ligand 7 FIC|MARC|MCP-3|MCP3|NC28|SCYA6|SCYA7 17 Chemokines

CCL8 6355 C-C motif chemokine ligand 8 HC14|MCP-2|MCP2|SCYA10|SCYA8 17 Chemokines

CKLF 51192 chemokine like factor C32|CKLF1|CKLF2|CKLF3|CKLF4|HSPC224|UCK-1 16 Chemokines

CMA1 1215 chymase 1 CYH|MCT1|chymase 14 Chemokines

CTSG 1511 cathepsin G CATG|CG 14 Chemokines

CX3CL1 6376 C-X3-C motif chemokine ligand 1 ABCD-3|C3Xkine|CXC3|CXC3C|NTN|NTT|SCYD1|fractalkine|neurotactin 16 Chemokines

CXCL1 2919 C-X-C motif chemokine ligand 1 FSP|GRO1|GROa|MGSA|MGSA-a|NAP-3|SCYB1 4 Chemokines

CXCL10 3627 C-X-C motif chemokine ligand 10 C7|IFI10|INP10|IP-10|SCYB10|crg-2|gIP-10|mob-1 4 Chemokines

CXCL11 6373 C-X-C motif chemokine ligand 11 H174|I-TAC|IP-9|IP9|SCYB11|SCYB9B|b-R1 4 Chemokines

CXCL12 6387 C-X-C motif chemokine ligand 12 IRH|PBSF|SCYB12|SDF1|TLSF|TPAR1 10 Chemokines

CXCL13 10563 C-X-C motif chemokine ligand 13 ANGIE|ANGIE2|BCA-1|BCA1|BLC|BLR1L|SCYB13 4 Chemokines

CXCL14 9547 C-X-C motif chemokine ligand 14 BMAC|BRAK|KEC|KS1|MIP-2g|MIP2G|NJAC|SCYB14 5 Chemokines

CXCL16 58191 C-X-C motif chemokine ligand 16 CXCLG16|SR-PSOX|SRPSOX 17 Chemokines

CXCL17 284340 C-X-C motif chemokine ligand 17 DMC|Dcip1|UNQ473|VCC-1|VCC1 19 Chemokines

CXCL2 2920 C-X-C motif chemokine ligand 2 CINC-2a|GRO2|GROb|MGSA-b|MIP-2a|MIP2|MIP2A|SCYB2 4 Chemokines

CXCL3 2921 C-X-C motif chemokine ligand 3 CINC-2b|GRO3|GROg|MIP-2b|MIP2B|SCYB3 4 Chemokines

CXCL5 6374 C-X-C motif chemokine ligand 5 ENA-78|SCYB5 4 Chemokines

CXCL6 6372 C-X-C motif chemokine ligand 6 CKA-3|GCP-2|GCP2|SCYB6 4 Chemokines

CXCL9 4283 C-X-C motif chemokine ligand 9 CMK|Humig|MIG|SCYB9|crg-10 4 Chemokines

CCN1 3491 cellular communication network factor 1 CYR61|GIG1|IGFBP10 1 Chemokines

DEFA1 1667 defensin alpha 1 DEF1|DEFA2|HNP-1|HP-1|HP1|MRS 8 Chemokines

DEFA3 1668 defensin alpha 3 DEF3|HNP-3|HNP3|HP-3|HP3 8 Chemokines

DEFA5 1670 defensin alpha 5 DEF5|HD-5 8 Chemokines

DEFB1 1672 defensin beta 1 BD1|DEFB-1|DEFB101|HBD1 8 Chemokines

DEFB103B 55894 defensin beta 103B BD-3|DEFB-3|DEFB103|DEFB3|HBD-3|HBD3|HBP-3|HBP3 8 Chemokines

DEFB104A 140596 defensin beta 104A BD-4|DEFB-4|DEFB104|DEFB4|hBD-4 8 Chemokines

DEFB4A 1673 defensin beta 4A BD-2|DEFB-2|DEFB102|DEFB2|DEFB4|HBD-2|SAP1 8 Chemokines

EDN1 1906 endothelin 1 ARCND3|ET1|HDLCQ7|PPET1|QME 6 Chemokines

EDN2 1907 endothelin 2 ET-2|ET2|PPET2 1 Chemokines

EDN3 1908 endothelin 3 ET-3|ET3|HSCR4|PPET3|WS4B 20 Chemokines

FGF10 2255 fibroblast growth factor 10 - 5 Chemokines

FGF2 2247 fibroblast growth factor 2 BFGF|FGF-2|FGFB|HBGF-2 4 Chemokines

HTN3 3347 histatin 3 HIS2|HTN2|HTN5|PB 4 Chemokines

CXCL8 3576 C-X-C motif chemokine ligand 8 GCP-1|GCP1|IL8|LECT|LUCT|LYNAP|MDNCF|MONAP|NAF|NAP-1|NAP1|SCYB8 4 Chemokines

LECT2 3950 leukocyte cell derived chemotaxin 2 chm-II|chm2 5 Chemokines

PF4 5196 platelet factor 4 CXCL4|PF-4|SCYB4 4 Chemokines

PF4V1 5197 platelet factor 4 variant 1 CXCL4L1|CXCL4V1|PF4-ALT|PF4A|SCYB4V1 4 Chemokines

PLAU 5328 plasminogen activator, urokinase ATF|BDPLT5|QPD|UPA|URK|u-PA 10 Chemokines

PPBP 5473 pro-platelet basic protein B-TG1|Beta-TG|CTAP-III|CTAP3|CTAPIII|CXCL7|LA-PF4|LDGF|MDGF|NAP-2|PBP|SCYB7|TC1|TC2|TGB|TGB1|THBGB|THBGB1 4 Chemokines

PPBPP1 728045 pro-platelet basic protein pseudogene 1 PPBPL1|TGB2 4 Chemokines

PROK2 60675 prokineticin 2 BV8|HH4|KAL4|MIT1|PK2 3 Chemokines

RNASE2 6036 ribonuclease A family member 2 EDN|RAF3|RNS2 14 Chemokines

SAA1 6288 serum amyloid A1 PIG4|SAA|SAA2|TP53I4 11 Chemokines

SAA2 6289 serum amyloid A2 SAA|SAA1 11 Chemokines

SBDS 51119 SBDS ribosome maturation factor CGI-97|SDS|SWDS 7 Chemokines

SEMA3A 10371 semaphorin 3A COLL1|HH16|Hsema-I|Hsema-III|SEMA1|SEMAD|SEMAIII|SEMAL|SemD|coll-1 7 Chemokines

SEMA3B 7869 semaphorin 3B LUCA-1|SEMA5|SEMAA|SemA|semaV 3 Chemokines

SEMA3C 10512 semaphorin 3C SEMAE|SemE 7 Chemokines

SEMA3D 223117 semaphorin 3D Sema-Z2|coll-2 7 Chemokines

SEMA3E 9723 semaphorin 3E M-SEMAH|M-SemaK|SEMAH|coll-5 7 Chemokines

SEMA3F 6405 semaphorin 3F SEMA-IV|SEMA4|SEMAK 3 Chemokines

SEMA3G 56920 semaphorin 3G sem2 3 Chemokines

SEMA4A 64218 semaphorin 4A CORD10|RP35|SEMAB|SEMB 1 Chemokines

SEMA4B 10509 semaphorin 4B SEMAC|SemC 15 Chemokines

SEMA4C 54910 semaphorin 4C M-SEMA-F|SEMACL1|SEMAF|SEMAI 2 Chemokines

SEMA4D 10507 semaphorin 4D A8|BB18|C9orf164|CD100|COLL4|GR3|M-sema-G|SEMAJ|coll-4 9 Chemokines

SEMA4F 10505 ssemaphorin 4F M-SEMA|PRO2353|S4F|SEMAM|SEMAW|m-Sema-M 2 Chemokines

SEMA4G 57715 semaphorin 4G - 10 Chemokines

SEMA5A 9037 semaphorin 5A SEMAF|semF 5 Chemokines

SEMA5B 54437 semaphorin 5B SEMAG|SemG 3 Chemokines

SEMA6A 57556 semaphorin 6A HT018|SEMA|SEMA6A1|SEMAQ|VIA 5 Chemokines

SEMA6B 10501 semaphorin 6B EPM11|SEM-SEMA-Y|SEMA-VIB|SEMAN|semaZ 19 Chemokines

SEMA6C 10500 semaphorin 6C SEMAY|m-SemaY|m-SemaY2 1 Chemokines

SEMA6D 80031 semaphorin 6D - 15 Chemokines

SEMA7A 8482 semaphorin 7A (John Milton Hagen blood group) CD108|CDw108|H-SEMA-K1|H-Sema-L|JMH|SEMAK1|SEMAL 15 Chemokines

SLIT1 6585 slit guidance ligand 1 MEGF4|SLIL1|SLIT-1|SLIT3 10 Chemokines

SLIT2 9353 slit guidance ligand 2 SLIL3|Slit-2 4 Chemokines

TNC 3371 tenascin C 150-225|DFNA56|GMEM|GP|HXB|JI|TN|TN-C 9 Chemokines

TYMP 1890 thymidine phosphorylase ECGF|ECGF1|MEDPS1|MNGIE|MTDPS1|PDECGF|TP|hPD-ECGF 22 Chemokines

XCL1 6375 X-C motif chemokine ligand 1 ATAC|LPTN|LTN|SCM-1|SCM-1a|SCM1|SCM1A|SCYC1 1 Chemokines

XCL2 6846 X-C motif chemokine ligand 2 SCM-1b|SCM1B|SCYC2 1 Chemokines

C5AR1 728 complement C5a receptor 1 C5A|C5AR|C5R1|CD88 19 Chemokine_Receptors

ACKR2 1238 atypical chemokine receptor 2 CCBP2|CCR10|CCR9|CMKBR9|D6|hD6 3 Chemokine_Receptors

CCR1 1230 C-C motif chemokine receptor 1 CD191|CKR-1|CKR1|CMKBR1|HM145|MIP1aR|SCYAR1 3 Chemokine_Receptors

CCR10 2826 C-C motif chemokine receptor 10 GPR2 17 Chemokine_Receptors

CCR3 1232 C-C motif chemokine receptor 3 C C CKR3|CC-CKR-3|CD193|CKR 3|CKR3|CMKBR3 3 Chemokine_Receptors

CCR4 1233 C-C motif chemokine receptor 4 CC-CKR-4|CD194|CKR4|CMKBR4|ChemR13|HGCN:14099|K5-5 3 Chemokine_Receptors

CCR5 1234 C-C motif chemokine receptor 5 CC-CKR-5|CCCKR5|CCR-5|CD195|CKR-5|CKR5|CMKBR5|IDDM22 3 Chemokine_Receptors

CCR6 1235 C-C motif chemokine receptor 6 BN-1|C-C CKR-6|CC-CKR-6|CCR-6|CD196|CKR-L3|CKRL3|CMKBR6|DCR2|DRY6|GPR29|GPRCY4|STRL22 6 Chemokine_Receptors

CCR7 1236 C-C motif chemokine receptor 7 BLR2|CC-CKR-7|CCR-7|CD197|CDw197|CMKBR7|EBI1 17 Chemokine_Receptors

CCR8 1237 C-C motif chemokine receptor 8 CC-CKR-8|CCR-8|CDw198|CKRL1|CMKBR8|CMKBRL2|CY6|GPRCY6|TER1 3 Chemokine_Receptors

CCR9 10803 C-C motif chemokine receptor 9 CC-CKR-9|CDw199|GPR-9-6|GPR28 3 Chemokine_Receptors

ACKR4 51554 atypical chemokine receptor 4 CC-CKR-11|CCBP2|CCR-11|CCR10|CCR11|CCRL1|CCX CKR|CCX-CKR|CKR-11|PPR1|VSHK1 3 Chemokine_Receptors

CCRL2 9034 C-C motif chemokine receptor like 2 ACKR5|CKRX|CRAM|CRAM-A|CRAM-B|HCR 3 Chemokine_Receptors

CMKLR1 1240 chemerin chemokine-like receptor 1 CHEMERINR|ChemR23|DEZ|RVER1 12 Chemokine_Receptors

CX3CR1 1524 C-X3-C motif chemokine receptor 1 CCRL1|CMKBRL1|CMKDR1|GPR13|GPRV28|V28 3 Chemokine_Receptors

CXCR3 2833 C-X-C motif chemokine receptor 3 CD182|CD183|CKR-L2|CMKAR3|GPR9|IP10-R|Mig-R|MigR X Chemokine_Receptors

CXCR4 7852 C-X-C motif chemokine receptor 4 CD184|D2S201E|FB22|HM89|HSY3RR|LAP-3|LAP3|LCR1|LESTR|NPY3R|NPYR|NPYRL|NPYY3R|WHIM|WHIMS 2 Chemokine_Receptors

CXCR5 643 C-X-C motif chemokine receptor 5 BLR1|CD185|MDR15 11 Chemokine_Receptors

CXCR6 10663 C-X-C motif chemokine receptor 6 BONZO|CD186|STRL33|TYMSTR 3 Chemokine_Receptors

ACKR3 57007 atypical chemokine receptor 3 CMKOR1|CXC-R7|CXCR-7|CXCR7|GPR159|RDC-1|RDC1 2 Chemokine_Receptors

CYSLTR1 10800 cysteinyl leukotriene receptor 1 CYSLT1|CYSLT1R|CYSLTR|HMTMF81 X Chemokine_Receptors

CYSLTR2 57105 cysteinyl leukotriene receptor 2 CYSLT2|CYSLT2R|GPCR21|HG57|HPN321|KPG_011|PSEC0146|hGPCR21 13 Chemokine_Receptors

ACKR1 2532 atypical chemokine receptor 1 (Duffy blood group) CCBP1|CD234|DARC|DARC/ACKR1|Dfy|FY|GPD|GpFy|WBCQ1 1 Chemokine_Receptors

EDNRA 1909 endothelin receptor type A ET-A|ETA|ETA-R|ETAR|ETRA|MFDA|hET-AR 4 Chemokine_Receptors

EDNRB 1910 endothelin receptor type B ABCDS|ET-B|ET-BR|ETB|ETB1|ETBR|ETRB|HSCR|HSCR2|WS4A 13 Chemokine_Receptors

FPR1 2357 formyl peptide receptor 1 FMLP|FPR 19 Chemokine_Receptors

FPR2 2358 formyl peptide receptor 2 ALXR|FMLP-R-II|FMLPX|FPR2A|FPRH1|FPRH2|FPRL1|HM63|LXA4R 19 Chemokine_Receptors

FPR2 2358 formyl peptide receptor 2 ALXR|FMLP-R-II|FMLPX|FPR2A|FPRH1|FPRH2|FPRL1|HM63|LXA4R 19 Chemokine_Receptors

GPR17 2840 G protein-coupled receptor 17 - 2 Chemokine_Receptors

GPR32 2854 G protein-coupled receptor 32 RVDR1 19 Chemokine_Receptors

GPR33 2856 G protein-coupled receptor 33 - 14 Chemokine_Receptors

PTGDR2 11251 prostaglandin D2 receptor 2 CD294|CRTH2|DL1R|DP2|GPR44 11 Chemokine_Receptors

C5AR2 27202 complement component 5a receptor 2 C5L2|GPF77|GPR77 19 Chemokine_Receptors

CXCR1 3577 C-X-C motif chemokine receptor 1 C-C|C-C-CKR-1|CD128|CD181|CDw128a|CKR-1|CMKAR1|IL8R1|IL8RA|IL8RBA 2 Chemokine_Receptors

CXCR2 3579 C-X-C motif chemokine receptor 2 CD182|CDw128b|CMKAR2|IL8R2|IL8RA|IL8RB 2 Chemokine_Receptors

LTB4R 1241 leukotriene B4 receptor BLT1|BLTR|CMKRL1|GPR16|LTB4R1|LTBR1|P2RY7|P2Y7 14 Chemokine_Receptors

LTB4R2 56413 leukotriene B4 receptor 2 BLT2|BLTR2|JULF2|KPG_004|LTB4-R 2|LTB4-R2|NOP9 14 Chemokine_Receptors

PLAUR 5329 plasminogen activator, urokinase receptor CD87|U-PAR|UPAR|URKR 19 Chemokine_Receptors

PLXNA1 5361 plexin A1 NOV|NOVP|PLEXIN-A1|PLXN1 3 Chemokine_Receptors

PLXNA2 5362 plexin A2 OCT|PLXN2 1 Chemokine_Receptors

PLXNA3 55558 plexin A3 6.3|HSSEXGENE|PLXN3|PLXN4|XAP-6 X Chemokine_Receptors

PLXNA4 91584 plexin A4 FAYV2820|PLEXA4|PLXNA4A|PLXNA4B|PRO34003 7 Chemokine_Receptors

PLXNB1 5364 plexin B1 PLEXIN-B1|PLXN5|SEP 3 Chemokine_Receptors

PLXNB2 23654 plexin B2 MM1|Nbla00445|PLEXB2|dJ402G11.3 22 Chemokine_Receptors

PLXNB3 5365 plexin B3 PLEXB3|PLEXR|PLXN6 X Chemokine_Receptors

PLXNC1 10154 plexin C1 CD232|PLXN-C1|VESPR 12 Chemokine_Receptors

PLXND1 23129 plexin D1 PLEXD1 3 Chemokine_Receptors

PTAFR 5724 platelet activating factor receptor PAFR 1 Chemokine_Receptors

ROBO1 6091 roundabout guidance receptor 1 DUTT1|SAX3 3 Chemokine_Receptors

ROBO2 6092 roundabout guidance receptor 2 SAX3 3 Chemokine_Receptors

ROBO3 64221 roundabout guidance receptor 3 HGPPS|HGPPS1|HGPS|RBIG1|RIG1 11 Chemokine_Receptors

RXFP3 51289 relaxin family peptide receptor 3 GPCR135|RLN3R1|RXFPR3|SALPR 5 Chemokine_Receptors

XCR1 2829 X-C motif chemokine receptor 1 CCXCR1|GPR5 3 Chemokine_Receptors

ADIPOQ 9370 adiponectin, C1Q and collagen domain containing ACDC|ACRP30|ADIPQTL1|ADPN|APM-1|APM1|GBP28 3 Cytokines

ADM 133 adrenomedullin AM|PAMP 11 Cytokines

ADM2 79924 adrenomedullin 2 AM2|dJ579N16.4 22 Cytokines

AGRP 181 agouti related neuropeptide AGRT|ART|ASIP2 16 Cytokines

AGT 183 angiotensinogen ANHU|SERPINA8|hFLT1 1 Cytokines

AMBN 258 ameloblastin AI1F 4 Cytokines

AMELX 265 amelogenin X-linked AI1E|AIH1|ALGN|AMG|AMGL|AMGX X Cytokines

AMH 268 anti-Mullerian hormone MIF|MIS 19 Cytokines

ANGPTL5 253935 angiopoietin like 5 - 11 Cytokines

ANGPTL7 10218 angiopoietin like 7 AngX|CDT6|dJ647M16.1 1 Cytokines

APLN 8862 apelin APEL|XNPEP2 X Cytokines

AREG 374 amphiregulin AR|AREGB|CRDGF|SDGF 4 Cytokines

MANF 7873 mesencephalic astrocyte derived neurotrophic factor ARMET|ARP 3 Cytokines

CDNF 441549 cerebral dopamine neurotrophic factor ARMETL1 10 Cytokines

ARTN 9048 artemin ART|ENOVIN|EVN|NBN 1 Cytokines

AVP 551 arginine vasopressin ADH|ARVP|AVP-NPII|AVRP|VP 20 Cytokines

AZU1 566 azurocidin 1 AZAMP|AZU|CAP37|HBP|HUMAZUR|NAZC|hHBP 19 Cytokines

BDNF 627 brain derived neurotrophic factor ANON2|BULN2 11 Cytokines

BMP1 649 bone morphogenetic protein 1 OI13|PCOLC|PCP|PCP2|TLD 8 Cytokines

BMP10 27302 bone morphogenetic protein 10 - 2 Cytokines

BMP15 9210 bone morphogenetic protein 15 GDF9B|ODG2|POF4 X Cytokines

BMP2 650 bone morphogenetic protein 2 BDA2|BMP2A|SSFSC 20 Cytokines

BMP3 651 bone morphogenetic protein 3 BMP-3A 4 Cytokines

BMP4 652 bone morphogenetic protein 4 BMP2B|BMP2B1|MCOPS6|OFC11|ZYME 14 Cytokines

BMP5 653 bone morphogenetic protein 5 - 6 Cytokines

BMP6 654 bone morphogenetic protein 6 VGR|VGR1 6 Cytokines

BMP7 655 bone morphogenetic protein 7 OP-1 20 Cytokines

BMP8A 353500 bone morphogenetic protein 8a OP-2 1 Cytokines

BMP8B 656 bone morphogenetic protein 8b BMP8|OP2 1 Cytokines

BTC 685 betacellulin - 4 Cytokines

MYDGF 56005 myeloid derived growth factor C19orf10|EUROIMAGE1875335|IL25|IL27|IL27w|R33729_1|SF20 19 Cytokines

C3 718 complement C3 AHUS5|ARMD9|ASP|C3a|C3b|CPAMD1|HEL-S-62p 19 Cytokines

C5 727 complement C5 C5D|C5a|C5b|CPAMD4|ECLZB 9 Cytokines

CALCA 796 calcitonin related polypeptide alpha CALC1|CGRP|CGRP-I|CGRP-alpha|CGRP1|CT|KC|PCT 11 Cytokines

CALCB 797 calcitonin related polypeptide beta CALC2|CGRP-II|CGRP2 11 Cytokines

CAMP 820 cathelicidin antimicrobial peptide CAP-18|CAP18|CRAMP|FALL-39|FALL39|HSD26|LL37 3 Cytokines

CAT 847 catalase - 11 Cytokines

CCK 885 cholecystokinin - 3 Cytokines

CCL1 6346 C-C motif chemokine ligand 1 I-309|P500|SCYA1|SISe|TCA3 17 Cytokines

CCL11 6356 C-C motif chemokine ligand 11 SCYA11 17 Cytokines

CCL13 6357 C-C motif chemokine ligand 13 CKb10|MCP-4|NCC-1|NCC1|SCYA13|SCYL1 17 Cytokines

CCL14 6358 C-C motif chemokine ligand 14 CC-1|CC-3|CKB1|HCC-1|HCC-1(1-74)|HCC-1/HCC-3|HCC-3|MCIF|NCC-2|NCC2|SCYA14|SCYL2|SY14 17 Cytokines

CCL15-CCL14 348249 CCL15-CCL14 readthrough (NMD candidate) CCL15|HCC-2|LKN-1|MIP-5|MIP5|Mrp-2b|NCC-3|NCC3|SCYA15 17 Cytokines

CCL15 6359 C-C motif chemokine ligand 15 HCC-2|HMRP-2B|LKN-1|LKN1|MIP-1 delta|MIP-1D|MIP-5|MRP-2B|NCC-3|NCC3|SCYA15|SCYL3|SY15 17 Cytokines

CCL16 6360 C-C motif chemokine ligand 16 CKb12|HCC-4|ILINCK|LCC-1|LEC|LMC|Mtn-1|NCC-4|NCC4|SCYA16|SCYL4 17 Cytokines

CCL17 6361 C-C motif chemokine ligand 17 A-152E5.3|ABCD-2|SCYA17|TARC 16 Cytokines

CCL18 6362 C-C motif chemokine ligand 18 AMAC-1|AMAC1|CKb7|DC-CK1|DCCK1|MIP-4|PARC|SCYA18 17 Cytokines

CCL19 6363 C-C motif chemokine ligand 19 CKb11|ELC|MIP-3b|MIP3B|SCYA19 9 Cytokines

CCL2 6347 C-C motif chemokine ligand 2 GDCF-2|HC11|HSMCR30|MCAF|MCP-1|MCP1|SCYA2|SMC-CF 17 Cytokines

CCL20 6364 C-C motif chemokine ligand 20 CKb4|Exodus|LARC|MIP-3-alpha|MIP-3a|MIP3A|SCYA20|ST38 2 Cytokines

CCL21 6366 C-C motif chemokine ligand 21 6Ckine|CKb9|ECL|SCYA21|SLC|TCA4 9 Cytokines

CCL22 6367 C-C motif chemokine ligand 22 A-152E5.1|ABCD-1|DC/B-CK|MDC|SCYA22|STCP-1 16 Cytokines

CCL23 6368 C-C motif chemokine ligand 23 CK-BETA-8|CKb8|Ckb-8|Ckb-8-1|MIP-3|MIP3|MPIF-1|SCYA23|hmrp-2a 17 Cytokines

CCL24 6369 C-C motif chemokine ligand 24 Ckb-6|MPIF-2|MPIF2|SCYA24 7 Cytokines

CCL25 6370 C-C motif chemokine ligand 25 Ckb15|SCYA25|TECK 19 Cytokines

CCL26 10344 C-C motif chemokine ligand 26 IMAC|MIP-4a|MIP-4alpha|SCYA26|TSC-1 7 Cytokines

CCL27 10850 C-C motif chemokine ligand 27 ALP|CTACK|CTAK|ESKINE|ILC|PESKY|SCYA27 9 Cytokines

CCL28 56477 C-C motif chemokine ligand 28 CCK1|MEC|SCYA28 5 Cytokines

CCL3 6348 C-C motif chemokine ligand 3 G0S19-1|LD78ALPHA|MIP-1-alpha|MIP1A|SCYA3 17 Cytokines

CCL3L1 6349 C-C motif chemokine ligand 3 like 1 464.2|D17S1718|G0S19-2|LD78|LD78-beta(1-70)|LD78BETA|MIP1AP|SCYA3L|SCYA3L1 17 Cytokines

CCL3P1 390788 C-C motif chemokine ligand 3 pseudogene 1 CCL3L2|G0S19-3|LD78gamma|SCYA3L2 17 Cytokines

CCL3L3 414062 C-C motif chemokine ligand 3 like 3 464.2|D17S1718|G0S19-2|LD78|LD78BETA|SCYA3L|SCYA3L1 17 Cytokines

CCL4 6351 C-C motif chemokine ligand 4 ACT2|AT744.1|G-26|HC21|LAG-1|LAG1|MIP-1-beta|MIP1B|MIP1B1|SCYA2|SCYA4 17 Cytokines

CCL4L2 9560 C-C motif chemokine ligand 4 like 2 AT744.2|CCL4L|SCYA4L|SCYQ4L2 17 Cytokines

CCL4L1 388372 C-C motif chemokine ligand 4 like 1 AT744.2|CCL4L|LAG-1|LAG1|MIP-1-beta|SCYA4L|SCYA4L1|SCYA4L2 17 Cytokines

CCL5 6352 C-C motif chemokine ligand 5 D17S136E|RANTES|SCYA5|SIS-delta|SISd|TCP228|eoCP 17 Cytokines

CCL7 6354 C-C motif chemokine ligand 7 FIC|MARC|MCP-3|MCP3|NC28|SCYA6|SCYA7 17 Cytokines

CCL8 6355 C-C motif chemokine ligand 8 HC14|MCP-2|MCP2|SCYA10|SCYA8 17 Cytokines

CD320 51293 CD320 molecule 8D6|8D6A|TCBLR|TCN2R 19 Cytokines

CD40LG 959 CD40 ligand CD154|CD40L|HIGM1|IGM|IMD3|T-BAM|TNFSF5|TRAP|gp39|hCD40L X Cytokines

CD70 970 CD70 molecule CD27-L|CD27L|CD27LG|LPFS3|TNFSF7|TNLG8A 19 Cytokines

ADA2 51816 adenosine deaminase 2 ADGF|CECR1|IDGFL|PAN|SNEDS|VAIHS 22 Cytokines

CER1 9350 cerberus 1, DAN family BMP antagonist DAND4 9 Cytokines

CGA 1081 glycoprotein hormones, alpha polypeptide CG-ALPHA|FSHA|GPA1|GPHA1|GPHa|HCG|LHA|TSHA 6 Cytokines

CGB3 1082 chorionic gonadotropin subunit beta 3 CGB|CGB5|CGB7|CGB8|hCGB 19 Cytokines

CGB1 114335 chorionic gonadotropin subunit beta 1 - 19 Cytokines

CGB2 114336 chorionic gonadotropin subunit beta 2 - 19 Cytokines

CGB5 93659 chorionic gonadotropin subunit beta 5 CGB|HCG|hCGB 19 Cytokines

CGB7 94027 chorionic gonadotropin subunit beta 7 CG-beta-a|CGB6 19 Cytokines

CGB8 94115 chorionic gonadotropin subunit beta 8 - 19 Cytokines

CHGA 1113 chromogranin A CGA 14 Cytokines

CHGB 1114 chromogranin B SCG1 20 Cytokines

CKLF 51192 chemokine like factor C32|CKLF1|CKLF2|CKLF3|CKLF4|HSPC224|UCK-1 16 Cytokines

CLCF1 23529 cardiotrophin like cytokine factor 1 BSF-3|BSF3|CISS2|CLC|NNT-1|NNT1|NR6 11 Cytokines

CLEC11A 6320 C-type lectin domain containing 11A CLECSF3|LSLCL|P47|SCGF 19 Cytokines

CMA1 1215 chymase 1 CYH|MCT1|chymase 14 Cytokines

CMTM1 113540 CKLF like MARVEL transmembrane domain containing 1 CKLFH|CKLFH1|CKLFSF1 16 Cytokines

CMTM2 146225 CKLF like MARVEL transmembrane domain containing 2 CKLFSF2 16 Cytokines

CMTM3 123920 CKLF like MARVEL transmembrane domain containing 3 BNAS2|CKLFSF3 16 Cytokines

CMTM4 146223 CKLF like MARVEL transmembrane domain containing 4 CKLFSF4 16 Cytokines

CMTM5 116173 CKLF like MARVEL transmembrane domain containing 5 CKLFSF5 14 Cytokines

CMTM6 54918 CKLF like MARVEL transmembrane domain containing 6 CKLFSF6|PRO2219 3 Cytokines

CMTM7 112616 CKLF like MARVEL transmembrane domain containing 7 CKLFSF7 3 Cytokines

CMTM8 152189 CKLF like MARVEL transmembrane domain containing 8 CKLFSF8|CKLFSF8-V2 3 Cytokines

CNTF 1270 ciliary neurotrophic factor HCNTF 11 Cytokines

CORT 1325 cortistatin CST-14|CST-17|CST-29 1 Cytokines

CRH 1392 corticotropin releasing hormone CRF|CRH1 8 Cytokines

CSF1 1435 colony stimulating factor 1 CSF-1|MCSF 1 Cytokines

CSF2 1437 colony stimulating factor 2 CSF|GMCSF 5 Cytokines

CSF3 1440 colony stimulating factor 3 C17orf33|CSF3OS|GCSF 17 Cytokines

CSH1 1442 chorionic somatomammotropin hormone 1 CS-1|CSA|CSMT|GHB3|PL|hCS-1|hCS-A 17 Cytokines

CSH2 1443 chorionic somatomammotropin hormone 2 CS-2|CSB|GHB1|PL|hCS-B 17 Cytokines

CSHL1 1444 chorionic somatomammotropin hormone like 1 CS-5|CSHP1|CSL|GHB4|hCS-L 17 Cytokines

CSPG5 10675 chondroitin sulfate proteoglycan 5 NGC 3 Cytokines

CTF1 1489 cardiotrophin 1 CT-1|CT1 16 Cytokines

CCN2 1490 cellular communication network factor 2 CTGF|HCS24|IGFBP8|NOV2 6 Cytokines

CTSG 1511 cathepsin G CATG|CG 14 Cytokines

CX3CL1 6376 C-X3-C motif chemokine ligand 1 ABCD-3|C3Xkine|CXC3|CXC3C|NTN|NTT|SCYD1|fractalkine|neurotactin 16 Cytokines

CXCL1 2919 C-X-C motif chemokine ligand 1 FSP|GRO1|GROa|MGSA|MGSA-a|NAP-3|SCYB1 4 Cytokines

CXCL10 3627 C-X-C motif chemokine ligand 10 C7|IFI10|INP10|IP-10|SCYB10|crg-2|gIP-10|mob-1 4 Cytokines

CXCL11 6373 C-X-C motif chemokine ligand 11 H174|I-TAC|IP-9|IP9|SCYB11|SCYB9B|b-R1 4 Cytokines

CXCL12 6387 C-X-C motif chemokine ligand 12 IRH|PBSF|SCYB12|SDF1|TLSF|TPAR1 10 Cytokines

CXCL13 10563 C-X-C motif chemokine ligand 13 ANGIE|ANGIE2|BCA-1|BCA1|BLC|BLR1L|SCYB13 4 Cytokines

CXCL14 9547 C-X-C motif chemokine ligand 14 BMAC|BRAK|KEC|KS1|MIP-2g|MIP2G|NJAC|SCYB14 5 Cytokines

CXCL16 58191 C-X-C motif chemokine ligand 16 CXCLG16|SR-PSOX|SRPSOX 17 Cytokines

CXCL17 284340 C-X-C motif chemokine ligand 17 DMC|Dcip1|UNQ473|VCC-1|VCC1 19 Cytokines

CXCL2 2920 C-X-C motif chemokine ligand 2 CINC-2a|GRO2|GROb|MGSA-b|MIP-2a|MIP2|MIP2A|SCYB2 4 Cytokines

CXCL3 2921 C-X-C motif chemokine ligand 3 CINC-2b|GRO3|GROg|MIP-2b|MIP2B|SCYB3 4 Cytokines

CXCL5 6374 C-X-C motif chemokine ligand 5 ENA-78|SCYB5 4 Cytokines

CXCL6 6372 C-X-C motif chemokine ligand 6 CKA-3|GCP-2|GCP2|SCYB6 4 Cytokines

CXCL9 4283 C-X-C motif chemokine ligand 9 CMK|Humig|MIG|SCYB9|crg-10 4 Cytokines

CCN1 3491 cellular communication network factor 1 CYR61|GIG1|IGFBP10 1 Cytokines

DEFA1 1667 defensin alpha 1 DEF1|DEFA2|HNP-1|HP-1|HP1|MRS 8 Cytokines

DEFA3 1668 defensin alpha 3 DEF3|HNP-3|HNP3|HP-3|HP3 8 Cytokines

DEFA5 1670 defensin alpha 5 DEF5|HD-5 8 Cytokines

DEFB1 1672 defensin beta 1 BD1|DEFB-1|DEFB101|HBD1 8 Cytokines

DEFB103B 55894 defensin beta 103B BD-3|DEFB-3|DEFB103|DEFB3|HBD-3|HBD3|HBP-3|HBP3 8 Cytokines

DEFB104A 140596 defensin beta 104A BD-4|DEFB-4|DEFB104|DEFB4|hBD-4 8 Cytokines

DEFB4A 1673 defensin beta 4A BD-2|DEFB-2|DEFB102|DEFB2|DEFB4|HBD-2|SAP1 8 Cytokines

DKK1 22943 dickkopf WNT signaling pathway inhibitor 1 DKK-1|SK 10 Cytokines

EBI3 10148 Epstein-Barr virus induced 3 IL-27B|IL27B|IL35B 19 Cytokines

EDN1 1906 endothelin 1 ARCND3|ET1|HDLCQ7|PPET1|QME 6 Cytokines

EDN2 1907 endothelin 2 ET-2|ET2|PPET2 1 Cytokines

EDN3 1908 endothelin 3 ET-3|ET3|HSCR4|PPET3|WS4B 20 Cytokines

EGF 1950 epidermal growth factor HOMG4|URG 4 Cytokines

EPGN 255324 epithelial mitogen ALGV3072|EPG|PRO9904 4 Cytokines

EPO 2056 erythropoietin DBAL|ECYT5|EP|MVCD2 7 Cytokines

EREG 2069 epiregulin EPR|ER|Ep 4 Cytokines

ESM1 11082 endothelial cell specific molecule 1 endocan 5 Cytokines

FAM3B 54097 FAM3 metabolism regulating signaling molecule B 2-21|C21orf11|C21orf76|ORF9|PANDER|PRED44 21 Cytokines

FAM3C 10447 FAM3 metabolism regulating signaling molecule C GS3786|ILEI 7 Cytokines

FAM3D 131177 FAM3 metabolism regulating signaling molecule D EF7|OIT1 3 Cytokines

FASLG 356 Fas ligand ALPS1B|APT1LG1|APTL|CD178|CD95-L|CD95L|FASL|TNFSF6|TNLG1A 1 Cytokines

FGF1 2246 fibroblast growth factor 1 AFGF|ECGF|ECGF-beta|ECGFA|ECGFB|FGF-1|FGF-alpha|FGFA|GLIO703|HBGF-1|HBGF1 5 Cytokines

FGF10 2255 fibroblast growth factor 10 - 5 Cytokines

FGF11 2256 fibroblast growth factor 11 FGF-11|FHF-3|FHF3 17 Cytokines

FGF12 2257 fibroblast growth factor 12 EIEE47|FGF12B|FHF1 3 Cytokines

FGF13 2258 fibroblast growth factor 13 FGF-13|FGF2|FHF-2|FHF2|LINC00889 X Cytokines

FGF14 2259 fibroblast growth factor 14 FGF-14|FHF-4|FHF4|SCA27 13 Cytokines

FGF16 8823 fibroblast growth factor 16 FGF-16|MF4 X Cytokines

FGF17 8822 fibroblast growth factor 17 FGF-13|FGF-17|HH20 8 Cytokines

FGF18 8817 fibroblast growth factor 18 FGF-18|ZFGF5 5 Cytokines

FGF19 9965 fibroblast growth factor 19 - 11 Cytokines

FGF2 2247 fibroblast growth factor 2 BFGF|FGF-2|FGFB|HBGF-2 4 Cytokines

FGF20 26281 fibroblast growth factor 20 FGF-20|RHDA2 8 Cytokines

FGF21 26291 fibroblast growth factor 21 - 19 Cytokines

FGF22 27006 fibroblast growth factor 22 - 19 Cytokines

FGF23 8074 fibroblast growth factor 23 ADHR|FGFN|HFTC2|HPDR2|HYPF|PHPTC 12 Cytokines

FGF3 2248 fibroblast growth factor 3 HBGF-3|INT2 11 Cytokines

FGF4 2249 fibroblast growth factor 4 FGF-4|HBGF-4|HST|HST-1|HSTF-1|HSTF1|K-FGF|KFGF 11 Cytokines

FGF5 2250 fibroblast growth factor 5 HBGF-5|Smag-82|TCMGLY 4 Cytokines

FGF6 2251 fibroblast growth factor 6 HBGF-6|HST2 12 Cytokines

FGF7 2252 fibroblast growth factor 7 HBGF-7|KGF 15 Cytokines

FGF8 2253 fibroblast growth factor 8 AIGF|FGF-8|HBGF-8|HH6|KAL6 10 Cytokines

FGF9 2254 fibroblast growth factor 9 FGF-9|GAF|HBFG-9|HBGF-9|SYNS3 13 Cytokines

VEGFD 2277 vascular endothelial growth factor D FIGF|VEGF-D X Cytokines

FIGNL2 401720 fidgetin like 2 - 12 Cytokines

FLT3LG 2323 fms related receptor tyrosine kinase 3 ligand FL|FLG3L|FLT3L 19 Cytokines

FSHB 2488 follicle stimulating hormone subunit beta HH24 11 Cytokines

GAL 51083 galanin and GMAP prepropeptide ETL8|GAL-GMAP|GALN|GLNN|GMAP 11 Cytokines

GALP 85569 galanin like peptide - 19 Cytokines

GAST 2520 gastrin GAS 17 Cytokines

GCG 2641 glucagon GLP-1|GLP1|GLP2|GRPP 2 Cytokines

GDF1 2657 growth differentiation factor 1 CERS1|CHTD6|DORV|DTGA3|LAG1|LASS1|RAI|UOG1 19 Cytokines

GDF10 2662 growth differentiation factor 10 BIP|BMP-3b|BMP3B 10 Cytokines

GDF11 10220 growth differentiation factor 11 BMP-11|BMP11 12 Cytokines

GDF15 9518 growth differentiation factor 15 GDF-15|MIC-1|MIC1|NAG-1|PDF|PLAB|PTGFB 19 Cytokines

GDF2 2658 growth differentiation factor 2 BMP-9|BMP9|HHT5 10 Cytokines

GDF3 9573 growth differentiation factor 3 KFS3|MCOP7|MCOPCB6 12 Cytokines

GDF5 8200 growth differentiation factor 5 BDA1C|BMP-14|BMP14|CDMP1|DUPANS|LAP-4|LAP4|OS5|SYM1B|SYNS2 20 Cytokines

GDF6 392255 growth differentiation factor 6 BMP-13|BMP13|CDMP2|KFM|KFS|KFS1|KFSL|SGM1|SYNS4 8 Cytokines

GDF7 151449 growth differentiation factor 7 BMP12 2 Cytokines

GDF9 2661 growth differentiation factor 9 POF14 5 Cytokines

GDNF 2668 glial cell derived neurotrophic factor ATF|ATF1|ATF2|HFB1-GDNF|HSCR3 5 Cytokines

GH1 2688 growth hormone 1 GH|GH-N|GHB5|GHN|IGHD1A|IGHD1B|IGHD2|hGH-N 17 Cytokines

GH2 2689 growth hormone 2 GH-V|GHB2|GHL|GHV|hGH-V 17 Cytokines

GHRH 2691 growth hormone releasing hormone GHRF|GRF|INN 20 Cytokines

GHRL 51738 ghrelin and obestatin prepropeptide MTLRP 3 Cytokines

GIP 2695 gastric inhibitory polypeptide - 17 Cytokines

GKN1 56287 gastrokine 1 AMP18|BRICD1|CA11|FOV|foveolin 2 Cytokines

GMFB 2764 glia maturation factor beta GMF 14 Cytokines

GMFG 9535 glia maturation factor gamma GMF-GAMMA 19 Cytokines

GNRH1 2796 gonadotropin releasing hormone 1 GNRH|GRH|LHRH|LNRH 8 Cytokines

GNRH2 2797 gonadotropin releasing hormone 2 GnRH-II|LH-RHII 20 Cytokines

GPHA2 170589 glycoprotein hormone subunit alpha 2 A2|GPA2|ZSIG51 11 Cytokines

GPHB5 122876 glycoprotein hormone subunit beta 5 B5|GPB5|ZLUT1 14 Cytokines

GPI 2821 glucose-6-phosphate isomerase AMF|GNPI|NLK|PGI|PHI|SA-36|SA36 19 Cytokines

GREM1 26585 gremlin 1, DAN family BMP antagonist C15DUPq|CKTSF1B1|CRAC1|CRCS4|DAND2|DRM|DUP15q|GREMLIN|HMPS|HMPS1|IHG-2|MPSH|PIG2 15 Cytokines

GREM2 64388 gremlin 2, DAN family BMP antagonist CKTSF1B2|DAND3|PRDC|STHAG9 1 Cytokines

GRN 2896 granulin precursor CLN11|GEP|GP88|PCDGF|PEPI|PGRN 17 Cytokines

GRP 2922 gastrin releasing peptide BN|GRP-10|preproGRP|proGRP 18 Cytokines

GUCA2A 2980 guanylate cyclase activator 2A GCAP-I|GUCA2|STARA 1 Cytokines

HAMP 57817 hepcidin antimicrobial peptide HEPC|HFE2B|LEAP1|PLTR 19 Cytokines

HBEGF 1839 heparin binding EGF like growth factor DTR|DTS|DTSF|HEGFL 5 Cytokines

HDGF 3068 heparin binding growth factor HMG1L2 1 Cytokines

HDGFL3 50810 HDGF like 3 CGI-142|HDGF-2|HDGF2|HDGFRP3|HRP-3 15 Cytokines

HGF 3082 hepatocyte growth factor DFNB39|F-TCF|HGFB|HPTA|SF 7 Cytokines

HTN3 3347 histatin 3 HIS2|HTN2|HTN5|PB 4 Cytokines

IAPP 3375 islet amyloid polypeptide DAP|IAP 12 Cytokines

IFNA1 3439 interferon alpha 1 IFL|IFN|IFN-ALPHA|IFN-alphaD|IFNA13|IFNA@|leIF D 9 Cytokines

IFNA10 3446 interferon alpha 10 IFN-alphaC 9 Cytokines

IFNA13 3447 interferon alpha 13 - 9 Cytokines

IFNA14 3448 interferon alpha 14 IFN-alphaH|LEIF2H 9 Cytokines

IFNA16 3449 interferon alpha 16 IFN-alpha-16|IFN-alphaO 9 Cytokines

IFNA17 3451 interferon alpha 17 IFN-alphaI|IFNA|INFA|LEIF2C1 9 Cytokines

IFNA2 3440 interferon alpha 2 IFN-alpha-2|IFN-alphaA|IFNA|IFNA2B|leIF A 9 Cytokines

IFNA21 3452 interferon alpha 21 IFN-alphaI|LeIF F|leIF-F 9 Cytokines

IFNA4 3441 interferon alpha 4 IFN-alpha4a|INFA4 9 Cytokines

IFNA5 3442 interferon alpha 5 IFN-alpha-5|IFN-alphaG|INA5|INFA5|leIF G 9 Cytokines

IFNA6 3443 interferon alpha 6 IFN-alphaK 9 Cytokines

IFNA7 3444 interferon alpha 7 IFN-alphaJ|IFNA-J 9 Cytokines

IFNA8 3445 interferon alpha 8 IFN-alphaB 9 Cytokines

IFNB1 3456 interferon beta 1 IFB|IFF|IFN-beta|IFNB 9 Cytokines

IFNE 338376 interferon epsilon IFN-E|IFNE1|IFNT1|INFE1|PRO655 9 Cytokines

IFNG 3458 interferon gamma IFG|IFI 12 Cytokines

IFNK 56832 interferon kappa IFNT1|INFE1 9 Cytokines

IFNW1 3467 interferon omega 1 - 9 Cytokines

IGF1 3479 insulin like growth factor 1 IGF|IGF-I|IGFI|MGF 12 Cytokines

IGF2 3481 insulin like growth factor 2 C11orf43|GRDF|IGF-II|PP9974 11 Cytokines

IL10 3586 interleukin 10 CSIF|GVHDS|IL-10|IL10A|TGIF 1 Cytokines

IL11 3589 interleukin 11 AGIF|IL-11 19 Cytokines

IL12A 3592 interleukin 12A CLMF|IL-12A|NFSK|NKSF1|P35 3 Cytokines

IL12B 3593 interleukin 12B CLMF|CLMF2|IL-12B|IMD28|IMD29|NKSF|NKSF2 5 Cytokines

IL13 3596 interleukin 13 IL-13|P600 5 Cytokines

IL15 3600 interleukin 15 IL-15 4 Cytokines

IL16 3603 interleukin 16 LCF|NIL16|PRIL16|prIL-16 15 Cytokines

IL17A 3605 interleukin 17A CTLA-8|CTLA8|IL-17|IL-17A|IL17 6 Cytokines

IL17B 27190 interleukin 17B IL-17B|IL-20|NIRF|ZCYTO7 5 Cytokines

IL17C 27189 interleukin 17C CX2|IL-17C 16 Cytokines

IL17D 53342 interleukin 17D IL-17D 13 Cytokines

IL17F 112744 interleukin 17F CANDF6|IL-17F|ML-1|ML1 6 Cytokines

IL18 3606 interleukin 18 IGIF|IL-18|IL-1g|IL1F4 11 Cytokines

IL19 29949 interleukin 19 IL-10C|MDA1|NG.1|ZMDA1 1 Cytokines

IL1A 3552 interleukin 1 alpha IL-1 alpha|IL-1A|IL1|IL1-ALPHA|IL1F1 2 Cytokines

IL1B 3553 interleukin 1 beta IL-1|IL1-BETA|IL1F2|IL1beta 2 Cytokines

IL1F10 84639 interleukin 1 family member 10 FIL1-theta|FKSG75|IL-1HY2|IL-38|IL1-theta|IL1HY2 2 Cytokines

IL36RN 26525 interleukin 36 receptor antagonist FIL1|FIL1(DELTA)|FIL1D|IL-36Ra|IL1F5|IL1HY1|IL1L1|IL1RP3|IL36RA|PSORP|PSORS14 2 Cytokines

IL36A 27179 interleukin 36 alpha FIL1|FIL1(EPSILON)|FIL1E|IL-1F6|IL1(EPSILON)|IL1F6 2 Cytokines

IL37 27178 interleukin 37 FIL1|FIL1(ZETA)|FIL1Z|IL-1F7|IL-1H|IL-1H4|IL-1RP1|IL-37|IL1F7|IL1H4|IL1RP1 2 Cytokines

IL36B 27177 interleukin 36 beta FIL1|FIL1-(ETA)|FIL1H|FILI-(ETA)|IL-1F8|IL-1H2|IL1-ETA|IL1F8|IL1H2 2 Cytokines

IL36G 56300 interleukin 36 gamma IL-1F9|IL-1H1|IL-1RP2|IL1E|IL1F9|IL1H1|IL1RP2 2 Cytokines

IL1RN 3557 interleukin 1 receptor antagonist DIRA|ICIL-1RA|IL-1RN|IL-1ra|IL-1ra3|IL1F3|IL1RA|IRAP|MVCD4 2 Cytokines

IL2 3558 interleukin 2 IL-2|TCGF|lymphokine 4 Cytokines

IL20 50604 interleukin 20 IL-20|IL10D|ZCYTO10 1 Cytokines

IL21 59067 interleukin 21 CVID11|IL-21|Za11 4 Cytokines

IL22 50616 interleukin 22 IL-21|IL-22|IL-D110|IL-TIF|ILTIF|TIFIL-23|TIFa|zcyto18 12 Cytokines

IL23A 51561 interleukin 23 subunit alpha IL-23|IL-23A|IL23P19|P19|SGRF 12 Cytokines

IL24 11009 interleukin 24 C49A|FISP|IL10B|MDA7|MOB5|ST16 1 Cytokines

IL25 64806 interleukin 25 IL17E 14 Cytokines

IL26 55801 interleukin 26 AK155|IL-26 12 Cytokines

IL27 246778 interleukin 27 IL-27|IL-27A|IL27A|IL27p28|IL30|p28 16 Cytokines

IFNL2 282616 interferon lambda 2 IL-28A|IL28A 19 Cytokines

IFNL3 282617 interferon lambda 3 IFN-lambda-3|IFN-lambda-4|IL-28B|IL-28C|IL28B|IL28C 19 Cytokines

IFNL1 282618 interferon lambda 1 IL-29|IL29 19 Cytokines

IL3 3562 interleukin 3 IL-3|MCGF|MULTI-CSF 5 Cytokines

IL31 386653 interleukin 31 IL-31 12 Cytokines

IL32 9235 interleukin 32 IL-32alpha|IL-32beta|IL-32delta|IL-32gamma|NK4|TAIF|TAIFa|TAIFb|TAIFc|TAIFd 16 Cytokines

IL33 90865 interleukin 33 C9orf26|DVS27|IL1F11|NF-HEV|NFEHEV 9 Cytokines

IL34 146433 interleukin 34 C16orf77|IL-34 16 Cytokines

IL4 3565 interleukin 4 BCGF-1|BCGF1|BSF-1|BSF1|IL-4 5 Cytokines

IL5 3567 interleukin 5 EDF|IL-5|TRF 5 Cytokines

IL6 3569 interleukin 6 BSF-2|BSF2|CDF|HGF|HSF|IFN-beta-2|IFNB2|IL-6 7 Cytokines

IL6ST 3572 interleukin 6 signal transducer CD130|CDW130|GP130|HIES4|IL-6RB|sGP130 5 Cytokines

IL7 3574 interleukin 7 IL-7 8 Cytokines

CXCL8 3576 C-X-C motif chemokine ligand 8 GCP-1|GCP1|IL8|LECT|LUCT|LYNAP|MDNCF|MONAP|NAF|NAP-1|NAP1|SCYB8 4 Cytokines

IL9 3578 interleukin 9 HP40|IL-9|P40 5 Cytokines

INHA 3623 inhibin subunit alpha - 2 Cytokines

INHBA 3624 inhibin subunit beta A EDF|FRP 7 Cytokines

INHBB 3625 inhibin subunit beta B - 2 Cytokines

INHBC 3626 inhibin subunit beta C IHBC 12 Cytokines

INHBE 83729 inhibin subunit beta E - 12 Cytokines

INS 3630 insulin IDDM|IDDM1|IDDM2|ILPR|IRDN|MODY10|PNDM4 11 Cytokines

INS-IGF2 723961 INS-IGF2 readthrough INSIGF 11 Cytokines

INSL3 3640 insulin like 3 RLF|RLNL|ley-I-L 19 Cytokines

INSL4 3641 insulin like 4 EPIL|PLACENTIN 9 Cytokines

INSL5 10022 insulin like 5 PRO182|UNQ156 1 Cytokines

INSL6 11172 insulin like 6 RIF1 9 Cytokines

JAG1 182 jagged canonical Notch ligand 1 AGS|AGS1|AHD|AWS|CD339|DCHE|HJ1|JAGL1 20 Cytokines

JAG2 3714 jagged canonical Notch ligand 2 HJ2|SER2 14 Cytokines

FGF7P6 387628 fibroblast growth factor 7 pseudogene 6 KGFLP1 9 Cytokines

FGF7P3 654466 fibroblast growth factor 7 pseudogene 3 KGFLP2 9 Cytokines

KITLG 4254 KIT ligand DCUA|DFNA69|FPH2|FPHH|KL-1|Kitl|MGF|SCF|SF|SHEP7|SLF 12 Cytokines

KL 9365 klotho HFTC3 13 Cytokines

LACRT 90070 lacritin - 12 Cytokines

LECT2 3950 leukocyte cell derived chemotaxin 2 chm-II|chm2 5 Cytokines

LEFTY1 10637 left-right determination factor 1 LEFTB|LEFTYB 1 Cytokines

LEFTY2 7044 left-right determination factor 2 EBAF|LEFTA|LEFTYA|TGFB4 1 Cytokines

LEP 3952 leptin LEPD|OB|OBS 7 Cytokines

LHB 3972 luteinizing hormone subunit beta CGB4|HH23|LSH-B|LSH-beta 19 Cytokines

LIF 3976 LIF interleukin 6 family cytokine CDF|DIA|HILDA|MLPLI 22 Cytokines

LRSAM1 90678 leucine rich repeat and sterile alpha motif containing 1 CMT2P|RIFLE|TAL 9 Cytokines

LTA 4049 lymphotoxin alpha LT|TNFB|TNFSF1|TNLG1E 6 Cytokines

LTB 4050 lymphotoxin beta TNFC|TNFSF3|TNLG1C|p33 6 Cytokines

LTBP1 4052 latent transforming growth factor beta binding protein 1 - 2 Cytokines

LTBP2 4053 latent transforming growth factor beta binding protein 2 C14orf141|GLC3D|LTBP3|MSPKA|MSTP031|WMS3 14 Cytokines

LTBP3 4054 latent transforming growth factor beta binding protein 3 DASS|GPHYSD3|LTBP-3|LTBP2|STHAG6|pp6425 11 Cytokines

LTBP4 8425 latent transforming growth factor beta binding protein 4 ARCL1C|LTBP-4|LTBP4L|LTBP4S 19 Cytokines

MDK 4192 midkine ARAP|MK|NEGF2 11 Cytokines

MIA 8190 MIA SH3 domain containing CD-RAP 19 Cytokines

MIF 4282 macrophage migration inhibitory factor GIF|GLIF|MMIF 22 Cytokines

MLN 4295 motilin - 6 Cytokines

MSTN 2660 myostatin GDF8|MSLHP 2 Cytokines

NAMPT 10135 nicotinamide phosphoribosyltransferase 1110035O14Rik|PBEF|PBEF1|VF|VISFATIN 7 Cytokines

NDP 4693 norrin cystine knot growth factor NDP EVR2|FEVR|ND X Cytokines

NENF 29937 neudesin neurotrophic factor CIR2|SCIRP10|SPUF 1 Cytokines

NGF 4803 nerve growth factor Beta-NGF|HSAN5|NGFB 1 Cytokines

NMB 4828 neuromedin B - 15 Cytokines

NODAL 4838 nodal growth differentiation factor HTX5 10 Cytokines

CCN3 4856 cellular communication network factor 3 IBP-9|IGFBP-9|IGFBP9|NOV|NOVh 8 Cytokines

NPFF 8620 neuropeptide FF-amide peptide precursor FMRFAL 12 Cytokines

NPPA 4878 natriuretic peptide A ANF|ANP|ATFB6|ATRST2|CDD|CDD-ANF|CDP|PND 1 Cytokines

NPPB 4879 natriuretic peptide B BNP 1 Cytokines

NPPC 4880 natriuretic peptide C CNP|CNP2 2 Cytokines

NPY 4852 neuropeptide Y PYY4 7 Cytokines

NRG1 3084 neuregulin 1 ARIA|GGF|GGF2|HGL|HRG|HRG1|HRGA|MST131|MSTP131|NDF|NRG1-IT2|SMDF 8 Cytokines

NRG2 9542 neuregulin 2 DON1|HRG2|NTAK 5 Cytokines

NRG3 10718 neuregulin 3 HRG3|pro-NRG3 10 Cytokines

NRG4 145957 neuregulin 4 HRG4 15 Cytokines

NRTN 4902 neurturin NTN 19 Cytokines

NTF3 4908 neurotrophin 3 HDNF|NGF-2|NGF2|NT-3|NT3 12 Cytokines

NTF4 4909 neurotrophin 4 GLC10|GLC1O|NT-4|NT-4/5|NT-5|NT4|NT5|NTF5 19 Cytokines

NTS 4922 neurotensin NMN-125|NN|NT|NT/N|NTS1 12 Cytokines

NUDT6 11162 nudix hydrolase 6 ASFGF2|FGF-AS|FGF2AS|GFG-1|GFG1 4 Cytokines

OGN 4969 osteoglycin OG|OIF|SLRR3A 9 Cytokines

OSGIN1 29948 oxidative stress induced growth inhibitor 1 BDGI|OKL38 16 Cytokines

OSM 5008 oncostatin M - 22 Cytokines

OSTN 344901 osteocrin MUSCLIN 3 Cytokines

OXT 5020 oxytocin/neurophysin I prepropeptide OT|OT-NPI|OXT-NPI 20 Cytokines

ENDOU 8909 endonuclease, poly(U) specific P11|PP11|PRSS26 12 Cytokines

PDGFA 5154 platelet derived growth factor subunit A PDGF-A|PDGF1 7 Cytokines

PDGFB 5155 platelet derived growth factor subunit B IBGC5|PDGF-2|PDGF2|SIS|SSV|c-sis 22 Cytokines

PDGFC 56034 platelet derived growth factor C FALLOTEIN|SCDGF 4 Cytokines

PDGFD 80310 platelet derived growth factor D IEGF|MSTP036|SCDGF-B|SCDGFB 11 Cytokines

PDGFRA 5156 platelet derived growth factor receptor alpha CD140A|PDGFR-2|PDGFR2 4 Cytokines

PDGFRB 5159 platelet derived growth factor receptor beta CD140B|IBGC4|IMF1|JTK12|KOGS|PDGFR|PDGFR-1|PDGFR1|PENTT 5 Cytokines

PDGFRL 5157 platelet derived growth factor receptor like PDGRL|PRLTS 8 Cytokines

PDYN 5173 prodynorphin ADCA|PENKB|SCA23 20 Cytokines

PENK 5179 proenkephalin PE|PENK-A 8 Cytokines

PF4 5196 platelet factor 4 CXCL4|PF-4|SCYB4 4 Cytokines

PF4V1 5197 platelet factor 4 variant 1 CXCL4L1|CXCL4V1|PF4-ALT|PF4A|SCYB4V1 4 Cytokines

PGF 5228 placental growth factor D12S1900|PGFL|PIGF|PLGF|PlGF-2|SHGC-10760 14 Cytokines

PLAU 5328 plasminogen activator, urokinase ATF|BDPLT5|QPD|UPA|URK|u-PA 10 Cytokines

PMCH 5367 pro-melanin concentrating hormone MCH|ppMCH 12 Cytokines

PNOC 5368 prepronociceptin N/OFQ|NOP|OFQ|PPNOC|ppN/OFQ 8 Cytokines

POMC 5443 proopiomelanocortin ACTH|CLIP|LPH|MSH|NPP|OBAIRH|POC 2 Cytokines

PPBP 5473 pro-platelet basic protein B-TG1|Beta-TG|CTAP-III|CTAP3|CTAPIII|CXCL7|LA-PF4|LDGF|MDGF|NAP-2|PBP|SCYB7|TC1|TC2|TGB|TGB1|THBGB|THBGB1 4 Cytokines

PPBPP1 728045 pro-platelet basic protein pseudogene 1 PPBPL1|TGB2 4 Cytokines

PPBPP2 10895 pro-platelet basic protein pseudogene 2 PPBPL2|SPBPBP 4 Cytokines

PPY 5539 pancreatic polypeptide PNP|PP 17 Cytokines

PRL 5617 prolactin GHA1 6 Cytokines

PRLH 51052 prolactin releasing hormone PRH|PRRP 2 Cytokines

PROK1 84432 prokineticin 1 EGVEGF|PK1|PRK1 1 Cytokines

PROK2 60675 prokineticin 2 BV8|HH4|KAL4|MIT1|PK2 3 Cytokines

PSPN 5623 persephin PSP 19 Cytokines

PTH 5741 parathyroid hormone FIH1|PTH1 11 Cytokines

PTH2 113091 parathyroid hormone 2 TIP39 19 Cytokines

PTHLH 5744 parathyroid hormone like hormone BDE2|HHM|PLP|PTHR|PTHRP 12 Cytokines

PTN 5764 pleiotrophin HARP|HB-GAM|HBBM|HBGF-8|HBGF8|HBNF|HBNF-1|NEGF1|OSF-1 7 Cytokines

PYY 5697 peptide YY PYY-I|PYY1 17 Cytokines

QRFP 347148 pyroglutamylated RFamide peptide 26RFa|P518 9 Cytokines

RABEP1 9135 rabaptin, RAB GTPase binding effector protein 1 RAB5EP|RABPT5 17 Cytokines

RABEP2 79874 rabaptin, RAB GTPase binding effector protein 2 FRA 16 Cytokines

REG1A 5967 regenerating family member 1 alpha ICRF|P19|PSP|PSPS|PSPS1|PTP|REG 2 Cytokines

RETN 56729 resistin ADSF|FIZZ3|RETN1|RSTN|XCP1 19 Cytokines

RETNLB 84666 resistin like beta FIZZ1|FIZZ2|HXCP2|RELM-beta|RELMb|RELMbeta|XCP2 3 Cytokines

RLN1 6013 relaxin 1 H1|H1RLX|RLXH1|bA12D24.3.1|bA12D24.3.2 9 Cytokines

RLN2 6019 relaxin 2 H2|H2-RLX|RLXH2|bA12D24.1.1|bA12D24.1.2 9 Cytokines

RLN3 117579 relaxin 3 H3|RXN3|ZINS4|insl7 19 Cytokines

RNASE2 6036 ribonuclease A family member 2 EDN|RAF3|RNS2 14 Cytokines

S100A6 6277 S100 calcium binding protein A6 2A9|5B10|CABP|CACY|PRA|S10A6 1 Cytokines

SAA1 6288 serum amyloid A1 PIG4|SAA|SAA2|TP53I4 11 Cytokines

SAA2 6289 serum amyloid A2 SAA|SAA1 11 Cytokines

SBDS 51119 SBDS ribosome maturation factor CGI-97|SDS|SWDS 7 Cytokines

SCG2 7857 secretogranin II CHGC|EM66|SN|SgII 2 Cytokines

SCGB3A1 92304 secretoglobin family 3A member 1 HIN-1|HIN1|LU105|PnSP-2|UGRP2 5 Cytokines

SCT 6343 secretin - 11 Cytokines

AIMP1 9255 aminoacyl tRNA synthetase complex interacting multifunctional protein 1 EMAP2|EMAPII|HLD3|SCYE1|p43 4 Cytokines

SECTM1 6398 secreted and transmembrane 1 K12|SECTM 17 Cytokines

SEMA3A 10371 semaphorin 3A COLL1|HH16|Hsema-I|Hsema-III|SEMA1|SEMAD|SEMAIII|SEMAL|SemD|coll-1 7 Cytokines

SEMA3B 7869 semaphorin 3B LUCA-1|SEMA5|SEMAA|SemA|semaV 3 Cytokines

SEMA3C 10512 semaphorin 3C SEMAE|SemE 7 Cytokines

SEMA3D 223117 semaphorin 3D Sema-Z2|coll-2 7 Cytokines

SEMA3E 9723 semaphorin 3E M-SEMAH|M-SemaK|SEMAH|coll-5 7 Cytokines

SEMA3F 6405 semaphorin 3F SEMA-IV|SEMA4|SEMAK 3 Cytokines

SEMA3G 56920 semaphorin 3G sem2 3 Cytokines

SEMA4A 64218 semaphorin 4A CORD10|RP35|SEMAB|SEMB 1 Cytokines

SEMA4B 10509 semaphorin 4B SEMAC|SemC 15 Cytokines

SEMA4C 54910 semaphorin 4C M-SEMA-F|SEMACL1|SEMAF|SEMAI 2 Cytokines

SEMA4D 10507 semaphorin 4D A8|BB18|C9orf164|CD100|COLL4|GR3|M-sema-G|SEMAJ|coll-4 9 Cytokines

SEMA4F 10505 ssemaphorin 4F M-SEMA|PRO2353|S4F|SEMAM|SEMAW|m-Sema-M 2 Cytokines

SEMA4G 57715 semaphorin 4G - 10 Cytokines

SEMA5A 9037 semaphorin 5A SEMAF|semF 5 Cytokines

SEMA5B 54437 semaphorin 5B SEMAG|SemG 3 Cytokines

SEMA6A 57556 semaphorin 6A HT018|SEMA|SEMA6A1|SEMAQ|VIA 5 Cytokines

SEMA6B 10501 semaphorin 6B EPM11|SEM-SEMA-Y|SEMA-VIB|SEMAN|semaZ 19 Cytokines

SEMA6C 10500 semaphorin 6C SEMAY|m-SemaY|m-SemaY2 1 Cytokines

SEMA6D 80031 semaphorin 6D - 15 Cytokines

SEMA7A 8482 semaphorin 7A (John Milton Hagen blood group) CD108|CDw108|H-SEMA-K1|H-Sema-L|JMH|SEMAK1|SEMAL 15 Cytokines

SLIT1 6585 slit guidance ligand 1 MEGF4|SLIL1|SLIT-1|SLIT3 10 Cytokines

SLIT2 9353 slit guidance ligand 2 SLIL3|Slit-2 4 Cytokines

SLURP1 57152 secreted LY6/PLAUR domain containing 1 ANUP|ARS|ArsB|LY6-MT|LY6LS|MDM 8 Cytokines

SPP1 6696 secreted phosphoprotein 1 BNSP|BSPI|ETA-1|OPN 4 Cytokines

SST 6750 somatostatin SMST 3 Cytokines

STC1 6781 stanniocalcin 1 STC 8 Cytokines

STC2 8614 stanniocalcin 2 STC-2|STCRP 5 Cytokines

TAC1 6863 tachykinin precursor 1 Hs.2563|NK2|NKNA|NPK|TAC2 7 Cytokines

TDGF1 6997 teratocarcinoma-derived growth factor 1 CR|CR-1|CRGF|CRIPTO 3 Cytokines

TDGF1P3 6998 teratocarcinoma-derived growth factor 1 pseudogene 3 CR-3|CRIPTO|CRIPTO-3|CRIPTO3|TDGF1|TDGF2|TDGF3 X Cytokines

TG 7038 thyroglobulin AITD3|TGN 8 Cytokines

TGFA 7039 transforming growth factor alpha TFGA 2 Cytokines

TGFB1 7040 transforming growth factor beta 1 CED|DPD1|IBDIMDE|LAP|TGF-beta1|TGFB|TGFbeta 19 Cytokines

TGFB2 7042 transforming growth factor beta 2 G-TSF|LDS4|TGF-beta2 1 Cytokines

TGFB3 7043 transforming growth factor beta 3 ARVD|ARVD1|LDS5|RNHF|TGF-beta3 14 Cytokines

THPO 7066 thrombopoietin MGDF|MKCSF|ML|MPLLG|THCYT1|TPO 3 Cytokines

TNC 3371 tenascin C 150-225|DFNA56|GMEM|GP|HXB|JI|TN|TN-C 9 Cytokines

TNF 7124 tumor necrosis factor DIF|TNF-alpha|TNFA|TNFSF2|TNLG1F 6 Cytokines

TNFRSF11B 4982 TNF receptor superfamily member 11b OCIF|OPG|PDB5|TR1 8 Cytokines

TNFSF10 8743 TNF superfamily member 10 APO2L|Apo-2L|CD253|TL2|TNLG6A|TRAIL 3 Cytokines

TNFSF11 8600 TNF superfamily member 11 CD254|ODF|OPGL|OPTB2|RANKL|TNLG6B|TRANCE|hRANKL2|sOdf 13 Cytokines

TNFSF12 8742 TNF superfamily member 12 APO3L|DR3LG|TNLG4A|TWEAK 17 Cytokines

TNFSF13 8741 TNF superfamily member 13 APRIL|CD256|TALL-2|TALL2|TNLG7B|TRDL-1|UNQ383/PRO715|ZTNF2 17 Cytokines

TNFSF13B 10673 TNF superfamily member 13b BAFF|BLYS|CD257|DTL|TALL-1|TALL1|THANK|TNFSF20|TNLG7A|ZTNF4 13 Cytokines

TNFSF14 8740 TNF superfamily member 14 CD258|HVEML|LIGHT|LTg 19 Cytokines

TNFSF15 9966 TNF superfamily member 15 TL1|TL1A|TNLG1B|VEGI|VEGI192A 9 Cytokines

TNFSF18 8995 TNF superfamily member 18 AITRL|GITRL|TL6|TNLG2A|hGITRL 1 Cytokines

TNFSF4 7292 TNF superfamily member 4 CD134L|CD252|GP34|OX-40L|OX4OL|TNLG2B|TXGP1 1 Cytokines

TNFSF8 944 TNF superfamily member 8 CD153|CD30L|CD30LG|TNLG3A 9 Cytokines

TNFSF9 8744 TNF superfamily member 9 4-1BB-L|CD137L|TNLG5A 19 Cytokines

TOR2A 27433 torsin family 2 member A TORP1 9 Cytokines

TRH 7200 thyrotropin releasing hormone Pro-TRH|TRF 3 Cytokines

TSHB 7252 thyroid stimulating hormone subunit beta TSH-B|TSH-BETA 1 Cytokines

TSLP 85480 thymic stromal lymphopoietin - 5 Cytokines

TXLNA 200081 taxilin alpha IL14|TXLN 1 Cytokines

TYMP 1890 thymidine phosphorylase ECGF|ECGF1|MEDPS1|MNGIE|MTDPS1|PDECGF|TP|hPD-ECGF 22 Cytokines

UCN 7349 urocortin UI|UROC 2 Cytokines

UCN2 90226 urocortin 2 SRP|UCN-II|UCNI|UR|URP 3 Cytokines

UCN3 114131 urocortin 3 SCP|SPC|UCNIII 10 Cytokines

UTS2 10911 urotensin 2 PRO1068|U-II|UCN2|UII 1 Cytokines

UTS2B 257313 urotensin 2B U2B|URP|UTS2D 3 Cytokines

VEGFA 7422 vascular endothelial growth factor A MVCD1|VEGF|VPF 6 Cytokines

VEGFB 7423 vascular endothelial growth factor B VEGFL|VRF 11 Cytokines

VEGFC 7424 vascular endothelial growth factor C Flt4-L|LMPH1D|LMPHM4|VRP 4 Cytokines

VGF 7425 VGF nerve growth factor inducible SCG7|SgVII 7 Cytokines

VIP 7432 vasoactive intestinal peptide PHM27 6 Cytokines

XCL1 6375 X-C motif chemokine ligand 1 ATAC|LPTN|LTN|SCM-1|SCM-1a|SCM1|SCM1A|SCYC1 1 Cytokines

XCL2 6846 X-C motif chemokine ligand 2 SCM-1b|SCM1B|SCYC2 1 Cytokines

ACVR1B 91 activin A receptor type 1B ACTRIB|ACVRLK4|ALK4|SKR2 12 Cytokine_Receptors

ACVR1C 130399 activin A receptor type 1C ACVRLK7|ALK7 2 Cytokine_Receptors

ACVR2A 92 activin A receptor type 2A ACTRII|ACVR2 2 Cytokine_Receptors

ACVR2B 93 activin A receptor type 2B ACTRIIB|ActR-IIB|HTX4 3 Cytokine_Receptors

ACVRL1 94 activin A receptor like type 1 ACVRLK1|ALK-1|ALK1|HHT|HHT2|ORW2|SKR3|TSR-I 12 Cytokine_Receptors

ADCYAP1R1 117 ADCYAP receptor type I PAC1|PAC1R|PACAPR|PACAPRI 7 Cytokine_Receptors

ADIPOR1 51094 adiponectin receptor 1 ACDCR1|CGI-45|CGI45|PAQR1|TESBP1A 1 Cytokine_Receptors

ADIPOR2 79602 adiponectin receptor 2 ACDCR2|PAQR2 12 Cytokine_Receptors

ADRB1 153 adrenoceptor beta 1 ADRB1R|B1AR|BETA1AR|FNSS2|RHR 10 Cytokine_Receptors

ADRB2 154 adrenoceptor beta 2 ADRB2R|ADRBR|B2AR|BAR|BETA2AR 5 Cytokine_Receptors

AGTR1 185 angiotensin II receptor type 1 AG2S|AGTR1B|AT1|AT1AR|AT1B|AT1BR|AT1R|AT2R1|HAT1R 3 Cytokine_Receptors

AGTR2 186 angiotensin II receptor type 2 AT2|ATGR2|MRX88 X Cytokine_Receptors

AMHR2 269 anti-Mullerian hormone receptor type 2 AMHR|MISR2|MISRII|MRII 12 Cytokine_Receptors

ANGPT1 284 angiopoietin 1 AGP1|AGPT|ANG1 8 Cytokine_Receptors

ANGPT4 51378 angiopoietin 4 ANG3|ANG4 20 Cytokine_Receptors

ANGPTL1 9068 angiopoietin like 1 ANG3|ANGPT3|ARP1|AngY|UNQ162|dJ595C2.2 1 Cytokine_Receptors

ANGPTL2 23452 angiopoietin like 2 ARP2|HARP 9 Cytokine_Receptors

ANGPTL3 27329 angiopoietin like 3 ANG-5|ANGPT5|ANL3|FHBL2 1 Cytokine_Receptors

ANGPTL4 51129 angiopoietin like 4 ARP4|FIAF|HARP|HFARP|NL2|PGAR|TGQTL|UNQ171|pp1158 19 Cytokine_Receptors

ANGPTL6 83854 angiopoietin like 6 AGF|ARP5 19 Cytokine_Receptors

APLNR 187 apelin receptor AGTRL1|APJ|APJR|HG11 11 Cytokine_Receptors

AR 367 androgen receptor AIS|AR8|DHTR|HUMARA|HYSP1|KD|NR3C4|SBMA|SMAX1|TFM X Cytokine_Receptors

AVPR1A 552 arginine vasopressin receptor 1A AVPR V1a|AVPR1|V1aR 12 Cytokine_Receptors

AVPR1B 553 arginine vasopressin receptor 1B AVPR3|V1bR 1 Cytokine_Receptors

AVPR2 554 arginine vasopressin receptor 2 ADHR|DI1|DIR|DIR3|NDI|V2R X Cytokine_Receptors

BMPR1A 657 bone morphogenetic protein receptor type 1A 10q23del|ACVRLK3|ALK3|CD292|SKR5 10 Cytokine_Receptors

BMPR1B 658 bone morphogenetic protein receptor type 1B ALK-6|ALK6|AMDD|BDA1D|BDA2|CDw293 4 Cytokine_Receptors

BMPR2 659 bone morphogenetic protein receptor type 2 BMPR-II|BMPR3|BMR2|BRK-3|POVD1|PPH1|T-ALK 2 Cytokine_Receptors

BRD8 10902 bromodomain containing 8 SMAP|SMAP2|p120 5 Cytokine_Receptors

C3AR1 719 complement C3a receptor 1 AZ3B|C3AR|HNFAG09 12 Cytokine_Receptors

C5AR1 728 complement C5a receptor 1 C5A|C5AR|C5R1|CD88 19 Cytokine_Receptors

CALCR 799 calcitonin receptor CRT|CT-R|CTR|CTR1 7 Cytokine_Receptors

CALCRL 10203 calcitonin receptor like receptor CGRPR|CRLR|LMPHM8 2 Cytokine_Receptors

ACKR2 1238 atypical chemokine receptor 2 CCBP2|CCR10|CCR9|CMKBR9|D6|hD6 3 Cytokine_Receptors

CCR1 1230 C-C motif chemokine receptor 1 CD191|CKR-1|CKR1|CMKBR1|HM145|MIP1aR|SCYAR1 3 Cytokine_Receptors

CCR10 2826 C-C motif chemokine receptor 10 GPR2 17 Cytokine_Receptors

CCR3 1232 C-C motif chemokine receptor 3 C C CKR3|CC-CKR-3|CD193|CKR 3|CKR3|CMKBR3 3 Cytokine_Receptors

CCR4 1233 C-C motif chemokine receptor 4 CC-CKR-4|CD194|CKR4|CMKBR4|ChemR13|HGCN:14099|K5-5 3 Cytokine_Receptors

CCR5 1234 C-C motif chemokine receptor 5 CC-CKR-5|CCCKR5|CCR-5|CD195|CKR-5|CKR5|CMKBR5|IDDM22 3 Cytokine_Receptors

CCR6 1235 C-C motif chemokine receptor 6 BN-1|C-C CKR-6|CC-CKR-6|CCR-6|CD196|CKR-L3|CKRL3|CMKBR6|DCR2|DRY6|GPR29|GPRCY4|STRL22 6 Cytokine_Receptors

CCR7 1236 C-C motif chemokine receptor 7 BLR2|CC-CKR-7|CCR-7|CD197|CDw197|CMKBR7|EBI1 17 Cytokine_Receptors

CCR8 1237 C-C motif chemokine receptor 8 CC-CKR-8|CCR-8|CDw198|CKRL1|CMKBR8|CMKBRL2|CY6|GPRCY6|TER1 3 Cytokine_Receptors

CCR9 10803 C-C motif chemokine receptor 9 CC-CKR-9|CDw199|GPR-9-6|GPR28 3 Cytokine_Receptors

ACKR4 51554 atypical chemokine receptor 4 CC-CKR-11|CCBP2|CCR-11|CCR10|CCR11|CCRL1|CCX CKR|CCX-CKR|CKR-11|PPR1|VSHK1 3 Cytokine_Receptors

CCRL2 9034 C-C motif chemokine receptor like 2 ACKR5|CKRX|CRAM|CRAM-A|CRAM-B|HCR 3 Cytokine_Receptors

CD40 958 CD40 molecule Bp50|CDW40|TNFRSF5|p50 20 Cytokine_Receptors

CMKLR1 1240 chemerin chemokine-like receptor 1 CHEMERINR|ChemR23|DEZ|RVER1 12 Cytokine_Receptors

CNTFR 1271 ciliary neurotrophic factor receptor - 9 Cytokine_Receptors

CRHR1 1394 corticotropin releasing hormone receptor 1 CRF-R|CRF-R-1|CRF-R1|CRF1|CRFR-1|CRFR1|CRH-R-1|CRH-R1|CRHR|CRHR1L 17 Cytokine_Receptors

CRHR2 1395 corticotropin releasing hormone receptor 2 CRF-RB|CRF2|CRFR2|HM-CRF 7 Cytokine_Receptors

CRIM1 51232 cysteine rich transmembrane BMP regulator 1 CRIM-1|S52 2 Cytokine_Receptors

CRLF1 9244 cytokine receptor like factor 1 CISS|CISS1|CLF|CLF-1|NR6|zcytor5 19 Cytokine_Receptors

CRLF2 64109 cytokine receptor like factor 2 CRL2|CRLF2Y|TSLPR X|Y Cytokine_Receptors

CRLF3 51379 cytokine receptor like factor 3 CREME-9|CREME9|CRLM9|CYTOR4|FRWS|p48.2 17 Cytokine_Receptors

CSF1R 1436 colony stimulating factor 1 receptor BANDDOS|C-FMS|CD115|CSF-1R|CSFR|FIM2|FMS|HDLS|M-CSF-R 5 Cytokine_Receptors

CSF2RA 1438 colony stimulating factor 2 receptor subunit alpha CD116|CDw116|CSF2R|CSF2RAX|CSF2RAY|CSF2RX|CSF2RY|GM-CSF-R-alpha|GMCSFR|GMCSFR-alpha|GMR|GMR-alpha|SMDP4|alphaGMR X|Y Cytokine_Receptors

CSF2RB 1439 colony stimulating factor 2 receptor subunit beta CD131|CDw131|IL3RB|IL5RB|SMDP5|betaGMR 22 Cytokine_Receptors

CSF3R 1441 colony stimulating factor 3 receptor CD114|GCSFR|SCN7 1 Cytokine_Receptors

CX3CR1 1524 C-X3-C motif chemokine receptor 1 CCRL1|CMKBRL1|CMKDR1|GPR13|GPRV28|V28 3 Cytokine_Receptors

CXCR3 2833 C-X-C motif chemokine receptor 3 CD182|CD183|CKR-L2|CMKAR3|GPR9|IP10-R|Mig-R|MigR X Cytokine_Receptors

CXCR4 7852 C-X-C motif chemokine receptor 4 CD184|D2S201E|FB22|HM89|HSY3RR|LAP-3|LAP3|LCR1|LESTR|NPY3R|NPYR|NPYRL|NPYY3R|WHIM|WHIMS 2 Cytokine_Receptors

CXCR5 643 C-X-C motif chemokine receptor 5 BLR1|CD185|MDR15 11 Cytokine_Receptors

CXCR6 10663 C-X-C motif chemokine receptor 6 BONZO|CD186|STRL33|TYMSTR 3 Cytokine_Receptors

ACKR3 57007 atypical chemokine receptor 3 CMKOR1|CXC-R7|CXCR-7|CXCR7|GPR159|RDC-1|RDC1 2 Cytokine_Receptors

CYSLTR1 10800 cysteinyl leukotriene receptor 1 CYSLT1|CYSLT1R|CYSLTR|HMTMF81 X Cytokine_Receptors

CYSLTR2 57105 cysteinyl leukotriene receptor 2 CYSLT2|CYSLT2R|GPCR21|HG57|HPN321|KPG_011|PSEC0146|hGPCR21 13 Cytokine_Receptors

ACKR1 2532 atypical chemokine receptor 1 (Duffy blood group) CCBP1|CD234|DARC|DARC/ACKR1|Dfy|FY|GPD|GpFy|WBCQ1 1 Cytokine_Receptors

EDNRA 1909 endothelin receptor type A ET-A|ETA|ETA-R|ETAR|ETRA|MFDA|hET-AR 4 Cytokine_Receptors

EDNRB 1910 endothelin receptor type B ABCDS|ET-B|ET-BR|ETB|ETB1|ETBR|ETRB|HSCR|HSCR2|WS4A 13 Cytokine_Receptors

EGFR 1956 epidermal growth factor receptor ERBB|ERBB1|HER1|NISBD2|PIG61|mENA 7 Cytokine_Receptors

ENG 2022 endoglin END|HHT1|ORW1 9 Cytokine_Receptors

EPOR 2057 erythropoietin receptor EPO-R 19 Cytokine_Receptors

ESR1 2099 estrogen receptor 1 ER|ESR|ESRA|ESTRR|Era|NR3A1 6 Cytokine_Receptors

ESR2 2100 estrogen receptor 2 ER-BETA|ESR-BETA|ESRB|ESTRB|Erb|NR3A2|ODG8 14 Cytokine_Receptors

ESRRA 2101 estrogen related receptor alpha ERR1|ERRa|ERRalpha|ESRL1|NR3B1 11 Cytokine_Receptors

ESRRB 2103 estrogen related receptor beta DFNB35|ERR beta-2|ERR2|ERRb|ERRbeta2|ESRL2|NR3B2 14 Cytokine_Receptors

ESRRG 2104 estrogen related receptor gamma ERR-gamma|ERR3|ERRg|ERRgamma|NR3B3 1 Cytokine_Receptors

FGFR1 2260 fibroblast growth factor receptor 1 BFGFR|CD331|CEK|ECCL|FGFBR|FGFR-1|FLG|FLT-2|FLT2|HBGFR|HH2|HRTFDS|KAL2|N-SAM|OGD|bFGF-R-1 8 Cytokine_Receptors

FGFR2 2263 fibroblast growth factor receptor 2 BBDS|BEK|BFR-1|CD332|CEK3|CFD1|ECT1|JWS|K-SAM|KGFR|TK14|TK25 10 Cytokine_Receptors

FGFR3 2261 fibroblast growth factor receptor 3 ACH|CD333|CEK2|HSFGFR3EX|JTK4 4 Cytokine_Receptors

FGFR4 2264 fibroblast growth factor receptor 4 CD334|JTK2|TKF 5 Cytokine_Receptors

FGFRL1 53834 fibroblast growth factor receptor like 1 FGFR-5|FGFR5|FHFR 4 Cytokine_Receptors

FLT1 2321 fms related receptor tyrosine kinase 1 FLT|FLT-1|VEGFR-1|VEGFR1 13 Cytokine_Receptors

FLT3 2322 fms related receptor tyrosine kinase 3 CD135|FLK-2|FLK2|STK1 13 Cytokine_Receptors

FLT4 2324 fms related receptor tyrosine kinase 4 CHTD7|FLT-4|FLT41|LMPH1A|LMPHM1|PCL|VEGFR-3|VEGFR3 5 Cytokine_Receptors

FPR1 2357 formyl peptide receptor 1 FMLP|FPR 19 Cytokine_Receptors

FPR2 2358 formyl peptide receptor 2 ALXR|FMLP-R-II|FMLPX|FPR2A|FPRH1|FPRH2|FPRL1|HM63|LXA4R 19 Cytokine_Receptors

FPR2 2358 formyl peptide receptor 2 ALXR|FMLP-R-II|FMLPX|FPR2A|FPRH1|FPRH2|FPRL1|HM63|LXA4R 19 Cytokine_Receptors

FSHR 2492 follicle stimulating hormone receptor FSHR1|FSHRO|LGR1|ODG1 2 Cytokine_Receptors

GALR2 8811 galanin receptor 2 GAL2-R|GALNR2|GALR-2 17 Cytokine_Receptors

GALR3 8484 galanin receptor 3 - 22 Cytokine_Receptors

GCGR 2642 glucagon receptor GGR|GL-R 17 Cytokine_Receptors

GHR 2690 growth hormone receptor GHBP|GHIP 5 Cytokine_Receptors

GHRHR 2692 growth hormone releasing hormone receptor GHRFR|GRFR|IGHD1B|IGHD4 7 Cytokine_Receptors

GHSR 2693 growth hormone secretagogue receptor GHDP 3 Cytokine_Receptors

GIPR 2696 gastric inhibitory polypeptide receptor PGQTL2 19 Cytokine_Receptors

GLP1R 2740 glucagon like peptide 1 receptor GLP-1|GLP-1-R|GLP-1R 6 Cytokine_Receptors

GLP2R 9340 glucagon like peptide 2 receptor - 17 Cytokine_Receptors

GNRHR 2798 gonadotropin releasing hormone receptor GNRHR1|GRHR|HH7|LHRHR|LRHR 4 Cytokine_Receptors

GPER1 2852 G protein-coupled estrogen receptor 1 CEPR|CMKRL2|DRY12|FEG-1|GPCR-Br|GPER|GPR30|LERGU|LERGU2|LyGPR|mER 7 Cytokine_Receptors

GPR17 2840 G protein-coupled receptor 17 - 2 Cytokine_Receptors

GPR32 2854 G protein-coupled receptor 32 RVDR1 19 Cytokine_Receptors

GPR33 2856 G protein-coupled receptor 33 - 14 Cytokine_Receptors

PTGDR2 11251 prostaglandin D2 receptor 2 CD294|CRTH2|DL1R|DP2|GPR44 11 Cytokine_Receptors

C5AR2 27202 complement component 5a receptor 2 C5L2|GPF77|GPR77 19 Cytokine_Receptors

HNF4A 3172 hepatocyte nuclear factor 4 alpha FRTS4|HNF4|HNF4a7|HNF4a8|HNF4a9|HNF4alpha|MODY|MODY1|NR2A1|NR2A21|TCF|TCF14 20 Cytokine_Receptors

HNF4G 3174 hepatocyte nuclear factor 4 gamma NR2A2|NR2A3 8 Cytokine_Receptors

HTR3A 3359 5-hydroxytryptamine receptor 3A 5-HT-3|5-HT3A|5-HT3R|5HT3R|HTR3 11 Cytokine_Receptors

HTR3B 9177 5-hydroxytryptamine receptor 3B 5-HT3B 11 Cytokine_Receptors

HTR3C 170572 5-hydroxytryptamine receptor 3C - 3 Cytokine_Receptors

HTR3D 200909 5-hydroxytryptamine receptor 3D 5HT3D 3 Cytokine_Receptors

HTR3E 285242 5-hydroxytryptamine receptor 3E 5-HT3-E|5-HT3E|5-HT3c1 3 Cytokine_Receptors

IFNAR1 3454 interferon alpha and beta receptor subunit 1 AVP|IFN-alpha-REC|IFNAR|IFNBR|IFRC 21 Cytokine_Receptors

IFNAR2 3455 interferon alpha and beta receptor subunit 2 IFN-R|IFN-alpha-REC|IFNABR|IFNARB|IMD45 21 Cytokine_Receptors

IFNGR1 3459 interferon gamma receptor 1 CD119|IFNGR|IMD27A|IMD27B 6 Cytokine_Receptors

IFNGR2 3460 interferon gamma receptor 2 AF-1|IFGR2|IFNGT1|IMD28 21 Cytokine_Receptors

IGF1R 3480 insulin like growth factor 1 receptor CD221|IGFIR|IGFR|JTK13 15 Cytokine_Receptors

IGF2R 3482 insulin like growth factor 2 receptor CD222|CI-M6PR|CIMPR|M6P-R|M6P/IGF2R|MPR 300|MPR1|MPR300|MPRI 6 Cytokine_Receptors

IL10RA 3587 interleukin 10 receptor subunit alpha CD210|CD210a|CDW210A|HIL-10R|IL-10R1|IL10R 11 Cytokine_Receptors

IL10RB 3588 interleukin 10 receptor subunit beta CDW210B|CRF2-4|CRFB4|D21S58|D21S66|IL-10R2 21 Cytokine_Receptors

IL11RA 3590 interleukin 11 receptor subunit alpha CRSDA 9 Cytokine_Receptors

IL12RB1 3594 interleukin 12 receptor subunit beta 1 CD212|IL-12R-BETA1|IL12RB|IMD30 19 Cytokine_Receptors

IL12RB2 3595 interleukin 12 receptor subunit beta 2 - 1 Cytokine_Receptors

IL13RA1 3597 interleukin 13 receptor subunit alpha 1 CD213A1|CT19|IL-13Ra|NR4 X Cytokine_Receptors

IL13RA2 3598 interleukin 13 receptor subunit alpha 2 CD213A2|CT19|IL-13R|IL13BP X Cytokine_Receptors

IL15RA 3601 interleukin 15 receptor subunit alpha CD215 10 Cytokine_Receptors

IL2RB 3560 interleukin 2 receptor subunit beta CD122|IL15RB|IMD63|P70-75 22 Cytokine_Receptors

IL17RA 23765 interleukin 17 receptor A CANDF5|CD217|CDw217|IL-17RA|IL17R|IMD51|hIL-17R 22 Cytokine_Receptors

IL17RB 55540 interleukin 17 receptor B CRL4|EVI27|IL17BR|IL17RH1 3 Cytokine_Receptors

IL17RC 84818 interleukin 17 receptor C CANDF9|IL17-RL|IL17RL 3 Cytokine_Receptors

IL17RD 54756 interleukin 17 receptor D HH18|IL-17RD|IL17RLM|SEF 3 Cytokine_Receptors

IL17RE 132014 interleukin 17 receptor E - 3 Cytokine_Receptors

IL18R1 8809 interleukin 18 receptor 1 CD218a|CDw218a|IL-18R-alpha|IL-18Ralpha|IL-1Rrp|IL18RA|IL18Ralpha2|IL1RRP 2 Cytokine_Receptors

IL18RAP 8807 interleukin 18 receptor accessory protein ACPL|CD218b|CDw218b|IL-18R-beta|IL-18RAcP|IL-18Rbeta|IL-1R-7|IL-1R7|IL-1RAcPL|IL18RB 2 Cytokine_Receptors

IL1R1 3554 interleukin 1 receptor type 1 CD121A|D2S1473|IL-1R-alpha|IL1R|IL1RA|P80 2 Cytokine_Receptors

IL1R2 7850 interleukin 1 receptor type 2 CD121b|CDw121b|IL-1R-2|IL-1RT-2|IL-1RT2|IL1R2c|IL1RB 2 Cytokine_Receptors

IL1RAP 3556 interleukin 1 receptor accessory protein C3orf13|IL-1RAcP|IL1R3 3 Cytokine_Receptors

IL1RL1 9173 interleukin 1 receptor like 1 DER4|FIT-1|IL33R|ST2|ST2L|ST2V|T1 2 Cytokine_Receptors

IL1RL2 8808 interleukin 1 receptor like 2 IL-1Rrp2|IL-36R|IL1R-rp2|IL1RRP2 2 Cytokine_Receptors

IL20RA 53832 interleukin 20 receptor subunit alpha CRF2-8|IL-20R-alpha|IL-20R1|IL-20RA 6 Cytokine_Receptors

IL20RB 53833 interleukin 20 receptor subunit beta DIRS1|FNDC6|IL-20R2 3 Cytokine_Receptors

IL21R 50615 interleukin 21 receptor CD360|IMD56|NILR 16 Cytokine_Receptors

IL22RA1 58985 interleukin 22 receptor subunit alpha 1 CRF2-9|IL22R|IL22R1 1 Cytokine_Receptors

IL22RA2 116379 interleukin 22 receptor subunit alpha 2 CRF2-10|CRF2-S1|CRF2X|IL-22BP|IL-22R-alpha-2|IL-22RA2|ZCYTOR16 6 Cytokine_Receptors

IL23R 149233 interleukin 23 receptor - 1 Cytokine_Receptors

IL27RA 9466 interleukin 27 receptor subunit alpha CRL1|IL-27RA|IL27R|TCCR|WSX1|zcytor1 19 Cytokine_Receptors

IFNLR1 163702 interferon lambda receptor 1 CRF2/12|IFNLR|IL-28R1|IL28RA|LICR2 1 Cytokine_Receptors

IL2RA 3559 interleukin 2 receptor subunit alpha CD25|IDDM10|IL2R|IMD41|TCGFR|p55 10 Cytokine_Receptors

IL2RB 3560 interleukin 2 receptor subunit beta CD122|IL15RB|IMD63|P70-75 22 Cytokine_Receptors

IL2RG 3561 interleukin 2 receptor subunit gamma CD132|CIDX|IL-2RG|IMD4|P64|SCIDX|SCIDX1 X Cytokine_Receptors

IL31RA 133396 interleukin 31 receptor A CRL|CRL3|GLM-R|GLMR|GPL|IL-31RA|PLCA2|PRO21384|hGLM-R 5 Cytokine_Receptors

IL3RA 3563 interleukin 3 receptor subunit alpha CD123|IL3R|IL3RAY|IL3RX|IL3RY|hIL-3Ra X|Y Cytokine_Receptors

IL4R 3566 interleukin 4 receptor CD124|IL-4RA|IL4RA 16 Cytokine_Receptors

IL5RA 3568 interleukin 5 receptor subunit alpha CD125|CDw125|HSIL5R3|IL5R 3 Cytokine_Receptors

IL6R 3570 interleukin 6 receptor CD126|IL-6R-1|IL-6RA|IL6Q|IL6RA|IL6RQ|gp80 1 Cytokine_Receptors

IL7R 3575 interleukin 7 receptor CD127|CDW127|IL-7R-alpha|IL7RA|ILRA 5 Cytokine_Receptors

CXCR1 3577 C-X-C motif chemokine receptor 1 C-C|C-C-CKR-1|CD128|CD181|CDw128a|CKR-1|CMKAR1|IL8R1|IL8RA|IL8RBA 2 Cytokine_Receptors

CXCR2 3579 C-X-C motif chemokine receptor 2 CD182|CDw128b|CMKAR2|IL8R2|IL8RA|IL8RB 2 Cytokine_Receptors

IL9R 3581 interleukin 9 receptor CD129|IL-9R X|Y Cytokine_Receptors

INSR 3643 insulin receptor CD220|HHF5 19 Cytokine_Receptors

KDR 3791 kinase insert domain receptor CD309|FLK1|VEGFR|VEGFR2 4 Cytokine_Receptors

LEPR 3953 leptin receptor CD295|LEP-R|LEPRD|OB-R|OBR 1 Cytokine_Receptors

LGR4 55366 leucine rich repeat containing G protein-coupled receptor 4 BNMD17|GPR48 11 Cytokine_Receptors

LGR5 8549 leucine rich repeat containing G protein-coupled receptor 5 FEX|GPR49|GPR67|GRP49|HG38 12 Cytokine_Receptors

LGR6 59352 leucine rich repeat containing G protein-coupled receptor 6 GPCR|VTS20631 1 Cytokine_Receptors

LHCGR 3973 luteinizing hormone/choriogonadotropin receptor HHG|LCGR|LGR2|LH/CG-R|LH/CGR|LHR|LHRHR|LSH-R|ULG5 2 Cytokine_Receptors

LIFR 3977 LIF receptor subunit alpha CD118|LIF-R|SJS2|STWS|SWS 5 Cytokine_Receptors

LTB4R 1241 leukotriene B4 receptor BLT1|BLTR|CMKRL1|GPR16|LTB4R1|LTBR1|P2RY7|P2Y7 14 Cytokine_Receptors

LTB4R2 56413 leukotriene B4 receptor 2 BLT2|BLTR2|JULF2|KPG_004|LTB4-R 2|LTB4-R2|NOP9 14 Cytokine_Receptors

LTBR 4055 lymphotoxin beta receptor D12S370|LT-BETA-R|TNF-R-III|TNFCR|TNFR-RP|TNFR2-RP|TNFR3|TNFRSF3 12 Cytokine_Receptors

MC1R 4157 melanocortin 1 receptor CMM5|MSH-R|SHEP2 16 Cytokine_Receptors

MC2R 4158 melanocortin 2 receptor ACTHR 18 Cytokine_Receptors

MC3R 4159 melanocortin 3 receptor BMIQ9|MC3|MC3-R|OB20|OQTL 20 Cytokine_Receptors

MC4R 4160 melanocortin 4 receptor BMIQ20 18 Cytokine_Receptors

MCHR1 2847 melanin concentrating hormone receptor 1 GPR24|MCH-1R|MCH1R|SLC-1|SLC1 22 Cytokine_Receptors

MCHR2 84539 melanin concentrating hormone receptor 2 GPR145|GPRv17|MCH-2R|MCH-R2|MCH2|MCH2R|MCHR-2|SLT 6 Cytokine_Receptors

MET 4233 MET proto-oncogene, receptor tyrosine kinase AUTS9|DFNB97|HGFR|RCCP2|c-Met 7 Cytokine_Receptors

MLNR 2862 motilin receptor GPR38|MTLR1 13 Cytokine_Receptors

MPL 4352 MPL proto-oncogene, thrombopoietin receptor C-MPL|CD110|MPLV|THCYT2|THPOR|TPOR 1 Cytokine_Receptors

MTNR1A 4543 melatonin receptor 1A MEL-1A-R|MT1 4 Cytokine_Receptors

MTNR1B 4544 melatonin receptor 1B FGQTL2|MEL-1B-R|MT2 11 Cytokine_Receptors

NGFR 4804 nerve growth factor receptor CD271|Gp80-LNGFR|TNFRSF16|p75(NTR)|p75NTR 17 Cytokine_Receptors

NMBR 4829 neuromedin B receptor BB1|BB1R|NMB-R 6 Cytokine_Receptors

NPR1 4881 natriuretic peptide receptor 1 ANPRA|ANPa|GUC2A|GUCY2A|NPRA 1 Cytokine_Receptors

NPR3 4883 natriuretic peptide receptor 3 ANP-C|ANPR-C|ANPRC|C5orf23|GUCY2B|NPR-C|NPRC 5 Cytokine_Receptors

NR0B1 190 nuclear receptor subfamily 0 group B member 1 AHC|AHCH|AHX|DAX-1|DAX1|DSS|GTD|HHG|NROB1|SRXY2 X Cytokine_Receptors

NR0B2 8431 nuclear receptor subfamily 0 group B member 2 SHP|SHP1 1 Cytokine_Receptors

NR1D1 9572 nuclear receptor subfamily 1 group D member 1 EAR1|REVERBA|REVERBalpha|THRA1|THRAL|ear-1|hRev 17 Cytokine_Receptors

NR1D2 9975 nuclear receptor subfamily 1 group D member 2 BD73|EAR-1R|REVERBB|REVERBbeta|RVR 3 Cytokine_Receptors

NR1H2 7376 nuclear receptor subfamily 1 group H member 2 LXR-b|LXRB|NER|NER-I|RIP15|UNR 19 Cytokine_Receptors

NR1H3 10062 nuclear receptor subfamily 1 group H member 3 LXR-a|LXRA|RLD-1 11 Cytokine_Receptors

NR1H4 9971 nuclear receptor subfamily 1 group H member 4 BAR|FXR|HRR-1|HRR1|PFIC5|RIP14 12 Cytokine_Receptors

NR1I2 8856 nuclear receptor subfamily 1 group I member 2 BXR|ONR1|PAR|PAR1|PAR2|PARq|PRR|PXR|SAR|SXR 3 Cytokine_Receptors

NR1I3 9970 nuclear receptor subfamily 1 group I member 3 CAR|CAR1|MB67 1 Cytokine_Receptors

NR2C1 7181 nuclear receptor subfamily 2 group C member 1 TR2 12 Cytokine_Receptors

NR2C2 7182 nuclear receptor subfamily 2 group C member 2 TAK1|TR4 3 Cytokine_Receptors

NR2E1 7101 nuclear receptor subfamily 2 group E member 1 TLL|TLX|XTLL 6 Cytokine_Receptors

NR2E3 10002 nuclear receptor subfamily 2 group E member 3 ESCS|PNR|RNR|RP37|rd7 15 Cytokine_Receptors

NR2F1 7025 nuclear receptor subfamily 2 group F member 1 BBOAS|BBSOAS|COUP-TFI|COUPTF1|EAR-3|EAR3|ERBAL3|SVP44|TCFCOUP1|TFCOUP1 5 Cytokine_Receptors

NR2F2 7026 nuclear receptor subfamily 2 group F member 2 ARP-1|ARP1|CHTD4|COUPTF2|COUPTFB|COUPTFII|NF-E3|SVP40|TFCOUP2 15 Cytokine_Receptors

NR2F6 2063 nuclear receptor subfamily 2 group F member 6 EAR-2|EAR2|ERBAL2 19 Cytokine_Receptors

NR3C1 2908 nuclear receptor subfamily 3 group C member 1 GCCR|GCR|GCRST|GR|GRL 5 Cytokine_Receptors

NR3C2 4306 nuclear receptor subfamily 3 group C member 2 MCR|MLR|MR|NR3C2VIT 4 Cytokine_Receptors

NR4A1 3164 nuclear receptor subfamily 4 group A member 1 GFRP1|HMR|N10|NAK-1|NGFIB|NP10|NUR77|TR3 12 Cytokine_Receptors

NR4A2 4929 nuclear receptor subfamily 4 group A member 2 HZF-3|NOT|NURR1|RNR1|TINUR 2 Cytokine_Receptors

NR4A3 8013 nuclear receptor subfamily 4 group A member 3 CHN|CSMF|MINOR|NOR1|TEC 9 Cytokine_Receptors

NR5A1 2516 nuclear receptor subfamily 5 group A member 1 AD4BP|ELP|FTZ1|FTZF1|POF7|SF-1|SF1|SPGF8|SRXX4|SRXY3|hSF-1 9 Cytokine_Receptors

NR5A2 2494 nuclear receptor subfamily 5 group A member 2 B1F|B1F2|CPF|FTF|FTZ-F1|FTZ-F1beta|LRH-1|LRH1|hB1F-2 1 Cytokine_Receptors

NR6A1 2649 nuclear receptor subfamily 6 group A member 1 CT150|GCNF|GCNF1|NR61|RTR|hGCNF|hRTR 9 Cytokine_Receptors

NRP1 8829 neuropilin 1 BDCA4|CD304|NP1|NRP|VEGF165R 10 Cytokine_Receptors

NRP2 8828 neuropilin 2 NP2|NPN2|PRO2714|VEGF165R2 2 Cytokine_Receptors

OGFR 11054 opioid growth factor receptor - 20 Cytokine_Receptors

OPRD1 4985 opioid receptor delta 1 DOP|DOR|DOR1|OPRD 1 Cytokine_Receptors

OPRK1 4986 opioid receptor kappa 1 K-OR-1|KOP|KOR|KOR-1|KOR1|OPRK 8 Cytokine_Receptors

OPRL1 4987 opioid related nociceptin receptor 1 KOR-3|KOR3|NOCIR|NOP|NOPr|OOR|OPRL|ORL1 20 Cytokine_Receptors

OPRM1 4988 opioid receptor mu 1 LMOR|M-OR-1|MOP|MOR|MOR1|OPRM 6 Cytokine_Receptors

OSMR 9180 oncostatin M receptor IL-31R-beta|IL-31RB|OSMRB|OSMRbeta|PLCA1 5 Cytokine_Receptors

OXTR 5021 oxytocin receptor OT-R 3 Cytokine_Receptors

PGR 5241 progesterone receptor NR3C3|PR 11 Cytokine_Receptors

PGRMC2 10424 progesterone receptor membrane component 2 DG6|PMBP 4 Cytokine_Receptors

PLAUR 5329 plasminogen activator, urokinase receptor CD87|U-PAR|UPAR|URKR 19 Cytokine_Receptors

PLXNA1 5361 plexin A1 NOV|NOVP|PLEXIN-A1|PLXN1 3 Cytokine_Receptors

PLXNA2 5362 plexin A2 OCT|PLXN2 1 Cytokine_Receptors

PLXNA3 55558 plexin A3 6.3|HSSEXGENE|PLXN3|PLXN4|XAP-6 X Cytokine_Receptors

PLXNA4 91584 plexin A4 FAYV2820|PLEXA4|PLXNA4A|PLXNA4B|PRO34003 7 Cytokine_Receptors

PLXNB1 5364 plexin B1 PLEXIN-B1|PLXN5|SEP 3 Cytokine_Receptors

PLXNB2 23654 plexin B2 MM1|Nbla00445|PLEXB2|dJ402G11.3 22 Cytokine_Receptors

PLXNB3 5365 plexin B3 PLEXB3|PLEXR|PLXN6 X Cytokine_Receptors

PLXNC1 10154 plexin C1 CD232|PLXN-C1|VESPR 12 Cytokine_Receptors

PLXND1 23129 plexin D1 PLEXD1 3 Cytokine_Receptors

PPARA 5465 peroxisome proliferator activated receptor alpha NR1C1|PPAR|PPARalpha|hPPAR 22 Cytokine_Receptors

PPARD 5467 peroxisome proliferator activated receptor delta FAAR|NR1C2|NUC1|NUCI|NUCII|PPARB 6 Cytokine_Receptors

PPARG 5468 peroxisome proliferator activated receptor gamma CIMT1|GLM1|NR1C3|PPARG1|PPARG2|PPARG5|PPARgamma 3 Cytokine_Receptors

PRLHR 2834 prolactin releasing hormone receptor GPR10|GR3|PrRPR 10 Cytokine_Receptors

PRLR 5618 prolactin receptor HPRL|MFAB|RI-PRLR|hPRLrI 5 Cytokine_Receptors

PTAFR 5724 platelet activating factor receptor PAFR 1 Cytokine_Receptors

PTGDR 5729 prostaglandin D2 receptor AS1|ASRT1|DP|DP1|PTGDR1 14 Cytokine_Receptors

PTGDS 5730 prostaglandin D2 synthase L-PGDS|LPGDS|PDS|PGD2|PGDS|PGDS2 9 Cytokine_Receptors

PTGER1 5731 prostaglandin E receptor 1 EP1 19 Cytokine_Receptors

PTGER2 5732 prostaglandin E receptor 2 EP2 14 Cytokine_Receptors

PTGER3 5733 prostaglandin E receptor 3 EP3|EP3-I|EP3-II|EP3-III|EP3-IV|EP3-VI|EP3e|PGE2-R|lnc003875 1 Cytokine_Receptors

PTGER4 5734 prostaglandin E receptor 4 EP4|EP4R 5 Cytokine_Receptors

PTGFR 5737 prostaglandin F receptor FP 1 Cytokine_Receptors

PTH1R 5745 parathyroid hormone 1 receptor EKNS|PFE|PTHR|PTHR1 3 Cytokine_Receptors

PTH2R 5746 parathyroid hormone 2 receptor PTHR2 2 Cytokine_Receptors

RARA 5914 retinoic acid receptor alpha NR1B1|RAR 17 Cytokine_Receptors

RARB 5915 retinoic acid receptor beta HAP|MCOPS12|NR1B2|RARbeta1|RRB2 3 Cytokine_Receptors

RARG 5916 retinoic acid receptor gamma NR1B3|RARC 12 Cytokine_Receptors

ROBO1 6091 roundabout guidance receptor 1 DUTT1|SAX3 3 Cytokine_Receptors

ROBO2 6092 roundabout guidance receptor 2 SAX3 3 Cytokine_Receptors

ROBO3 64221 roundabout guidance receptor 3 HGPPS|HGPPS1|HGPS|RBIG1|RIG1 11 Cytokine_Receptors

RORA 6095 RAR related orphan receptor A IDDECA|NR1F1|ROR1|ROR2|ROR3|RZR-ALPHA|RZRA 15 Cytokine_Receptors

RORB 6096 RAR related orphan receptor B EIG15|NR1F2|ROR-BETA|RZR-BETA|RZRB|bA133M9.1 9 Cytokine_Receptors

RORC 6097 RAR related orphan receptor C IMD42|NR1F3|RORG|RZR-GAMMA|RZRG|TOR 1 Cytokine_Receptors

RXFP1 59350 relaxin family peptide receptor 1 LGR7|RXFPR1 4 Cytokine_Receptors

RXFP2 122042 relaxin family peptide receptor 2 GPR106|GREAT|INSL3R|LGR8|LGR8.1|RXFPR2 13 Cytokine_Receptors

RXFP3 51289 relaxin family peptide receptor 3 GPCR135|RLN3R1|RXFPR3|SALPR 5 Cytokine_Receptors

RXRA 6256 retinoid X receptor alpha NR2B1 9 Cytokine_Receptors

RXRB 6257 retinoid X receptor beta DAUDI6|H-2RIIBP|NR2B2|RCoR-1 6 Cytokine_Receptors

RXRG 6258 retinoid X receptor gamma NR2B3|RXRC 1 Cytokine_Receptors

S1PR1 1901 sphingosine-1-phosphate receptor 1 CD363|CHEDG1|D1S3362|ECGF1|EDG-1|EDG1|S1P1 1 Cytokine_Receptors

S1PR2 9294 sphingosine-1-phosphate receptor 2 AGR16|DFNB68|EDG-5|EDG5|Gpcr13|H218|LPB2|S1P2 19 Cytokine_Receptors

SCTR 6344 secretin receptor SR 2 Cytokine_Receptors

SDC1 6382 syndecan 1 CD138|SDC|SYND1|syndecan 2 Cytokine_Receptors

SDC2 6383 syndecan 2 CD362|HSPG|HSPG1|SYND2 8 Cytokine_Receptors

SDC3 9672 syndecan 3 SDCN|SYND3 1 Cytokine_Receptors

SDC4 6385 syndecan 4 SYND4 20 Cytokine_Receptors

SORT1 6272 sortilin 1 Gp95|LDLCQ6|NT3|NTR3 1 Cytokine_Receptors

SSTR1 6751 somatostatin receptor 1 SRIF-2|SS-1-R|SS1-R|SS1R 14 Cytokine_Receptors

SSTR2 6752 somatostatin receptor 2 - 17 Cytokine_Receptors

SSTR5 6755 somatostatin receptor 5 SS-5-R 16 Cytokine_Receptors

ST2 6761 - - 11 Cytokine_Receptors

TACR1 6869 tachykinin receptor 1 NK1R|NKIR|SPR|TAC1R 2 Cytokine_Receptors

TEK 7010 TEK receptor tyrosine kinase CD202B|GLC3E|TIE-2|TIE2|VMCM|VMCM1 9 Cytokine_Receptors

TGFBR1 7046 transforming growth factor beta receptor 1 AAT5|ACVRLK4|ALK-5|ALK5|ESS1|LDS1|LDS1A|LDS2A|MSSE|SKR4|TBR-i|TBRI|TGFR-1|tbetaR-I 9 Cytokine_Receptors

TGFBR2 7048 transforming growth factor beta receptor 2 AAT3|FAA3|LDS1B|LDS2|LDS2B|MFS2|RIIC|TAAD2|TBR-ii|TBRII|TGFR-2|TGFbeta-RII 3 Cytokine_Receptors

TGFBR3 7049 transforming growth factor beta receptor 3 BGCAN|betaglycan 1 Cytokine_Receptors

THRA 7067 thyroid hormone receptor alpha AR7|CHNG6|EAR7|ERB-T-1|ERBA|ERBA1|NR1A1|THRA1|THRA2|c-ERBA-1 17 Cytokine_Receptors

THRB 7068 thyroid hormone receptor beta C-ERBA-2|C-ERBA-BETA|ERBA2|GRTH|NR1A2|PRTH|THR1|THRB1|THRB2 3 Cytokine_Receptors

TIE1 7075 tyrosine kinase with immunoglobulin like and EGF like domains 1 JTK14|TIE 1 Cytokine_Receptors

TNFRSF10A 8797 TNF receptor superfamily member 10a APO2|CD261|DR4|TRAILR-1|TRAILR1 8 Cytokine_Receptors

TNFRSF10B 8795 TNF receptor superfamily member 10b CD262|DR5|KILLER|KILLER/DR5|TRAIL-R2|TRAILR2|TRICK2|TRICK2A|TRICK2B|TRICKB|ZTNFR9 8 Cytokine_Receptors

TNFRSF10C 8794 TNF receptor superfamily member 10c CD263|DCR1|DCR1-TNFR|LIT|TRAIL-R3|TRAILR3|TRID 8 Cytokine_Receptors

TNFRSF10D 8793 TNF receptor superfamily member 10d CD264|DCR2|TRAIL-R4|TRAILR4|TRUNDD 8 Cytokine_Receptors

TNFRSF11A 8792 TNF receptor superfamily member 11a CD265|FEO|LOH18CR1|ODFR|OFE|OPTB7|OSTS|PDB2|RANK|TRANCER 18 Cytokine_Receptors

TNFRSF12A 51330 TNF receptor superfamily member 12A CD266|FN14|TWEAKR 16 Cytokine_Receptors

TNFRSF13B 23495 TNF receptor superfamily member 13B CD267|CVID|CVID2|IGAD2|RYZN|TACI|TNFRSF14B 17 Cytokine_Receptors

TNFRSF13C 115650 TNF receptor superfamily member 13C BAFF-R|BAFFR|BROMIX|CD268|CVID4|prolixin 22 Cytokine_Receptors

TNFRSF14 8764 TNF receptor superfamily member 14 ATAR|CD270|HVEA|HVEM|LIGHTR|TR2 1 Cytokine_Receptors

TNFRSF17 608 TNF receptor superfamily member 17 BCM|BCMA|CD269|TNFRSF13A 16 Cytokine_Receptors

TNFRSF18 8784 TNF receptor superfamily member 18 AITR|CD357|ENERGEN|GITR|GITR-D 1 Cytokine_Receptors

TNFRSF19 55504 TNF receptor superfamily member 19 TAJ|TAJ-alpha|TRADE|TROY 13 Cytokine_Receptors

TNFRSF1A 7132 TNF receptor superfamily member 1A CD120a|FPF|TBP1|TNF-R|TNF-R-I|TNF-R55|TNFAR|TNFR1|TNFR55|TNFR60|p55|p55-R|p60 12 Cytokine_Receptors

TNFRSF1B 7133 TNF receptor superfamily member 1B CD120b|TBPII|TNF-R-II|TNF-R75|TNFBR|TNFR1B|TNFR2|TNFR80|p75|p75TNFR 1 Cytokine_Receptors

TNFRSF21 27242 TNF receptor superfamily member 21 BM-018|CD358|DR6 6 Cytokine_Receptors

TNFRSF25 8718 TNF receptor superfamily member 25 APO-3|DDR3|DR3|GEF720|LARD|PLEKHG5|TNFRSF12|TR3|TRAMP|WSL-1|WSL-LR 1 Cytokine_Receptors

TNFRSF4 7293 TNF receptor superfamily member 4 ACT35|CD134|IMD16|OX40|TXGP1L 1 Cytokine_Receptors

TNFRSF6B 8771 TNF receptor superfamily member 6b DCR3|DJ583P15.1.1|M68|M68E|TR6 20 Cytokine_Receptors

TNFRSF8 943 TNF receptor superfamily member 8 CD30|D1S166E|Ki-1 1 Cytokine_Receptors

TNFRSF9 3604 TNF receptor superfamily member 9 4-1BB|CD137|CDw137|ILA 1 Cytokine_Receptors

TRHR 7201 thyrotropin releasing hormone receptor CHNG7|TRH-R 8 Cytokine_Receptors

TSHR 7253 thyroid stimulating hormone receptor CHNG1|LGR3|hTSHR-I 14 Cytokine_Receptors

TUBB3 10381 tubulin beta 3 class III CDCBM|CDCBM1|CFEOM3|CFEOM3A|FEOM3|TUBB4|beta-4 16 Cytokine_Receptors

VDR 7421 vitamin D receptor NR1I1|PPP1R163 12 Cytokine_Receptors

VIPR1 7433 vasoactive intestinal peptide receptor 1 HVR1|II|PACAP-R-2|PACAP-R2|RDC1|V1RG|VAPC1|VIP-R-1|VIPR|VIRG|VPAC1|VPAC1R|VPCAP1R 3 Cytokine_Receptors

VIPR2 7434 vasoactive intestinal peptide receptor 2 C16DUPq36.3|DUP7q36.3|PACAP-R-3|PACAP-R3|VIP-R-2|VPAC2|VPAC2R|VPCAP2R 7 Cytokine_Receptors

XCR1 2829 X-C motif chemokine receptor 1 CCXCR1|GPR5 3 Cytokine_Receptors

IFNA10 3446 interferon alpha 10 IFN-alphaC 9 Interferons

IFNA13 3447 interferon alpha 13 - 9 Interferons

IFNA14 3448 interferon alpha 14 IFN-alphaH|LEIF2H 9 Interferons

IFNA16 3449 interferon alpha 16 IFN-alpha-16|IFN-alphaO 9 Interferons

IFNA17 3451 interferon alpha 17 IFN-alphaI|IFNA|INFA|LEIF2C1 9 Interferons

IFNA2 3440 interferon alpha 2 IFN-alpha-2|IFN-alphaA|IFNA|IFNA2B|leIF A 9 Interferons

IFNA21 3452 interferon alpha 21 IFN-alphaI|LeIF F|leIF-F 9 Interferons

IFNA4 3441 interferon alpha 4 IFN-alpha4a|INFA4 9 Interferons

IFNA5 3442 interferon alpha 5 IFN-alpha-5|IFN-alphaG|INA5|INFA5|leIF G 9 Interferons

IFNA6 3443 interferon alpha 6 IFN-alphaK 9 Interferons

IFNA7 3444 interferon alpha 7 IFN-alphaJ|IFNA-J 9 Interferons

IFNA8 3445 interferon alpha 8 IFN-alphaB 9 Interferons

IFNB1 3456 interferon beta 1 IFB|IFF|IFN-beta|IFNB 9 Interferons

IFNE 338376 interferon epsilon IFN-E|IFNE1|IFNT1|INFE1|PRO655 9 Interferons

IFNG 3458 interferon gamma IFG|IFI 12 Interferons

IFNK 56832 interferon kappa IFNT1|INFE1 9 Interferons

IFNW1 3467 interferon omega 1 - 9 Interferons

IFNAR2 3455 interferon alpha and beta receptor subunit 2 IFN-R|IFN-alpha-REC|IFNABR|IFNARB|IMD45 21 Interferon_Receptor

IFNGR1 3459 interferon gamma receptor 1 CD119|IFNGR|IMD27A|IMD27B 6 Interferon_Receptor

IFNGR2 3460 interferon gamma receptor 2 AF-1|IFGR2|IFNGT1|IMD28 21 Interferon_Receptor

IL11 3589 interleukin 11 AGIF|IL-11 19 Interleukins

IL12A 3592 interleukin 12A CLMF|IL-12A|NFSK|NKSF1|P35 3 Interleukins

IL12B 3593 interleukin 12B CLMF|CLMF2|IL-12B|IMD28|IMD29|NKSF|NKSF2 5 Interleukins

IL13 3596 interleukin 13 IL-13|P600 5 Interleukins

IL15 3600 interleukin 15 IL-15 4 Interleukins

IL16 3603 interleukin 16 LCF|NIL16|PRIL16|prIL-16 15 Interleukins

IL17A 3605 interleukin 17A CTLA-8|CTLA8|IL-17|IL-17A|IL17 6 Interleukins

IL17B 27190 interleukin 17B IL-17B|IL-20|NIRF|ZCYTO7 5 Interleukins

IL17C 27189 interleukin 17C CX2|IL-17C 16 Interleukins

IL17D 53342 interleukin 17D IL-17D 13 Interleukins

IL17F 112744 interleukin 17F CANDF6|IL-17F|ML-1|ML1 6 Interleukins

IL18 3606 interleukin 18 IGIF|IL-18|IL-1g|IL1F4 11 Interleukins

IL19 29949 interleukin 19 IL-10C|MDA1|NG.1|ZMDA1 1 Interleukins

IL1A 3552 interleukin 1 alpha IL-1 alpha|IL-1A|IL1|IL1-ALPHA|IL1F1 2 Interleukins

IL1B 3553 interleukin 1 beta IL-1|IL1-BETA|IL1F2|IL1beta 2 Interleukins

IL1F10 84639 interleukin 1 family member 10 FIL1-theta|FKSG75|IL-1HY2|IL-38|IL1-theta|IL1HY2 2 Interleukins

IL36RN 26525 interleukin 36 receptor antagonist FIL1|FIL1(DELTA)|FIL1D|IL-36Ra|IL1F5|IL1HY1|IL1L1|IL1RP3|IL36RA|PSORP|PSORS14 2 Interleukins

IL36A 27179 interleukin 36 alpha FIL1|FIL1(EPSILON)|FIL1E|IL-1F6|IL1(EPSILON)|IL1F6 2 Interleukins

IL37 27178 interleukin 37 FIL1|FIL1(ZETA)|FIL1Z|IL-1F7|IL-1H|IL-1H4|IL-1RP1|IL-37|IL1F7|IL1H4|IL1RP1 2 Interleukins

IL36B 27177 interleukin 36 beta FIL1|FIL1-(ETA)|FIL1H|FILI-(ETA)|IL-1F8|IL-1H2|IL1-ETA|IL1F8|IL1H2 2 Interleukins

IL36G 56300 interleukin 36 gamma IL-1F9|IL-1H1|IL-1RP2|IL1E|IL1F9|IL1H1|IL1RP2 2 Interleukins

IL1RN 3557 interleukin 1 receptor antagonist DIRA|ICIL-1RA|IL-1RN|IL-1ra|IL-1ra3|IL1F3|IL1RA|IRAP|MVCD4 2 Interleukins

IL2 3558 interleukin 2 IL-2|TCGF|lymphokine 4 Interleukins

IL20 50604 interleukin 20 IL-20|IL10D|ZCYTO10 1 Interleukins

IL21 59067 interleukin 21 CVID11|IL-21|Za11 4 Interleukins

IL22 50616 interleukin 22 IL-21|IL-22|IL-D110|IL-TIF|ILTIF|TIFIL-23|TIFa|zcyto18 12 Interleukins

IL23A 51561 interleukin 23 subunit alpha IL-23|IL-23A|IL23P19|P19|SGRF 12 Interleukins

IL24 11009 interleukin 24 C49A|FISP|IL10B|MDA7|MOB5|ST16 1 Interleukins

IL25 64806 interleukin 25 IL17E 14 Interleukins

IL26 55801 interleukin 26 AK155|IL-26 12 Interleukins

IL27 246778 interleukin 27 IL-27|IL-27A|IL27A|IL27p28|IL30|p28 16 Interleukins

IFNL2 282616 interferon lambda 2 IL-28A|IL28A 19 Interleukins

IFNL3 282617 interferon lambda 3 IFN-lambda-3|IFN-lambda-4|IL-28B|IL-28C|IL28B|IL28C 19 Interleukins

IFNL1 282618 interferon lambda 1 IL-29|IL29 19 Interleukins

IL3 3562 interleukin 3 IL-3|MCGF|MULTI-CSF 5 Interleukins

IL31 386653 interleukin 31 IL-31 12 Interleukins

IL32 9235 interleukin 32 IL-32alpha|IL-32beta|IL-32delta|IL-32gamma|NK4|TAIF|TAIFa|TAIFb|TAIFc|TAIFd 16 Interleukins

IL33 90865 interleukin 33 C9orf26|DVS27|IL1F11|NF-HEV|NFEHEV 9 Interleukins

IL34 146433 interleukin 34 C16orf77|IL-34 16 Interleukins

IL4 3565 interleukin 4 BCGF-1|BCGF1|BSF-1|BSF1|IL-4 5 Interleukins

IL5 3567 interleukin 5 EDF|IL-5|TRF 5 Interleukins

IL6 3569 interleukin 6 BSF-2|BSF2|CDF|HGF|HSF|IFN-beta-2|IFNB2|IL-6 7 Interleukins

IL6ST 3572 interleukin 6 signal transducer CD130|CDW130|GP130|HIES4|IL-6RB|sGP130 5 Interleukins

IL7 3574 interleukin 7 IL-7 8 Interleukins

CXCL8 3576 C-X-C motif chemokine ligand 8 GCP-1|GCP1|IL8|LECT|LUCT|LYNAP|MDNCF|MONAP|NAF|NAP-1|NAP1|SCYB8 4 Interleukins

IL9 3578 interleukin 9 HP40|IL-9|P40 5 Interleukins

TXLNA 200081 taxilin alpha IL14|TXLN 1 Interleukins

IL10RA 3587 interleukin 10 receptor subunit alpha CD210|CD210a|CDW210A|HIL-10R|IL-10R1|IL10R 11 Interleukins_Receptor

IL10RB 3588 interleukin 10 receptor subunit beta CDW210B|CRF2-4|CRFB4|D21S58|D21S66|IL-10R2 21 Interleukins_Receptor

IL11RA 3590 interleukin 11 receptor subunit alpha CRSDA 9 Interleukins_Receptor

IL12RB1 3594 interleukin 12 receptor subunit beta 1 CD212|IL-12R-BETA1|IL12RB|IMD30 19 Interleukins_Receptor

IL12RB2 3595 interleukin 12 receptor subunit beta 2 - 1 Interleukins_Receptor

IL13RA1 3597 interleukin 13 receptor subunit alpha 1 CD213A1|CT19|IL-13Ra|NR4 X Interleukins_Receptor

IL13RA2 3598 interleukin 13 receptor subunit alpha 2 CD213A2|CT19|IL-13R|IL13BP X Interleukins_Receptor

IL15RA 3601 interleukin 15 receptor subunit alpha CD215 10 Interleukins_Receptor

IL2RB 3560 interleukin 2 receptor subunit beta CD122|IL15RB|IMD63|P70-75 22 Interleukins_Receptor

IL17RA 23765 interleukin 17 receptor A CANDF5|CD217|CDw217|IL-17RA|IL17R|IMD51|hIL-17R 22 Interleukins_Receptor

IL17RB 55540 interleukin 17 receptor B CRL4|EVI27|IL17BR|IL17RH1 3 Interleukins_Receptor

IL17RC 84818 interleukin 17 receptor C CANDF9|IL17-RL|IL17RL 3 Interleukins_Receptor

IL17RD 54756 interleukin 17 receptor D HH18|IL-17RD|IL17RLM|SEF 3 Interleukins_Receptor

IL17RE 132014 interleukin 17 receptor E - 3 Interleukins_Receptor

IL18R1 8809 interleukin 18 receptor 1 CD218a|CDw218a|IL-18R-alpha|IL-18Ralpha|IL-1Rrp|IL18RA|IL18Ralpha2|IL1RRP 2 Interleukins_Receptor

IL18RAP 8807 interleukin 18 receptor accessory protein ACPL|CD218b|CDw218b|IL-18R-beta|IL-18RAcP|IL-18Rbeta|IL-1R-7|IL-1R7|IL-1RAcPL|IL18RB 2 Interleukins_Receptor

IL1R1 3554 interleukin 1 receptor type 1 CD121A|D2S1473|IL-1R-alpha|IL1R|IL1RA|P80 2 Interleukins_Receptor

IL1R2 7850 interleukin 1 receptor type 2 CD121b|CDw121b|IL-1R-2|IL-1RT-2|IL-1RT2|IL1R2c|IL1RB 2 Interleukins_Receptor

IL1RAP 3556 interleukin 1 receptor accessory protein C3orf13|IL-1RAcP|IL1R3 3 Interleukins_Receptor

IL1RL1 9173 interleukin 1 receptor like 1 DER4|FIT-1|IL33R|ST2|ST2L|ST2V|T1 2 Interleukins_Receptor

IL1RL2 8808 interleukin 1 receptor like 2 IL-1Rrp2|IL-36R|IL1R-rp2|IL1RRP2 2 Interleukins_Receptor

IL20RA 53832 interleukin 20 receptor subunit alpha CRF2-8|IL-20R-alpha|IL-20R1|IL-20RA 6 Interleukins_Receptor

IL20RB 53833 interleukin 20 receptor subunit beta DIRS1|FNDC6|IL-20R2 3 Interleukins_Receptor

IL21R 50615 interleukin 21 receptor CD360|IMD56|NILR 16 Interleukins_Receptor

IL22RA1 58985 interleukin 22 receptor subunit alpha 1 CRF2-9|IL22R|IL22R1 1 Interleukins_Receptor

IL22RA2 116379 interleukin 22 receptor subunit alpha 2 CRF2-10|CRF2-S1|CRF2X|IL-22BP|IL-22R-alpha-2|IL-22RA2|ZCYTOR16 6 Interleukins_Receptor

IL23R 149233 interleukin 23 receptor - 1 Interleukins_Receptor

IL27RA 9466 interleukin 27 receptor subunit alpha CRL1|IL-27RA|IL27R|TCCR|WSX1|zcytor1 19 Interleukins_Receptor

IFNLR1 163702 interferon lambda receptor 1 CRF2/12|IFNLR|IL-28R1|IL28RA|LICR2 1 Interleukins_Receptor

IL2RA 3559 interleukin 2 receptor subunit alpha CD25|IDDM10|IL2R|IMD41|TCGFR|p55 10 Interleukins_Receptor

IL2RB 3560 interleukin 2 receptor subunit beta CD122|IL15RB|IMD63|P70-75 22 Interleukins_Receptor

IL2RG 3561 interleukin 2 receptor subunit gamma CD132|CIDX|IL-2RG|IMD4|P64|SCIDX|SCIDX1 X Interleukins_Receptor

IL31RA 133396 interleukin 31 receptor A CRL|CRL3|GLM-R|GLMR|GPL|IL-31RA|PLCA2|PRO21384|hGLM-R 5 Interleukins_Receptor

IL3RA 3563 interleukin 3 receptor subunit alpha CD123|IL3R|IL3RAY|IL3RX|IL3RY|hIL-3Ra X|Y Interleukins_Receptor

IL4R 3566 interleukin 4 receptor CD124|IL-4RA|IL4RA 16 Interleukins_Receptor

IL5RA 3568 interleukin 5 receptor subunit alpha CD125|CDw125|HSIL5R3|IL5R 3 Interleukins_Receptor

IL6R 3570 interleukin 6 receptor CD126|IL-6R-1|IL-6RA|IL6Q|IL6RA|IL6RQ|gp80 1 Interleukins_Receptor

IL7R 3575 interleukin 7 receptor CD127|CDW127|IL-7R-alpha|IL7RA|ILRA 5 Interleukins_Receptor

CXCR1 3577 C-X-C motif chemokine receptor 1 C-C|C-C-CKR-1|CD128|CD181|CDw128a|CKR-1|CMKAR1|IL8R1|IL8RA|IL8RBA 2 Interleukins_Receptor

CXCR2 3579 C-X-C motif chemokine receptor 2 CD182|CDw128b|CMKAR2|IL8R2|IL8RA|IL8RB 2 Interleukins_Receptor

IL9R 3581 interleukin 9 receptor CD129|IL-9R X|Y Interleukins_Receptor

ST2 6761 - - 11 Interleukins_Receptor

HLA-A 3105 major histocompatibility complex, class I, A HLAA 6 NaturalKiller_Cell_Cytotoxicity

HLA-B 3106 major histocompatibility complex, class I, B AS|B-4901|HLAB 6 NaturalKiller_Cell_Cytotoxicity

HLA-C 3107 major histocompatibility complex, class I, C D6S204|HLA-JY3|HLAC|HLC-C|MHC|PSORS1 6 NaturalKiller_Cell_Cytotoxicity

HLA-E 3133 major histocompatibility complex, class I, E HLA-6.2|QA1 6 NaturalKiller_Cell_Cytotoxicity

HLA-G 3135 major histocompatibility complex, class I, G MHC-G 6 NaturalKiller_Cell_Cytotoxicity

KIR3DL1 3811 killer cell immunoglobulin like receptor, three Ig domains and long cytoplasmic tail 1 CD158E1|KIR|KIR3DL1/S1|NKAT-3|NKAT3|NKB1|NKB1B 19 NaturalKiller_Cell_Cytotoxicity

KIR3DL2 3812 killer cell immunoglobulin like receptor, three Ig domains and long cytoplasmic tail 2 3DL2|CD158K|KIR-3DL2|NKAT-4|NKAT4|NKAT4B|p140 19 NaturalKiller_Cell_Cytotoxicity

KIR2DL1 3802 killer cell immunoglobulin like receptor, two Ig domains and long cytoplasmic tail 1 CD158A|KIR-K64|KIR221|KIR2DL3|NKAT|NKAT-1|NKAT1|p58.1 19 NaturalKiller_Cell_Cytotoxicity

KIR2DL2 3803 killer cell immunoglobulin like receptor, two Ig domains and long cytoplasmic tail 2 CD158B1|CD158b|NKAT-6|NKAT6|p58.2 19 NaturalKiller_Cell_Cytotoxicity

KIR2DL3 3804 killer cell immunoglobulin like receptor, two Ig domains and long cytoplasmic tail 3 CD158B2|CD158b|GL183|KIR-023GB|KIR-K7b|KIR-K7c|KIR2DL|KIR2DS5|KIRCL23|NKAT|NKAT2|NKAT2A|NKAT2B|p58 19 NaturalKiller_Cell_Cytotoxicity

KIR2DL4 3805 killer cell immunoglobulin like receptor, two Ig domains and long cytoplasmic tail 4 CD158D|G9P|KIR-103AS|KIR-2DL4|KIR103|KIR103AS 19 NaturalKiller_Cell_Cytotoxicity

KIR2DL5A 57292 killer cell immunoglobulin like receptor, two Ig domains and long cytoplasmic tail 5A CD158F|KIR2DL5|KIR2DL5.1|KIR2DL5.3 19 NaturalKiller_Cell_Cytotoxicity

KLRC1 3821 killer cell lectin like receptor C1 CD159A|NKG2|NKG2A 12 NaturalKiller_Cell_Cytotoxicity

KLRC2 3822 killer cell lectin like receptor C2 CD159c|NKG2-C|NKG2C 12 NaturalKiller_Cell_Cytotoxicity

KLRC3 3823 killer cell lectin like receptor C3 NKG2-E|NKG2E 12 NaturalKiller_Cell_Cytotoxicity

KLRD1 3824 killer cell lectin like receptor D1 CD94 12 NaturalKiller_Cell_Cytotoxicity

PTPN6 5777 protein tyrosine phosphatase non-receptor type 6 HCP|HCPH|HPTP1C|PTP-1C|SH-PTP1|SHP-1|SHP-1L|SHP1 12 NaturalKiller_Cell_Cytotoxicity

PTPN11 5781 protein tyrosine phosphatase non-receptor type 11 BPTP3|CFC|JMML|METCDS|NS1|PTP-1D|PTP2C|SH-PTP2|SH-PTP3|SHP2 12 NaturalKiller_Cell_Cytotoxicity

ICAM1 3383 intercellular adhesion molecule 1 BB2|CD54|P3.58 19 NaturalKiller_Cell_Cytotoxicity

ICAM2 3384 intercellular adhesion molecule 2 CD102 17 NaturalKiller_Cell_Cytotoxicity

ITGAL 3683 integrin subunit alpha L CD11A|LFA-1|LFA1A 16 NaturalKiller_Cell_Cytotoxicity

ITGB2 3689 integrin subunit beta 2 CD18|LAD|LCAMB|LFA-1|MAC-1|MF17|MFI7 21 NaturalKiller_Cell_Cytotoxicity

PTK2B 2185 protein tyrosine kinase 2 beta CADTK|CAKB|FADK2|FAK2|PKB|PTK|PYK2|RAFTK 8 NaturalKiller_Cell_Cytotoxicity

VAV3 10451 vav guanine nucleotide exchange factor 3 - 1 NaturalKiller_Cell_Cytotoxicity

VAV1 7409 vav guanine nucleotide exchange factor 1 VAV 19 NaturalKiller_Cell_Cytotoxicity

VAV2 7410 vav guanine nucleotide exchange factor 2 VAV-2 9 NaturalKiller_Cell_Cytotoxicity

RAC1 5879 Rac family small GTPase 1 MIG5|MRD48|Rac-1|TC-25|p21-Rac1 7 NaturalKiller_Cell_Cytotoxicity

RAC2 5880 Rac family small GTPase 2 EN-7|Gx|HSPC022|p21-Rac2 22 NaturalKiller_Cell_Cytotoxicity

RAC3 5881 Rac family small GTPase 3 - 17 NaturalKiller_Cell_Cytotoxicity

PAK1 5058 p21 (RAC1) activated kinase 1 IDDMSSD|PAKalpha|alpha-PAK|p65-PAK 11 NaturalKiller_Cell_Cytotoxicity

MAP2K1 5604 mitogen-activated protein kinase kinase 1 CFC3|MAPKK1|MEK1|MKK1|PRKMK1 15 NaturalKiller_Cell_Cytotoxicity

MAP2K2 5605 mitogen-activated protein kinase kinase 2 CFC4|MAPKK2|MEK2|MKK2|PRKMK2 19 NaturalKiller_Cell_Cytotoxicity

MAPK1 5594 mitogen-activated protein kinase 1 ERK|ERK-2|ERK2|ERT1|MAPK2|P42MAPK|PRKM1|PRKM2|p38|p40|p41|p41mapk|p42-MAPK 22 NaturalKiller_Cell_Cytotoxicity

MAPK3 5595 mitogen-activated protein kinase 3 ERK-1|ERK1|ERT2|HS44KDAP|HUMKER1A|P44ERK1|P44MAPK|PRKM3|p44-ERK1|p44-MAPK 16 NaturalKiller_Cell_Cytotoxicity

TNF 7124 tumor necrosis factor DIF|TNF-alpha|TNFA|TNFSF2|TNLG1F 6 NaturalKiller_Cell_Cytotoxicity

CSF2 1437 colony stimulating factor 2 CSF|GMCSF 5 NaturalKiller_Cell_Cytotoxicity

IFNG 3458 interferon gamma IFG|IFI 12 NaturalKiller_Cell_Cytotoxicity

KIR2DS1 3806 killer cell immunoglobulin like receptor, two Ig domains and short cytoplasmic tail 1 CD158H|CD158a|p50.1 19 NaturalKiller_Cell_Cytotoxicity

KIR2DS3 3808 killer cell immunoglobulin like receptor, two Ig domains and short cytoplasmic tail 3 NKAT7 19 NaturalKiller_Cell_Cytotoxicity

KIR2DS4 3809 killer cell immunoglobulin like receptor, two Ig domains and short cytoplasmic tail 4 CD158I|KIR-2DS4|KIR1D|KIR412|KKA3|NKAT-8|NKAT8 19 NaturalKiller_Cell_Cytotoxicity

KIR2DS5 3810 killer cell immunoglobulin like receptor, two Ig domains and short cytoplasmic tail 5 CD158G|NKAT9 19 NaturalKiller_Cell_Cytotoxicity

NCR2 9436 natural cytotoxicity triggering receptor 2 CD336|LY95|NK-p44|NKP44|dJ149M18.1 6 NaturalKiller_Cell_Cytotoxicity

TYROBP 7305 transmembrane immune signaling adaptor TYROBP DAP12|KARAP|PLOSL|PLOSL1 19 NaturalKiller_Cell_Cytotoxicity

LCK 3932 LCK proto-oncogene, Src family tyrosine kinase IMD22|LSK|YT16|p56lck|pp58lck 1 NaturalKiller_Cell_Cytotoxicity

FCGR3A 2214 Fc fragment of IgG receptor IIIa CD16|CD16A|FCG3|FCGR3|FCGRIII|FCR-10|FCRIII|FCRIIIA|IGFR3|IMD20 1 NaturalKiller_Cell_Cytotoxicity

FCGR3B 2215 Fc fragment of IgG receptor IIIb CD16|CD16A|CD16b|FCG3|FCGR3|FCGR3A|FCR-10|FCRIII|FCRIIIb 1 NaturalKiller_Cell_Cytotoxicity

NCR1 9437 natural cytotoxicity triggering receptor 1 CD335|LY94|NK-p46|NKP46 19 NaturalKiller_Cell_Cytotoxicity

NCR3 259197 natural cytotoxicity triggering receptor 3 1C7|CD337|LY117|MALS|NKp30 6 NaturalKiller_Cell_Cytotoxicity

FCER1G 2207 Fc fragment of IgE receptor Ig FCRG 1 NaturalKiller_Cell_Cytotoxicity

CD247 919 CD247 molecule CD3-ZETA|CD3H|CD3Q|CD3Z|IMD25|T3Z|TCRZ 1 NaturalKiller_Cell_Cytotoxicity

ZAP70 7535 zeta chain of T cell receptor associated protein kinase 70 ADMIO2|IMD48|SRK|STCD|STD|TZK|ZAP-70 2 NaturalKiller_Cell_Cytotoxicity

SYK 6850 spleen associated tyrosine kinase p72-Syk 9 NaturalKiller_Cell_Cytotoxicity

LCP2 3937 lymphocyte cytosolic protein 2 SLP-76|SLP76 5 NaturalKiller_Cell_Cytotoxicity

LAT 27040 linker for activation of T cells IMD52|LAT1|pp36 16 NaturalKiller_Cell_Cytotoxicity

PLCG1 5335 phospholipase C gamma 1 NCKAP3|PLC-II|PLC1|PLC148|PLCgamma1 20 NaturalKiller_Cell_Cytotoxicity

PLCG2 5336 phospholipase C gamma 2 APLAID|FCAS3|PLC-IV|PLC-gamma-2 16 NaturalKiller_Cell_Cytotoxicity

SH3BP2 6452 SH3 domain binding protein 2 3BP-2|3BP2|CRBM|CRPM|RES4-23 4 NaturalKiller_Cell_Cytotoxicity

PIK3CA 5290 phosphatidylinositol-4,5-bisphosphate 3-kinase catalytic subunit alpha CLAPO|CLOVE|CWS5|MCAP|MCM|MCMTC|PI3K|PI3K-alpha|p110-alpha 3 NaturalKiller_Cell_Cytotoxicity

PIK3CB 5291 phosphatidylinositol-4,5-bisphosphate 3-kinase catalytic subunit beta P110BETA|PI3K|PI3KBETA|PIK3C1 3 NaturalKiller_Cell_Cytotoxicity

PIK3CD 5293 phosphatidylinositol-4,5-bisphosphate 3-kinase catalytic subunit delta APDS|IMD14|P110DELTA|PI3K|p110D 1 NaturalKiller_Cell_Cytotoxicity

PIK3CG 5294 phosphatidylinositol-4,5-bisphosphate 3-kinase catalytic subunit gamma PI3CG|PI3K|PI3Kgamma|PIK3|p110gamma|p120-PI3K 7 NaturalKiller_Cell_Cytotoxicity

PIK3R5 23533 phosphoinositide-3-kinase regulatory subunit 5 F730038I15Rik|FOAP-2|P101-PI3K|p101 17 NaturalKiller_Cell_Cytotoxicity

PIK3R1 5295 phosphoinositide-3-kinase regulatory subunit 1 AGM7|GRB1|IMD36|p85|p85-ALPHA 5 NaturalKiller_Cell_Cytotoxicity

PIK3R2 5296 phosphoinositide-3-kinase regulatory subunit 2 MPPH|MPPH1|P85B|p85|p85-BETA 19 NaturalKiller_Cell_Cytotoxicity

PIK3R3 8503 phosphoinositide-3-kinase regulatory subunit 3 p55|p55-GAMMA|p55PIK 1 NaturalKiller_Cell_Cytotoxicity

FYN 2534 FYN proto-oncogene, Src family tyrosine kinase SLK|SYN|p59-FYN 6 NaturalKiller_Cell_Cytotoxicity

SHC2 25759 SHC adaptor protein 2 SCK|SHCB|SLI 19 NaturalKiller_Cell_Cytotoxicity

SHC4 399694 SHC adaptor protein 4 RaLP|SHCD 15 NaturalKiller_Cell_Cytotoxicity

SHC3 53358 SHC adaptor protein 3 N-Shc|NSHC|RAI|SHCC 9 NaturalKiller_Cell_Cytotoxicity

SHC1 6464 SHC adaptor protein 1 SHC|SHCA 1 NaturalKiller_Cell_Cytotoxicity

GRB2 2885 growth factor receptor bound protein 2 ASH|EGFRBP-GRB2|Grb3-3|MST084|MSTP084|NCKAP2 17 NaturalKiller_Cell_Cytotoxicity

SOS1 6654 SOS Ras/Rac guanine nucleotide exchange factor 1 GF1|GGF1|GINGF|HGF|NS4|SOS-1 2 NaturalKiller_Cell_Cytotoxicity

SOS2 6655 SOS Ras/Rho guanine nucleotide exchange factor 2 NS9|SOS-2 14 NaturalKiller_Cell_Cytotoxicity

HRAS 3265 HRas proto-oncogene, GTPase C-BAS/HAS|C-H-RAS|C-HA-RAS1|CTLO|H-RASIDX|HAMSV|HRAS1|RASH1|p21ras 11 NaturalKiller_Cell_Cytotoxicity

KRAS 3845 KRAS proto-oncogene, GTPase 'C-K-RAS|C-K-RAS|CFC2|K-RAS2A|K-RAS2B|K-RAS4A|K-RAS4B|K-Ras|K-Ras 2|KI-RAS|KRAS1|KRAS2|NS|NS3|OES|RALD|RASK2|c-Ki-ras|c-Ki-ras2 12 NaturalKiller_Cell_Cytotoxicity

NRAS 4893 NRAS proto-oncogene, GTPase ALPS4|CMNS|N-ras|NCMS|NRAS1|NS6 1 NaturalKiller_Cell_Cytotoxicity

ARAF 369 A-Raf proto-oncogene, serine/threonine kinase A-RAF|ARAF1|PKS2|RAFA1 X NaturalKiller_Cell_Cytotoxicity

BRAF 673 B-Raf proto-oncogene, serine/threonine kinase B-RAF1|B-raf|BRAF1|NS7|RAFB1 7 NaturalKiller_Cell_Cytotoxicity

RAF1 5894 Raf-1 proto-oncogene, serine/threonine kinase CMD1NN|CRAF|NS5|Raf-1|c-Raf 3 NaturalKiller_Cell_Cytotoxicity

MICA 100507436 MHC class I polypeptide-related sequence A MIC-A|PERB11.1 6 NaturalKiller_Cell_Cytotoxicity

MICB 4277 MHC class I polypeptide-related sequence B PERB11.2 6 NaturalKiller_Cell_Cytotoxicity

ULBP3 79465 UL16 binding protein 3 N2DL-3|NKG2DL3|RAET1N 6 NaturalKiller_Cell_Cytotoxicity

ULBP2 80328 UL16 binding protein 2 ALCAN-alpha|N2DL2|NKG2DL2|RAET1H|RAET1L 6 NaturalKiller_Cell_Cytotoxicity

ULBP1 80329 UL16 binding protein 1 N2DL-1|NKG2DL1|RAET1I 6 NaturalKiller_Cell_Cytotoxicity

KLRK1 22914 killer cell lectin like receptor K1 CD314|D12S2489E|KLR|NKG2-D|NKG2D 12 NaturalKiller_Cell_Cytotoxicity

HCST 10870 hematopoietic cell signal transducer DAP10|KAP10|PIK3AP 19 NaturalKiller_Cell_Cytotoxicity

CD48 962 CD48 molecule BCM1|BLAST|BLAST1|MEM-102|SLAMF2|hCD48|mCD48 1 NaturalKiller_Cell_Cytotoxicity

CD244 51744 CD244 molecule 2B4|NAIL|NKR2B4|Nmrk|SLAMF4 1 NaturalKiller_Cell_Cytotoxicity

PPP3CA 5530 protein phosphatase 3 catalytic subunit alpha ACCIID|CALN|CALNA|CALNA1|CCN1|CNA1|IECEE|IECEE1|PPP2B 4 NaturalKiller_Cell_Cytotoxicity

PPP3CB 5532 protein phosphatase 3 catalytic subunit beta CALNA2|CALNB|CNA2|PP2Bbeta 10 NaturalKiller_Cell_Cytotoxicity

PPP3CC 5533 protein phosphatase 3 catalytic subunit gamma CALNA3|CNA3|PP2Bgamma 8 NaturalKiller_Cell_Cytotoxicity

CHP1 11261 calcineurin like EF-hand protein 1 CHP|SLC9A1BP|SPAX9|Sid470p|p22|p24 15 NaturalKiller_Cell_Cytotoxicity

PPP3R1 5534 protein phosphatase 3 regulatory subunit B, alpha CALNB1|CNB|CNB1 2 NaturalKiller_Cell_Cytotoxicity

PPP3R2 5535 protein phosphatase 3 regulatory subunit B, beta PPP3RL 9 NaturalKiller_Cell_Cytotoxicity

CHP2 63928 calcineurin like EF-hand protein 2 - 16 NaturalKiller_Cell_Cytotoxicity

NFAT5 10725 nuclear factor of activated T cells 5 NF-AT5|NFATL1|NFATZ|OREBP|TONEBP 16 NaturalKiller_Cell_Cytotoxicity

NFATC1 4772 nuclear factor of activated T cells 1 NF-ATC|NF-ATc1.2|NFAT2|NFATc 18 NaturalKiller_Cell_Cytotoxicity

NFATC2 4773 nuclear factor of activated T cells 2 NFAT1|NFATP 20 NaturalKiller_Cell_Cytotoxicity

NFATC3 4775 nuclear factor of activated T cells 3 NF-AT4c|NFAT4|NFATX 16 NaturalKiller_Cell_Cytotoxicity

NFATC4 4776 nuclear factor of activated T cells 4 NF-AT3|NF-ATC4|NFAT3 14 NaturalKiller_Cell_Cytotoxicity

PRKCA 5578 protein kinase C alpha AAG6|PKC-alpha|PKCA|PKCI+/-|PKCalpha|PRKACA 17 NaturalKiller_Cell_Cytotoxicity

PRKCB 5579 protein kinase C beta PKC-beta|PKCB|PKCI(2)|PKCbeta|PRKCB1|PRKCB2 16 NaturalKiller_Cell_Cytotoxicity

PRKCG 5582 protein kinase C gamma PKC-gamma|PKCC|PKCG|PKCI(3)|PKCgamma|SCA14 19 NaturalKiller_Cell_Cytotoxicity

SH2D1B 117157 SH2 domain containing 1B EAT2 1 NaturalKiller_Cell_Cytotoxicity

SH2D1A 4068 SH2 domain containing 1A DSHP|EBVS|IMD5|LYP|MTCP1|SAP|SAP/SH2D1A|XLP|XLPD|XLPD1 X NaturalKiller_Cell_Cytotoxicity

IFNGR1 3459 interferon gamma receptor 1 CD119|IFNGR|IMD27A|IMD27B 6 NaturalKiller_Cell_Cytotoxicity

IFNGR2 3460 interferon gamma receptor 2 AF-1|IFGR2|IFNGT1|IMD28 21 NaturalKiller_Cell_Cytotoxicity

IFNA1 3439 interferon alpha 1 IFL|IFN|IFN-ALPHA|IFN-alphaD|IFNA13|IFNA@|leIF D 9 NaturalKiller_Cell_Cytotoxicity

IFNA2 3440 interferon alpha 2 IFN-alpha-2|IFN-alphaA|IFNA|IFNA2B|leIF A 9 NaturalKiller_Cell_Cytotoxicity

IFNA4 3441 interferon alpha 4 IFN-alpha4a|INFA4 9 NaturalKiller_Cell_Cytotoxicity

IFNA5 3442 interferon alpha 5 IFN-alpha-5|IFN-alphaG|INA5|INFA5|leIF G 9 NaturalKiller_Cell_Cytotoxicity

IFNA6 3443 interferon alpha 6 IFN-alphaK 9 NaturalKiller_Cell_Cytotoxicity

IFNA7 3444 interferon alpha 7 IFN-alphaJ|IFNA-J 9 NaturalKiller_Cell_Cytotoxicity

IFNA8 3445 interferon alpha 8 IFN-alphaB 9 NaturalKiller_Cell_Cytotoxicity

IFNA10 3446 interferon alpha 10 IFN-alphaC 9 NaturalKiller_Cell_Cytotoxicity

IFNA13 3447 interferon alpha 13 - 9 NaturalKiller_Cell_Cytotoxicity

IFNA14 3448 interferon alpha 14 IFN-alphaH|LEIF2H 9 NaturalKiller_Cell_Cytotoxicity

IFNA16 3449 interferon alpha 16 IFN-alpha-16|IFN-alphaO 9 NaturalKiller_Cell_Cytotoxicity

IFNA17 3451 interferon alpha 17 IFN-alphaI|IFNA|INFA|LEIF2C1 9 NaturalKiller_Cell_Cytotoxicity

IFNA21 3452 interferon alpha 21 IFN-alphaI|LeIF F|leIF-F 9 NaturalKiller_Cell_Cytotoxicity

IFNB1 3456 interferon beta 1 IFB|IFF|IFN-beta|IFNB 9 NaturalKiller_Cell_Cytotoxicity

IFNAR1 3454 interferon alpha and beta receptor subunit 1 AVP|IFN-alpha-REC|IFNAR|IFNBR|IFRC 21 NaturalKiller_Cell_Cytotoxicity

IFNAR2 3455 interferon alpha and beta receptor subunit 2 IFN-R|IFN-alpha-REC|IFNABR|IFNARB|IMD45 21 NaturalKiller_Cell_Cytotoxicity

TNFSF10 8743 TNF superfamily member 10 APO2L|Apo-2L|CD253|TL2|TNLG6A|TRAIL 3 NaturalKiller_Cell_Cytotoxicity

TNFRSF10D 8793 TNF receptor superfamily member 10d CD264|DCR2|TRAIL-R4|TRAILR4|TRUNDD 8 NaturalKiller_Cell_Cytotoxicity

TNFRSF10C 8794 TNF receptor superfamily member 10c CD263|DCR1|DCR1-TNFR|LIT|TRAIL-R3|TRAILR3|TRID 8 NaturalKiller_Cell_Cytotoxicity

TNFRSF10B 8795 TNF receptor superfamily member 10b CD262|DR5|KILLER|KILLER/DR5|TRAIL-R2|TRAILR2|TRICK2|TRICK2A|TRICK2B|TRICKB|ZTNFR9 8 NaturalKiller_Cell_Cytotoxicity

TNFRSF10A 8797 TNF receptor superfamily member 10a APO2|CD261|DR4|TRAILR-1|TRAILR1 8 NaturalKiller_Cell_Cytotoxicity

FASLG 356 Fas ligand ALPS1B|APT1LG1|APTL|CD178|CD95-L|CD95L|FASL|TNFSF6|TNLG1A 1 NaturalKiller_Cell_Cytotoxicity

FAS 355 Fas cell surface death receptor ALPS1A|APO-1|APT1|CD95|FAS1|FASTM|TNFRSF6 10 NaturalKiller_Cell_Cytotoxicity

GZMB 3002 granzyme B C11|CCPI|CGL-1|CGL1|CSP-B|CSPB|CTLA1|CTSGL1|HLP|SECT 14 NaturalKiller_Cell_Cytotoxicity

PRF1 5551 perforin 1 HPLH2|P1|PFP 10 NaturalKiller_Cell_Cytotoxicity

CASP3 836 caspase 3 CPP32|CPP32B|SCA-1 4 NaturalKiller_Cell_Cytotoxicity

BID 637 BH3 interacting domain death agonist FP497 22 NaturalKiller_Cell_Cytotoxicity

CD3D 915 CD3d molecule CD3-DELTA|IMD19|T3D 11 TCRsignalingPathway

CD3E 916 CD3e molecule IMD18|T3E|TCRE 11 TCRsignalingPathway

CD3G 917 CD3g molecule CD3-GAMMA|IMD17|T3G 11 TCRsignalingPathway

CD247 919 CD247 molecule CD3-ZETA|CD3H|CD3Q|CD3Z|IMD25|T3Z|TCRZ 1 TCRsignalingPathway

CD4 920 CD4 molecule CD4mut 12 TCRsignalingPathway

CD8A 925 CD8a molecule CD8|Leu2|p32 2 TCRsignalingPathway

CD8B 926 CD8b molecule CD8B1|LEU2|LY3|LYT3|P37 2 TCRsignalingPathway

PTPRC 5788 protein tyrosine phosphatase receptor type C B220|CD45|CD45R|GP180|L-CA|LCA|LY5|T200 1 TCRsignalingPathway

LCK 3932 LCK proto-oncogene, Src family tyrosine kinase IMD22|LSK|YT16|p56lck|pp58lck 1 TCRsignalingPathway

FYN 2534 FYN proto-oncogene, Src family tyrosine kinase SLK|SYN|p59-FYN 6 TCRsignalingPathway

ZAP70 7535 zeta chain of T cell receptor associated protein kinase 70 ADMIO2|IMD48|SRK|STCD|STD|TZK|ZAP-70 2 TCRsignalingPathway

LCP2 3937 lymphocyte cytosolic protein 2 SLP-76|SLP76 5 TCRsignalingPathway

LAT 27040 linker for activation of T cells IMD52|LAT1|pp36 16 TCRsignalingPathway

ITK 3702 IL2 inducible T cell kinase EMT|LPFS1|LYK|PSCTK2 5 TCRsignalingPathway

TEC 7006 tec protein tyrosine kinase PSCTK4 4 TCRsignalingPathway

NCK1 4690 NCK adaptor protein 1 NCK|NCKalpha|nck-1 3 TCRsignalingPathway

NCK2 8440 NCK adaptor protein 2 GRB4|NCKbeta 2 TCRsignalingPathway

VAV3 10451 vav guanine nucleotide exchange factor 3 - 1 TCRsignalingPathway

VAV1 7409 vav guanine nucleotide exchange factor 1 VAV 19 TCRsignalingPathway

VAV2 7410 vav guanine nucleotide exchange factor 2 VAV-2 9 TCRsignalingPathway

GRAP2 9402 GRB2 related adaptor protein 2 GADS|GRAP-2|GRB2L|GRBLG|GRID|GRPL|GrbX|Grf40|Mona|P38 22 TCRsignalingPathway

GRB2 2885 growth factor receptor bound protein 2 ASH|EGFRBP-GRB2|Grb3-3|MST084|MSTP084|NCKAP2 17 TCRsignalingPathway

PAK1 5058 p21 (RAC1) activated kinase 1 IDDMSSD|PAKalpha|alpha-PAK|p65-PAK 11 TCRsignalingPathway

PAK2 5062 p21 (RAC1) activated kinase 2 PAK65|PAKgamma 3 TCRsignalingPathway

PAK3 5063 p21 (RAC1) activated kinase 3 ARA|MRX30|MRX47|OPHN3|PAK-3|PAK3beta|bPAK|beta-PAK X TCRsignalingPathway

PAK4 10298 p21 (RAC1) activated kinase 4 - 19 TCRsignalingPathway

PAK6 56924 p21 (RAC1) activated kinase 6 PAK5 15 TCRsignalingPathway

PAK5 57144 p21 (RAC1) activated kinase 5 PAK7 20 TCRsignalingPathway

RHOA 387 ras homolog family member A ARH12|ARHA|EDFAOB|RHO12|RHOH12 3 TCRsignalingPathway

CDC42 998 cell division cycle 42 CDC42Hs|G25K|TKS 1 TCRsignalingPathway

PPP3CA 5530 protein phosphatase 3 catalytic subunit alpha ACCIID|CALN|CALNA|CALNA1|CCN1|CNA1|IECEE|IECEE1|PPP2B 4 TCRsignalingPathway

PPP3CB 5532 protein phosphatase 3 catalytic subunit beta CALNA2|CALNB|CNA2|PP2Bbeta 10 TCRsignalingPathway

PPP3CC 5533 protein phosphatase 3 catalytic subunit gamma CALNA3|CNA3|PP2Bgamma 8 TCRsignalingPathway

CHP1 11261 calcineurin like EF-hand protein 1 CHP|SLC9A1BP|SPAX9|Sid470p|p22|p24 15 TCRsignalingPathway

PPP3R1 5534 protein phosphatase 3 regulatory subunit B, alpha CALNB1|CNB|CNB1 2 TCRsignalingPathway

PPP3R2 5535 protein phosphatase 3 regulatory subunit B, beta PPP3RL 9 TCRsignalingPathway

CHP2 63928 calcineurin like EF-hand protein 2 - 16 TCRsignalingPathway

NFAT5 10725 nuclear factor of activated T cells 5 NF-AT5|NFATL1|NFATZ|OREBP|TONEBP 16 TCRsignalingPathway

NFATC1 4772 nuclear factor of activated T cells 1 NF-ATC|NF-ATc1.2|NFAT2|NFATc 18 TCRsignalingPathway

NFATC2 4773 nuclear factor of activated T cells 2 NFAT1|NFATP 20 TCRsignalingPathway

NFATC3 4775 nuclear factor of activated T cells 3 NF-AT4c|NFAT4|NFATX 16 TCRsignalingPathway

NFATC4 4776 nuclear factor of activated T cells 4 NF-AT3|NF-ATC4|NFAT3 14 TCRsignalingPathway

SOS1 6654 SOS Ras/Rac guanine nucleotide exchange factor 1 GF1|GGF1|GINGF|HGF|NS4|SOS-1 2 TCRsignalingPathway

SOS2 6655 SOS Ras/Rho guanine nucleotide exchange factor 2 NS9|SOS-2 14 TCRsignalingPathway

HRAS 3265 HRas proto-oncogene, GTPase C-BAS/HAS|C-H-RAS|C-HA-RAS1|CTLO|H-RASIDX|HAMSV|HRAS1|RASH1|p21ras 11 TCRsignalingPathway

KRAS 3845 KRAS proto-oncogene, GTPase 'C-K-RAS|C-K-RAS|CFC2|K-RAS2A|K-RAS2B|K-RAS4A|K-RAS4B|K-Ras|K-Ras 2|KI-RAS|KRAS1|KRAS2|NS|NS3|OES|RALD|RASK2|c-Ki-ras|c-Ki-ras2 12 TCRsignalingPathway

NRAS 4893 NRAS proto-oncogene, GTPase ALPS4|CMNS|N-ras|NCMS|NRAS1|NS6 1 TCRsignalingPathway

FOS 2353 Fos proto-oncogene, AP-1 transcription factor subunit AP-1|C-FOS|p55 14 TCRsignalingPathway

JUN 3725 Jun proto-oncogene, AP-1 transcription factor subunit AP-1|AP1|c-Jun|cJUN|p39 1 TCRsignalingPathway

CARD11 84433 caspase recruitment domain family member 11 BENTA|BIMP3|CARMA1|IMD11|IMD11A|PPBL 7 TCRsignalingPathway

BCL10 8915 BCL10 immune signaling adaptor CARMEN|CIPER|CLAP|IMD37|c-E10|mE10 1 TCRsignalingPathway

MALT1 10892 MALT1 paracaspase IMD12|MLT|MLT1|PCASP1 18 TCRsignalingPathway

CHUK 1147 component of inhibitor of nuclear factor kappa B kinase complex IKBKA|IKK-alpha|IKK1|IKKA|NFKBIKA|TCF16 10 TCRsignalingPathway

IKBKB 3551 inhibitor of nuclear factor kappa B kinase subunit beta IKK-beta|IKK2|IKKB|IMD15|IMD15A|IMD15B|NFKBIKB 8 TCRsignalingPathway

IKBKG 8517 inhibitor of nuclear factor kappa B kinase regulatory subunit gamma AMCBX1|EDAID1|FIP-3|FIP3|Fip3p|IKK-gamma|IKKAP1|IKKG|IMD33|IP|IP1|IP2|IPD2|NEMO|ZC2HC9 X TCRsignalingPathway

NFKB1 4790 nuclear factor kappa B subunit 1 CVID12|EBP-1|KBF1|NF-kB|NF-kB1|NF-kappa-B1|NF-kappaB|NF-kappabeta|NFKB-p105|NFKB-p50|NFkappaB 4 TCRsignalingPathway

RELA 5970 RELA proto-oncogene, NF-kB subunit CMCU|NFKB3|p65 11 TCRsignalingPathway

NFKBIA 4792 NFKB inhibitor alpha EDAID2|IKBA|MAD-3|NFKBI 14 TCRsignalingPathway

NFKBIB 4793 NFKB inhibitor beta IKBB|TRIP9 19 TCRsignalingPathway

NFKBIE 4794 NFKB inhibitor epsilon IKBE 6 TCRsignalingPathway

CD28 940 CD28 molecule Tp44 2 TCRsignalingPathway

ICOS 29851 inducible T cell costimulator AILIM|CD278|CVID1 2 TCRsignalingPathway

CD40LG 959 CD40 ligand CD154|CD40L|HIGM1|IGM|IMD3|T-BAM|TNFSF5|TRAP|gp39|hCD40L X TCRsignalingPathway

PIK3R5 23533 phosphoinositide-3-kinase regulatory subunit 5 F730038I15Rik|FOAP-2|P101-PI3K|p101 17 TCRsignalingPathway

PIK3R1 5295 phosphoinositide-3-kinase regulatory subunit 1 AGM7|GRB1|IMD36|p85|p85-ALPHA 5 TCRsignalingPathway

PIK3R2 5296 phosphoinositide-3-kinase regulatory subunit 2 MPPH|MPPH1|P85B|p85|p85-BETA 19 TCRsignalingPathway

PIK3R3 8503 phosphoinositide-3-kinase regulatory subunit 3 p55|p55-GAMMA|p55PIK 1 TCRsignalingPathway

PIK3CA 5290 phosphatidylinositol-4,5-bisphosphate 3-kinase catalytic subunit alpha CLAPO|CLOVE|CWS5|MCAP|MCM|MCMTC|PI3K|PI3K-alpha|p110-alpha 3 TCRsignalingPathway

PIK3CB 5291 phosphatidylinositol-4,5-bisphosphate 3-kinase catalytic subunit beta P110BETA|PI3K|PI3KBETA|PIK3C1 3 TCRsignalingPathway

PIK3CD 5293 phosphatidylinositol-4,5-bisphosphate 3-kinase catalytic subunit delta APDS|IMD14|P110DELTA|PI3K|p110D 1 TCRsignalingPathway

PIK3CG 5294 phosphatidylinositol-4,5-bisphosphate 3-kinase catalytic subunit gamma PI3CG|PI3K|PI3Kgamma|PIK3|p110gamma|p120-PI3K 7 TCRsignalingPathway

AKT3 10000 AKT serine/threonine kinase 3 MPPH|MPPH2|PKB-GAMMA|PKBG|PRKBG|RAC-PK-gamma|RAC-gamma|STK-2 1 TCRsignalingPathway

AKT1 207 AKT serine/threonine kinase 1 AKT|CWS6|PKB|PKB-ALPHA|PRKBA|RAC|RAC-ALPHA 14 TCRsignalingPathway

AKT2 208 AKT serine/threonine kinase 2 HIHGHH|PKBB|PKBBETA|PRKBB|RAC-BETA 19 TCRsignalingPathway

MAP3K8 1326 mitogen-activated protein kinase kinase kinase 8 AURA2|COT|EST|ESTF|MEKK8|TPL2|Tpl-2|c-COT 10 TCRsignalingPathway

MAP3K14 9020 mitogen-activated protein kinase kinase kinase 14 FTDCR1B|HS|HSNIK|NIK 17 TCRsignalingPathway

PDCD1 5133 programmed cell death 1 CD279|PD-1|PD1|SLEB2|hPD-1|hPD-l|hSLE1 2 TCRsignalingPathway

CTLA4 1493 cytotoxic T-lymphocyte associated protein 4 ALPS5|CD|CD152|CELIAC3|CTLA-4|GRD4|GSE|IDDM12 2 TCRsignalingPathway

PTPN6 5777 protein tyrosine phosphatase non-receptor type 6 HCP|HCPH|HPTP1C|PTP-1C|SH-PTP1|SHP-1|SHP-1L|SHP1 12 TCRsignalingPathway

CBLC 23624 Cbl proto-oncogene C CBL-3|CBL-SL|RNF57 19 TCRsignalingPathway

CBL 867 Cbl proto-oncogene C-CBL|CBL2|FRA11B|NSLL|RNF55 11 TCRsignalingPathway

CBLB 868 Cbl proto-oncogene B Cbl-b|Nbla00127|RNF56 3 TCRsignalingPathway

IL2 3558 interleukin 2 IL-2|TCGF|lymphokine 4 TCRsignalingPathway

IL4 3565 interleukin 4 BCGF-1|BCGF1|BSF-1|BSF1|IL-4 5 TCRsignalingPathway

IL5 3567 interleukin 5 EDF|IL-5|TRF 5 TCRsignalingPathway

IL10 3586 interleukin 10 CSIF|GVHDS|IL-10|IL10A|TGIF 1 TCRsignalingPathway

IFNG 3458 interferon gamma IFG|IFI 12 TCRsignalingPathway

CSF2 1437 colony stimulating factor 2 CSF|GMCSF 5 TCRsignalingPathway

TNF 7124 tumor necrosis factor DIF|TNF-alpha|TNFA|TNFSF2|TNLG1F 6 TCRsignalingPathway

CDK4 1019 cyclin dependent kinase 4 CMM3|PSK-J3 12 TCRsignalingPathway

RASGRP1 10125 RAS guanyl releasing protein 1 CALDAG-GEFI|CALDAG-GEFII|IMD64|RASGRP 15 TCRsignalingPathway

PDK1 5163 pyruvate dehydrogenase kinase 1 - 2 TCRsignalingPathway

PLCG1 5335 phospholipase C gamma 1 NCKAP3|PLC-II|PLC1|PLC148|PLCgamma1 20 TCRsignalingPathway

PRKCQ 5588 protein kinase C theta PRKCT|nPKC-theta 10 TCRsignalingPathway

TRAC 28755 T cell receptor alpha constant IMD7|TCRA|TRA|TRCA 14 TCRsignalingPathway

TRAJ1 28754 T cell receptor alpha joining 1 (non-functional) - 14 TCRsignalingPathway

TRAJ2 28753 T cell receptor alpha joining 2 (non-functional) - 14 TCRsignalingPathway

TRAJ3 28752 T cell receptor alpha joining 3 - 14 TCRsignalingPathway

TRAJ4 28751 T cell receptor alpha joining 4 - 14 TCRsignalingPathway

TRAJ5 28750 T cell receptor alpha joining 5 - 14 TCRsignalingPathway

TRAJ6 28749 T cell receptor alpha joining 6 - 14 TCRsignalingPathway

TRAJ7 28748 T cell receptor alpha joining 7 - 14 TCRsignalingPathway

TRAJ8 28747 T cell receptor alpha joining 8 - 14 TCRsignalingPathway

TRAJ9 28746 T cell receptor alpha joining 9 - 14 TCRsignalingPathway

TRAJ10 28745 T cell receptor alpha joining 10 - 14 TCRsignalingPathway

TRAJ11 28744 T cell receptor alpha joining 11 - 14 TCRsignalingPathway

TRAJ12 28743 T cell receptor alpha joining 12 - 14 TCRsignalingPathway

TRAJ13 28742 T cell receptor alpha joining 13 - 14 TCRsignalingPathway

TRAJ14 28741 T cell receptor alpha joining 14 - 14 TCRsignalingPathway

TRAJ15 28740 T cell receptor alpha joining 15 - 14 TCRsignalingPathway

TRAJ16 28739 T cell receptor alpha joining 16 - 14 TCRsignalingPathway

TRAJ17 28738 T cell receptor alpha joining 17 - 14 TCRsignalingPathway

TRAJ18 28737 T cell receptor alpha joining 18 - 14 TCRsignalingPathway

TRAJ19 28736 T cell receptor alpha joining 19 (non-functional) - 14 TCRsignalingPathway

TRAJ20 28735 T cell receptor alpha joining 20 - 14 TCRsignalingPathway

TRAJ21 28734 T cell receptor alpha joining 21 - 14 TCRsignalingPathway

TRAJ22 28733 T cell receptor alpha joining 22 - 14 TCRsignalingPathway

TRAJ23 28732 T cell receptor alpha joining 23 - 14 TCRsignalingPathway

TRAJ24 28731 T cell receptor alpha joining 24 - 14 TCRsignalingPathway

TRAJ25 28730 T cell receptor alpha joining 25 (non-functional) - 14 TCRsignalingPathway

TRAJ26 28729 T cell receptor alpha joining 26 - 14 TCRsignalingPathway

TRAJ27 28728 T cell receptor alpha joining 27 - 14 TCRsignalingPathway

TRAJ28 28727 T cell receptor alpha joining 28 - 14 TCRsignalingPathway

TRAJ29 28726 T cell receptor alpha joining 29 - 14 TCRsignalingPathway

TRAJ30 28725 T cell receptor alpha joining 30 - 14 TCRsignalingPathway

TRAJ31 28724 T cell receptor alpha joining 31 - 14 TCRsignalingPathway

TRAJ32 28723 T cell receptor alpha joining 32 - 14 TCRsignalingPathway

TRAJ33 28722 T cell receptor alpha joining 33 - 14 TCRsignalingPathway

TRAJ34 28721 T cell receptor alpha joining 34 - 14 TCRsignalingPathway

TRAJ35 28720 T cell receptor alpha joining 35 (non-functional) - 14 TCRsignalingPathway

TRAJ36 28719 T cell receptor alpha joining 36 - 14 TCRsignalingPathway

TRAJ37 28718 T cell receptor alpha joining 37 - 14 TCRsignalingPathway

TRAJ38 28717 T cell receptor alpha joining 38 - 14 TCRsignalingPathway

TRAJ39 28716 T cell receptor alpha joining 39 - 14 TCRsignalingPathway

TRAJ40 28715 T cell receptor alpha joining 40 - 14 TCRsignalingPathway

TRAJ41 28714 T cell receptor alpha joining 41 - 14 TCRsignalingPathway

TRAJ42 28713 T cell receptor alpha joining 42 - 14 TCRsignalingPathway

TRAJ43 28712 T cell receptor alpha joining 43 - 14 TCRsignalingPathway

TRAJ44 28711 T cell receptor alpha joining 44 - 14 TCRsignalingPathway

TRAJ45 28710 T cell receptor alpha joining 45 - 14 TCRsignalingPathway

TRAJ46 28709 T cell receptor alpha joining 46 - 14 TCRsignalingPathway

TRAJ47 28708 T cell receptor alpha joining 47 - 14 TCRsignalingPathway

TRAJ48 28707 T cell receptor alpha joining 48 - 14 TCRsignalingPathway

TRAJ49 28706 T cell receptor alpha joining 49 - 14 TCRsignalingPathway

TRAJ50 28705 T cell receptor alpha joining 50 - 14 TCRsignalingPathway

TRAJ52 28703 T cell receptor alpha joining 52 - 14 TCRsignalingPathway

TRAJ53 28702 T cell receptor alpha joining 53 - 14 TCRsignalingPathway

TRAJ54 28701 T cell receptor alpha joining 54 - 14 TCRsignalingPathway

TRAJ56 28699 T cell receptor alpha joining 56 - 14 TCRsignalingPathway

TRAJ57 28698 T cell receptor alpha joining 57 - 14 TCRsignalingPathway

TRAJ58 28697 T cell receptor alpha joining 58 (non-functional) - 14 TCRsignalingPathway

TRAJ59 28696 T cell receptor alpha joining 59 (non-functional) - 14 TCRsignalingPathway

TRAJ61 28694 T cell receptor alpha joining 61 (non-functional) - 14 TCRsignalingPathway

TRAV1-1 28693 T cell receptor alpha variable 1-1 TCRAV1S1|TCRAV7S1|TRAV11 14 TCRsignalingPathway

TRAV1-2 28692 T cell receptor alpha variable 1-2 TCRAV1S2|TCRAV7S2|TRAV12 14 TCRsignalingPathway

TRAV2 28691 T cell receptor alpha variable 2 TCRAV11S1|TCRAV2S1 14 TCRsignalingPathway

TRAV3 28690 T cell receptor alpha variable 3 TCRAV16S1|TCRAV3S1 14 TCRsignalingPathway

TRAV4 28689 T cell receptor alpha variable 4 TCRAV20S1|TCRAV4S1 14 TCRsignalingPathway

TRAV5 28688 T cell receptor alpha variable 5 TCRAV15S1|TCRAV5S1 14 TCRsignalingPathway

TRAV7 28686 T cell receptor alpha variable 7 TCRAV7S1 14 TCRsignalingPathway

TRAV8-1 28685 T cell receptor alpha variable 8-1 TCRAV1S1|TCRAV8S1|TRAV81 14 TCRsignalingPathway

TRAV8-2 28684 T cell receptor alpha variable 8-2 TCRAV1S5|TCRAV8S2|TRAV82 14 TCRsignalingPathway

TRAV8-3 28683 T cell receptor alpha variable 8-3 TCRAV1S4|TCRAV8S3|TRAV83 14 TCRsignalingPathway

TRAV8-4 28682 T cell receptor alpha variable 8-4 TCRAV1S2|TCRAV8S4|TRAV84 14 TCRsignalingPathway

TRAV8-6 28680 T cell receptor alpha variable 8-6 TCRAV1S3|TCRAV8S6|TRAV86 14 TCRsignalingPathway

TRAV8-7 28679 T cell receptor alpha variable 8-7 (pseudogene) TCRAV8S7|TRAV87 14 TCRsignalingPathway

TRAV9-1 28678 T cell receptor alpha variable 9-1 TCRAV9S1|TRAV91 14 TCRsignalingPathway

TRAV9-2 28677 T cell receptor alpha variable 9-2 TCRAV22S1|TCRAV9S2|TRAV92 14 TCRsignalingPathway

TRAV10 28676 T cell receptor alpha variable 10 TCRAV10S1|TCRAV24S1 14 TCRsignalingPathway

TRAV12-1 28674 T cell receptor alpha variable 12-1 TCRAV12S1|TCRAV2S3|TRAV121 14 TCRsignalingPathway

TRAV12-2 28673 T cell receptor alpha variable 12-2 TCRAV12S2|TCRAV2S1|TRAV122 14 TCRsignalingPathway

TRAV12-3 28672 T cell receptor alpha variable 12-3 TCRAV12S3|TCRAV2S2|TRAV123 14 TCRsignalingPathway

TRAV13-1 28671 T cell receptor alpha variable 13-1 TCRAV13S1|TCRAV8S1|TRAV131 14 TCRsignalingPathway

TRAV13-2 28670 T cell receptor alpha variable 13-2 TCRAV13S2|TCRAV8S2|TRAV132 14 TCRsignalingPathway

TRAV14DV4 28669 T cell receptor alpha variable 14/delta variable 4 TCRAV6S1-hDV104S1|TRAV14/DV4|hADV14S1 14 TCRsignalingPathway

TRAV16 28667 T cell receptor alpha variable 16 TCRAV16S1|TCRAV9S1 14 TCRsignalingPathway

TRAV17 28666 T cell receptor alpha variable 17 TCRAV17S1|TCRAV3S1 14 TCRsignalingPathway

TRAV18 28665 T cell receptor alpha variable 18 TCRAV18S1 14 TCRsignalingPathway

TRAV19 28664 T cell receptor alpha variable 19 TCRAV12S1|TCRAV19S1 14 TCRsignalingPathway

TRAV20 28663 T cell receptor alpha variable 20 TCRAV20S1|TCRAV30S1 14 TCRsignalingPathway

TRAV21 28662 T cell receptor alpha variable 21 TCRAV21S1|TCRAV23S1 14 TCRsignalingPathway

TRAV22 28661 T cell receptor alpha variable 22 TCRAV13S1|TCRAV22S1 14 TCRsignalingPathway

TRAV23DV6 28660 T cell receptor alpha variable 23/delta variable 6 TCRAV17S1|TRAV23/DV6|hADV23S1 14 TCRsignalingPathway

TRAV24 28659 T cell receptor alpha variable 24 TCRAV18S1|TCRAV24S1 14 TCRsignalingPathway

TRAV25 28658 T cell receptor alpha variable 25 TCRAV25S1|TCRAV32S1 14 TCRsignalingPathway

TRAV26-1 28657 T cell receptor alpha variable 26-1 TCRAV26S1|TCRAV4S2|TRAV261 14 TCRsignalingPathway

TRAV26-2 28656 T cell receptor alpha variable 26-2 TCRAV26S2|TCRAV4S1|TRAV262 14 TCRsignalingPathway

TRAV27 28655 T cell receptor alpha variable 27 TCRAV10S1|TCRAV27S1 14 TCRsignalingPathway

TRAV29DV5 28653 T cell receptor alpha variable 29/delta variable 5 TCRA|TCRAV21S1|TRAV29/DV5|hADV29S1 14 TCRsignalingPathway

TRAV30 28652 T cell receptor alpha variable 30 TCRAV29S1|TCRAV30S1 14 TCRsignalingPathway

TRAV34 28648 T cell receptor alpha variable 34 TCRAV26S1|TCRAV34S1 14 TCRsignalingPathway

TRAV35 28647 T cell receptor alpha variable 35 TCRAV25S1|TCRAV35S1 14 TCRsignalingPathway

TRAV36DV7 28646 T cell receptor alpha variable 36/delta variable 7 TCRAV28S1|TRAV36/DV7|hADV36S1 14 TCRsignalingPathway

TRAV38-1 28644 T cell receptor alpha variable 38-1 TCRAV14S2|TCRAV38S1|TRAV381 14 TCRsignalingPathway

TRAV38-2DV8 28643 T cell receptor alpha variable 38-2/delta variable 8 TCRAV14S1|TRAV382DV8|hADV38S2 14 TCRsignalingPathway

TRAV39 28642 T cell receptor alpha variable 39 TCRAV27S1|TCRAV39S1 14 TCRsignalingPathway

TRAV40 28641 T cell receptor alpha variable 40 TCRAV31S1|TCRAV40S1 14 TCRsignalingPathway

TRAV41 28640 T cell receptor alpha variable 41 TCRAV19S1|TCRAV41S1 14 TCRsignalingPathway

TRBC1 28639 T cell receptor beta constant 1 BV05S1J2.2|TCRB|TCRBC1 7 TCRsignalingPathway

TRBC2 28638 T cell receptor beta constant 2 TCRBC2 7 TCRsignalingPathway

TRBD1 28637 T cell receptor beta diversity 1 TCRBD1 7 TCRsignalingPathway

TRBD2 28636 T cell receptor beta diversity 2 TCRBD2 7 TCRsignalingPathway

TRBJ1-1 28635 T cell receptor beta joining 1-1 TCRBJ1S1|TRBJ11 7 TCRsignalingPathway

TRBJ1-2 28634 T cell receptor beta joining 1-2 TCRBJ1S2|TRBJ12 7 TCRsignalingPathway

TRBJ1-3 28633 T cell receptor beta joining 1-3 TCRBJ1S3|TRBJ13 7 TCRsignalingPathway

TRBJ1-4 28632 T cell receptor beta joining 1-4 TCRBJ1S4|TRBJ14 7 TCRsignalingPathway

TRBJ1-5 28631 T cell receptor beta joining 1-5 TCRBJ1S5|TRBJ15 7 TCRsignalingPathway

TRBJ1-6 28630 T cell receptor beta joining 1-6 TCRBJ1S6|TRBJ16 7 TCRsignalingPathway

TRBJ2-1 28629 T cell receptor beta joining 2-1 TCRBJ2S1|TRBJ21 7 TCRsignalingPathway

TRBJ2-2 28628 T cell receptor beta joining 2-2 TCRBJ2S2|TRBJ22 7 TCRsignalingPathway

TRBJ2-3 28626 T cell receptor beta joining 2-3 TCRBJ2S3|TRBJ23 7 TCRsignalingPathway

TRBJ2-4 28625 T cell receptor beta joining 2-4 TCRBJ2S4|TRBJ24 7 TCRsignalingPathway

TRBJ2-5 28624 T cell receptor beta joining 2-5 TCRBJ2S5|TRBJ25 7 TCRsignalingPathway

TRBJ2-6 28623 T cell receptor beta joining 2-6 TCRBJ2S6|TRBJ26 7 TCRsignalingPathway

TRBJ2-7 28622 T cell receptor beta joining 2-7 TCRBJ2S7|TRBJ27 7 TCRsignalingPathway

TRBV2 28620 T cell receptor beta variable 2 TCRBV22S1A2N1T|TCRBV2S1 7 TCRsignalingPathway

TRBV3-1 28619 T cell receptor beta variable 3-1 TCRBV3S1|TCRBV9S1A1T|TRBV31 7 TCRsignalingPathway

TRBV4-1 28617 T cell receptor beta variable 4-1 BV07S1J2.7|TCRBV4S1|TCRBV7S1A1N2T|TRBV41 7 TCRsignalingPathway

TRBV4-2 28616 T cell receptor beta variable 4-2 TCRBV4S2|TCRBV7S3A2|TCRBV7S3A2T|TRBV42 7 TCRsignalingPathway

TRBV4-3 28615 T cell receptor beta variable 4-3 TCRBV4S3|TCRBV7S2A1N4T|TRBV43 7 TCRsignalingPathway

TRBV5-1 28614 T cell receptor beta variable 5-1 TCRBV5S1|TCRBV5S1A1T|TRBV51 7 TCRsignalingPathway

TRBV5-4 28611 T cell receptor beta variable 5-4 TCRBV5S4|TCRBV5S6A3N2T|TRBV54 7 TCRsignalingPathway

TRBV5-5 28610 T cell receptor beta variable 5-5 TCRBV5S3A2T|TCRBV5S5|TRBV55 7 TCRsignalingPathway

TRBV5-6 28609 T cell receptor beta variable 5-6 TCRBV5S2|TCRBV5S6|TRBV56 7 TCRsignalingPathway

TRBV5-7 28608 T cell receptor beta variable 5-7 (non-functional) TCRBV5S7|TCRBV5S7P|TRBV57 7 TCRsignalingPathway

TRBV5-8 28607 T cell receptor beta variable 5-8 TCRBV5S4A2T|TCRBV5S8|TRBV58 7 TCRsignalingPathway

TRBV6-1 28606 T cell receptor beta variable 6-1 TCRBV13S3|TCRBV6S1|TRBV61 7 TCRsignalingPathway

TRBV6-2 28605 T cell receptor beta variable 6-2 TCRBV13S2|TCRBV13S2A1T|TCRBV6S2|TRBV62 7 TCRsignalingPathway

TRBV6-3 28604 T cell receptor beta variable 6-3 TCRBV13S9/13S2A1T|TCRBV6S3|TRBV63 7 TCRsignalingPathway

TRBV6-4 28603 T cell receptor beta variable 6-4 TCRBV13S5|TCRBV6S4|TRBV64 7 TCRsignalingPathway

TRBV6-5 28602 T cell receptor beta variable 6-5 TCRBV13S1|TCRBV6S5|TRBV65 7 TCRsignalingPathway

TRBV6-6 28601 T cell receptor beta variable 6-6 TCRBV13S6A2T|TCRBV6S6|TRBV66 7 TCRsignalingPathway

TRBV6-7 28600 T cell receptor beta variable 6-7 (non-functional) TCRBV13S8P|TCRBV6S7|TRBV67 7 TCRsignalingPathway

TRBV6-8 28599 T cell receptor beta variable 6-8 TCRBV13S7P|TCRBV6S8|TRBV68 7 TCRsignalingPathway

TRBV6-9 28598 T cell receptor beta variable 6-9 TCRBV13S4|TCRBV6S9|TRBV69 7 TCRsignalingPathway

TRBV7-2 28596 T cell receptor beta variable 7-2 TCRBV6S5A1N1|TCRBV6S5A2|TCRBV7S2|TRBV72 7 TCRsignalingPathway

TRBV7-3 28595 T cell receptor beta variable 7-3 TCRBV6S1A1N1|TCRBV7S3|TRBV73 7 TCRsignalingPathway

TRBV7-4 28594 T cell receptor beta variable 7-4 TCRBV6S8A2T|TCRBV7S4|TRBV74 7 TCRsignalingPathway

TRBV7-6 28592 T cell receptor beta variable 7-6 TCRBV6S3A1N1T|TCRBV7S6|TRBV76 7 TCRsignalingPathway

TRBV7-7 28591 T cell receptor beta variable 7-7 TCRBV6S6A2T|TCRBV7S7|TRBV77 7 TCRsignalingPathway

TRBV7-8 28590 T cell receptor beta variable 7-8 TCRBV6S2A1N1T|TCRBV7S8|TRBV78 7 TCRsignalingPathway

TRBV7-9 28589 T cell receptor beta variable 7-9 TCRB|TCRBV6S4A1|TCRBV7S9|TRBV79 7 TCRsignalingPathway

TRBV9 28586 T cell receptor beta variable 9 TCRBV1S1A1N1|TCRBV9S1 7 TCRsignalingPathway

TRBV10-1 28585 T cell receptor beta variable 10-1 TCRBV10S1|TCRBV12S2|TCRBV12S2A1T|TRBV101 7 TCRsignalingPathway

TRBV10-2 28584 T cell receptor beta variable 10-2 TCRBV10S2|TCRBV12S3|TRBV102 7 TCRsignalingPathway

TRBV10-3 28583 T cell receptor beta variable 10-3 TCRBV10S3|TCRBV12S1A1N2|TRBV103 7 TCRsignalingPathway

TRBV11-1 28582 T cell receptor beta variable 11-1 TCRBV11S1|TCRBV21S1|TRBV111 7 TCRsignalingPathway

TRBV11-2 28581 T cell receptor beta variable 11-2 TCRBV11S2|TCRBV21S3A2N2T|TRBV112 7 TCRsignalingPathway

TRBV11-3 28580 T cell receptor beta variable 11-3 TCRBV11S3|TCRBV21S2A2|TRBV113 7 TCRsignalingPathway

TRBV12-3 28577 T cell receptor beta variable 12-3 TCRBV12S3|TCRBV8S1|TRBV123 7 TCRsignalingPathway

TRBV12-4 28576 T cell receptor beta variable 12-4 TCRBV12S4|TCRBV8S2A1T|TRBV124 7 TCRsignalingPathway

TRBV12-5 28575 T cell receptor beta variable 12-5 TCRBV12S5|TCRBV8S3|TRBV125 7 TCRsignalingPathway

TRBV13 28574 T cell receptor beta variable 13 TCRBV13S1|TCRBV23S1A2T 7 TCRsignalingPathway

TRBV14 28573 T cell receptor beta variable 14 TCRBV14S1|TCRBV16S1A1N1 7 TCRsignalingPathway

TRBV15 28572 T cell receptor beta variable 15 TCRBV15S1|TCRBV24S1A3T 7 TCRsignalingPathway

TRBV16 28571 T cell receptor beta variable 16 BV25S1J1.2|TCRB|TCRBV16S1|TCRBV25S1|TCRBV25S1A2PT 7 TCRsignalingPathway

TRBV17 28570 T cell receptor beta variable 17 (non-functional) TCRBV17S1|TCRBV26S1P 7 TCRsignalingPathway

TRBV18 28569 T cell receptor beta variable 18 TCRBV18S1 7 TCRsignalingPathway

TRBV19 28568 T cell receptor beta variable 19 TCRBV17S1A1T|TCRBV19S1 7 TCRsignalingPathway

TRBV20-1 28567 T cell receptor beta variable 20-1 TCRBV20S1|TCRBV2S1|TRBV201 7 TCRsignalingPathway

TRBV24-1 28563 T cell receptor beta variable 24-1 TCRBV15S1|TCRBV24S1|TRBV241 7 TCRsignalingPathway

TRBV25-1 28562 T cell receptor beta variable 25-1 TCRBV11S1A1T|TCRBV25S1|TRBV251 7 TCRsignalingPathway

TRBV27 28560 T cell receptor beta variable 27 TCRBV14S1|TCRBV27S1 7 TCRsignalingPathway

TRBV28 28559 T cell receptor beta variable 28 TCRBV28S1|TCRBV3S1 7 TCRsignalingPathway

TRBV29-1 28558 T cell receptor beta variable 29-1 TCRBV29S1|TCRBV4S1A1T|TRBV291 7 TCRsignalingPathway

TRBV30 28557 T cell receptor beta variable 30 TCRBV20S1A1N2|TCRBV30S1 7 TCRsignalingPathway

TRDC 28526 T cell receptor delta constant TCRD 14 TCRsignalingPathway

TRDD1 28525 T cell receptor delta diversity 1 - 14 TCRsignalingPathway

TRDD2 28524 T cell receptor delta diversity 2 - 14 TCRsignalingPathway

TRDD3 28523 T cell receptor delta diversity 3 TCRD 14 TCRsignalingPathway

TRDJ1 28522 T cell receptor delta joining 1 TCRD 14 TCRsignalingPathway

TRDJ2 28521 T cell receptor delta joining 2 - 14 TCRsignalingPathway

TRDJ3 28520 T cell receptor delta joining 3 - 14 TCRsignalingPathway

TRDJ4 28519 T cell receptor delta joining 4 - 14 TCRsignalingPathway

TRDV1 28518 T cell receptor delta variable 1 hDV101S1 14 TCRsignalingPathway

TRDV2 28517 T cell receptor delta variable 2 hDV102S1 14 TCRsignalingPathway

TRDV3 28516 T cell receptor delta variable 3 hDV103S1 14 TCRsignalingPathway

TRGV9 6983 T cell receptor gamma variable 9 TCRGV9|TRGC1|V2 7 TCRsignalingPathway

TRGV8 6982 T cell receptor gamma variable 8 TCRGV8|V1S8 7 TCRsignalingPathway

TRGV5 6978 T cell receptor gamma variable 5 TCRGV5|V1S5 7 TCRsignalingPathway

TRGV4 6977 T cell receptor gamma variable 4 TCRGV4|V1S4 7 TCRsignalingPathway

TRGV3 6976 T cell receptor gamma variable 3 TCRGV3|V1S3 7 TCRsignalingPathway

TRGV2 6974 T cell receptor gamma variable 2 TCRGV2|VIS2 7 TCRsignalingPathway

TRGJP2 6972 T cell receptor gamma joining P2 JP2|TCRGJP2 7 TCRsignalingPathway

TRGJP1 6971 T cell receptor gamma joining P1 JP1|TCRGJP1 7 TCRsignalingPathway

TRGJP 6970 T cell receptor gamma joining P JP|TCRGJP 7 TCRsignalingPathway

TRGJ2 6969 T cell receptor gamma joining 2 J2|TCRGJ2 7 TCRsignalingPathway

TRGJ1 6968 T cell receptor gamma joining 1 J1|TCRGJ1 7 TCRsignalingPathway

TRGC2 6967 T cell receptor gamma constant 2 TCRGC2|TRGC2(2X)|TRGC2(3X) 7 TCRsignalingPathway

TRGC1 6966 T cell receptor gamma constant 1 C1|TCRG|TCRGC1 7 TCRsignalingPathway

TRAV6 6956 T cell receptor alpha variable 6 TCRAV5S1|TCRAV6S1 14 TCRsignalingPathway

BMP1 649 bone morphogenetic protein 1 OI13|PCOLC|PCP|PCP2|TLD 8 TGFb_Family_Member

BMP10 27302 bone morphogenetic protein 10 - 2 TGFb_Family_Member

BMP15 9210 bone morphogenetic protein 15 GDF9B|ODG2|POF4 X TGFb_Family_Member

BMP2 650 bone morphogenetic protein 2 BDA2|BMP2A|SSFSC 20 TGFb_Family_Member

BMP3 651 bone morphogenetic protein 3 BMP-3A 4 TGFb_Family_Member

BMP4 652 bone morphogenetic protein 4 BMP2B|BMP2B1|MCOPS6|OFC11|ZYME 14 TGFb_Family_Member

BMP5 653 bone morphogenetic protein 5 - 6 TGFb_Family_Member

BMP6 654 bone morphogenetic protein 6 VGR|VGR1 6 TGFb_Family_Member

BMP7 655 bone morphogenetic protein 7 OP-1 20 TGFb_Family_Member

BMP8A 353500 bone morphogenetic protein 8a OP-2 1 TGFb_Family_Member

BMP8B 656 bone morphogenetic protein 8b BMP8|OP2 1 TGFb_Family_Member

GDF1 2657 growth differentiation factor 1 CERS1|CHTD6|DORV|DTGA3|LAG1|LASS1|RAI|UOG1 19 TGFb_Family_Member

GDF10 2662 growth differentiation factor 10 BIP|BMP-3b|BMP3B 10 TGFb_Family_Member

GDF11 10220 growth differentiation factor 11 BMP-11|BMP11 12 TGFb_Family_Member

GDF15 9518 growth differentiation factor 15 GDF-15|MIC-1|MIC1|NAG-1|PDF|PLAB|PTGFB 19 TGFb_Family_Member

GDF2 2658 growth differentiation factor 2 BMP-9|BMP9|HHT5 10 TGFb_Family_Member

GDF3 9573 growth differentiation factor 3 KFS3|MCOP7|MCOPCB6 12 TGFb_Family_Member

GDF5 8200 growth differentiation factor 5 BDA1C|BMP-14|BMP14|CDMP1|DUPANS|LAP-4|LAP4|OS5|SYM1B|SYNS2 20 TGFb_Family_Member

GDF6 392255 growth differentiation factor 6 BMP-13|BMP13|CDMP2|KFM|KFS|KFS1|KFSL|SGM1|SYNS4 8 TGFb_Family_Member

GDF7 151449 growth differentiation factor 7 BMP12 2 TGFb_Family_Member

GDF9 2661 growth differentiation factor 9 POF14 5 TGFb_Family_Member

GDNF 2668 glial cell derived neurotrophic factor ATF|ATF1|ATF2|HFB1-GDNF|HSCR3 5 TGFb_Family_Member

INHA 3623 inhibin subunit alpha - 2 TGFb_Family_Member

INHBA 3624 inhibin subunit beta A EDF|FRP 7 TGFb_Family_Member

INHBB 3625 inhibin subunit beta B - 2 TGFb_Family_Member

INHBC 3626 inhibin subunit beta C IHBC 12 TGFb_Family_Member

INHBE 83729 inhibin subunit beta E - 12 TGFb_Family_Member

LEFTY1 10637 left-right determination factor 1 LEFTB|LEFTYB 1 TGFb_Family_Member

LEFTY2 7044 left-right determination factor 2 EBAF|LEFTA|LEFTYA|TGFB4 1 TGFb_Family_Member

NODAL 4838 nodal growth differentiation factor HTX5 10 TGFb_Family_Member

TGFB1 7040 transforming growth factor beta 1 CED|DPD1|IBDIMDE|LAP|TGF-beta1|TGFB|TGFbeta 19 TGFb_Family_Member

TGFB2 7042 transforming growth factor beta 2 G-TSF|LDS4|TGF-beta2 1 TGFb_Family_Member

TGFB3 7043 transforming growth factor beta 3 ARVD|ARVD1|LDS5|RNHF|TGF-beta3 14 TGFb_Family_Member

ACVR1B 91 activin A receptor type 1B ACTRIB|ACVRLK4|ALK4|SKR2 12 TGFb_Family_Member_Receptor

ACVR1C 130399 activin A receptor type 1C ACVRLK7|ALK7 2 TGFb_Family_Member_Receptor

ACVR2A 92 activin A receptor type 2A ACTRII|ACVR2 2 TGFb_Family_Member_Receptor

ACVR2B 93 activin A receptor type 2B ACTRIIB|ActR-IIB|HTX4 3 TGFb_Family_Member_Receptor

ACVRL1 94 activin A receptor like type 1 ACVRLK1|ALK-1|ALK1|HHT|HHT2|ORW2|SKR3|TSR-I 12 TGFb_Family_Member_Receptor

AMHR2 269 anti-Mullerian hormone receptor type 2 AMHR|MISR2|MISRII|MRII 12 TGFb_Family_Member_Receptor

BMPR1A 657 bone morphogenetic protein receptor type 1A 10q23del|ACVRLK3|ALK3|CD292|SKR5 10 TGFb_Family_Member_Receptor

BMPR1B 658 bone morphogenetic protein receptor type 1B ALK-6|ALK6|AMDD|BDA1D|BDA2|CDw293 4 TGFb_Family_Member_Receptor

BMPR2 659 bone morphogenetic protein receptor type 2 BMPR-II|BMPR3|BMR2|BRK-3|POVD1|PPH1|T-ALK 2 TGFb_Family_Member_Receptor

TGFBR1 7046 transforming growth factor beta receptor 1 AAT5|ACVRLK4|ALK-5|ALK5|ESS1|LDS1|LDS1A|LDS2A|MSSE|SKR4|TBR-i|TBRI|TGFR-1|tbetaR-I 9 TGFb_Family_Member_Receptor

TGFBR2 7048 transforming growth factor beta receptor 2 AAT3|FAA3|LDS1B|LDS2|LDS2B|MFS2|RIIC|TAAD2|TBR-ii|TBRII|TGFR-2|TGFbeta-RII 3 TGFb_Family_Member_Receptor

TGFBR3 7049 transforming growth factor beta receptor 3 BGCAN|betaglycan 1 TGFb_Family_Member_Receptor

TNFRSF11B 4982 TNF receptor superfamily member 11b OCIF|OPG|PDB5|TR1 8 TNF_Family_Members

TNFSF10 8743 TNF superfamily member 10 APO2L|Apo-2L|CD253|TL2|TNLG6A|TRAIL 3 TNF_Family_Members

TNFSF11 8600 TNF superfamily member 11 CD254|ODF|OPGL|OPTB2|RANKL|TNLG6B|TRANCE|hRANKL2|sOdf 13 TNF_Family_Members

TNFSF12 8742 TNF superfamily member 12 APO3L|DR3LG|TNLG4A|TWEAK 17 TNF_Family_Members

TNFSF13 8741 TNF superfamily member 13 APRIL|CD256|TALL-2|TALL2|TNLG7B|TRDL-1|UNQ383/PRO715|ZTNF2 17 TNF_Family_Members

TNFSF13B 10673 TNF superfamily member 13b BAFF|BLYS|CD257|DTL|TALL-1|TALL1|THANK|TNFSF20|TNLG7A|ZTNF4 13 TNF_Family_Members

TNFSF14 8740 TNF superfamily member 14 CD258|HVEML|LIGHT|LTg 19 TNF_Family_Members

TNFSF15 9966 TNF superfamily member 15 TL1|TL1A|TNLG1B|VEGI|VEGI192A 9 TNF_Family_Members

TNFSF18 8995 TNF superfamily member 18 AITRL|GITRL|TL6|TNLG2A|hGITRL 1 TNF_Family_Members

TNFSF4 7292 TNF superfamily member 4 CD134L|CD252|GP34|OX-40L|OX4OL|TNLG2B|TXGP1 1 TNF_Family_Members

TNFSF8 944 TNF superfamily member 8 CD153|CD30L|CD30LG|TNLG3A 9 TNF_Family_Members

TNFSF9 8744 TNF superfamily member 9 4-1BB-L|CD137L|TNLG5A 19 TNF_Family_Members

TNFRSF10B 8795 TNF receptor superfamily member 10b CD262|DR5|KILLER|KILLER/DR5|TRAIL-R2|TRAILR2|TRICK2|TRICK2A|TRICK2B|TRICKB|ZTNFR9 8 TNF_Family_Members_Receptors

TNFRSF10C 8794 TNF receptor superfamily member 10c CD263|DCR1|DCR1-TNFR|LIT|TRAIL-R3|TRAILR3|TRID 8 TNF_Family_Members_Receptors

TNFRSF10D 8793 TNF receptor superfamily member 10d CD264|DCR2|TRAIL-R4|TRAILR4|TRUNDD 8 TNF_Family_Members_Receptors

TNFRSF11A 8792 TNF receptor superfamily member 11a CD265|FEO|LOH18CR1|ODFR|OFE|OPTB7|OSTS|PDB2|RANK|TRANCER 18 TNF_Family_Members_Receptors

TNFRSF12A 51330 TNF receptor superfamily member 12A CD266|FN14|TWEAKR 16 TNF_Family_Members_Receptors

TNFRSF13B 23495 TNF receptor superfamily member 13B CD267|CVID|CVID2|IGAD2|RYZN|TACI|TNFRSF14B 17 TNF_Family_Members_Receptors

TNFRSF13C 115650 TNF receptor superfamily member 13C BAFF-R|BAFFR|BROMIX|CD268|CVID4|prolixin 22 TNF_Family_Members_Receptors

TNFRSF14 8764 TNF receptor superfamily member 14 ATAR|CD270|HVEA|HVEM|LIGHTR|TR2 1 TNF_Family_Members_Receptors

TNFRSF17 608 TNF receptor superfamily member 17 BCM|BCMA|CD269|TNFRSF13A 16 TNF_Family_Members_Receptors

TNFRSF18 8784 TNF receptor superfamily member 18 AITR|CD357|ENERGEN|GITR|GITR-D 1 TNF_Family_Members_Receptors

TNFRSF19 55504 TNF receptor superfamily member 19 TAJ|TAJ-alpha|TRADE|TROY 13 TNF_Family_Members_Receptors

TNFRSF1A 7132 TNF receptor superfamily member 1A CD120a|FPF|TBP1|TNF-R|TNF-R-I|TNF-R55|TNFAR|TNFR1|TNFR55|TNFR60|p55|p55-R|p60 12 TNF_Family_Members_Receptors

TNFRSF1B 7133 TNF receptor superfamily member 1B CD120b|TBPII|TNF-R-II|TNF-R75|TNFBR|TNFR1B|TNFR2|TNFR80|p75|p75TNFR 1 TNF_Family_Members_Receptors

TNFRSF21 27242 TNF receptor superfamily member 21 BM-018|CD358|DR6 6 TNF_Family_Members_Receptors

TNFRSF25 8718 TNF receptor superfamily member 25 APO-3|DDR3|DR3|GEF720|LARD|PLEKHG5|TNFRSF12|TR3|TRAMP|WSL-1|WSL-LR 1 TNF_Family_Members_Receptors

TNFRSF4 7293 TNF receptor superfamily member 4 ACT35|CD134|IMD16|OX40|TXGP1L 1 TNF_Family_Members_Receptors

TNFRSF6B 8771 TNF receptor superfamily member 6b DCR3|DJ583P15.1.1|M68|M68E|TR6 20 TNF_Family_Members_Receptors

TNFRSF8 943 TNF receptor superfamily member 8 CD30|D1S166E|Ki-1 1 TNF_Family_Members_Receptors

TNFRSF9 3604 TNF receptor superfamily member 9 4-1BB|CD137|CDw137|ILA 1 TNF_Family_Members_Receptors
